# Peripheral immune profiles separate disease activity stages in birdshot uveitis

**DOI:** 10.3389/fimmu.2026.1938305

**Published:** 2026-10-02

**Authors:** D. Pohlmann-Krappitz, I. Kaeferstein, B. Kruse, S. Winterhalter, A. Thiel, U. Pleyer, J. Braun

**Affiliations:** 1 Berlin Institute of Health at Charité – Universitätsmedizin Berlin, Berlin, Germany; 2 Department of Ophthalmology, Charité – Universitätsmedizin Berlin, corporate member of Freie Universität Berlin, Humboldt-Universität zu Berlin, and Berlin Institute of Health, Berlin, Germany; 3 BIH Center of Immunomics, BIH, Berlin Institute of Health at Charité - Universitätsmedizin Berlin, Berlin, Germany; 4 Si-M/”Der Simulierte Mensch” a science framework of Technische Universität Berlin and Charité - Universitätsmedizin Berlin, Berlin, Germany

**Keywords:** birdshot uveitis, mass cytometry, peripheral immune signatures, T cells, uveitis posterior

## Abstract

**Introduction:**

Birdshot uveitis (BU) is a chronic, debilitating posterior uveitis strongly associated with HLA-A*29, supporting a suspected autoimmune etiology. We used high-dimensional mass cytometry and clinical stratification to characterize the peripheral immune landscape in BU and identify disease activity-dependent signatures.

**Methods:**

In this cross-sectional study, we included 36 treated BU patients and 31 age- and sex-matched healthy controls (HCs). All participants underwent comprehensive ophthalmologic evaluation with multimodal imaging. Patients were stratified by an expert ophthalmologist into Inactive, Active, and Burned-out disease activity groups based on clinical and imaging findings. Peripheral blood and serum were collected for immunoprofiling using a 37-marker mass cytometry panel and multiplex cytokine analysis.

**Results:**

Compared with HCs, BU patients showed increased frequencies of Th17 cells, CD146^+^ T cells, CXCR3^+^CCR4^+^ intermediate effector/central memory T cells, CD56^dim^ natural killer (NK) cells, and elevated serum IL-18. Disease activity-specific differences were evident. Inactive patients displayed more quiescent effector T cell phenotypes, including higher frequencies of TIM-3^+^ Tc17-Tc22 intermediates and CD8^+^ T memory stem cells (T_SCM_), capable of maintaining long-term immune and autoimmune memory. Active patients exhibited increased *in vivo* activation of Th17 and Tc1 subsets (elevated HLA-DR, CD38, and PD-1 expression) and the highest levels of CD56^dim^ NK cells. Immune profiles were also linked to current treatment: conventional synthetic disease-modifying antirheumatic drugs (csDMARDs) were associated with higher CD56^bright^ NK cell frequencies, while absence of current therapy correlated with elevated PD-1^+^/SLAMF7^+^ Tc17+1 and PD-1^+^ CD57^+^ CD8^+^ terminally differentiated effector memory T cells re-expressing CD45RA (T_EMRA_). Tocilizumab treatment resulted in apparent loss of IL-6R^+^ T cells and an increase in SLAMF7^+^ T cells, consistent with epitope masking by the therapeutic antibody.

**Discussion:**

Integrated peripheral immune profiling with clinical stratification revealed distinct disease activity-dependent and treatment-associated signatures in BU. Inactive and Burned-out patients showed predominantly exhausted or anergic T cell phenotypes, whereas active disease featured heightened T cell activation and mature NK cell expansion. These findings demonstrate both the value and challenges of interpreting immune profiles in treated patients, especially with biologics such as tocilizumab that alter receptor detectability. Despite the heterogeneity typical of rare-disease cohorts, activity-associated immune patterns were detectable. Future studies should evaluate the diagnostic, prognostic, and monitoring utility of these signatures in BU.

## Introduction

Birdshot uveitis (BU), also known as birdshot chorioretinopathy, represents a rare, non-infectious posterior uveitis characterized by recurrent episodes of inflammation affecting the retina and stromal choroid, with a “shotgun pattern” appearance and ovoid lesions around the optic nerve and mid-periphery of the retina (1). Patients experience floaters, blurred vision, and photopsia, and may encounter reduced visual acuity (VA), nocturnal blindness, and color blindness, resulting from retinal and macular degeneration, extensive choroidal depigmentation, and progressive formation of scar tissue on the retina (2).

BU is highly associated with the major histocompatibility complex (MHC) gene human leukocyte antigen class I allele A*29 (HLA-A*29) and different variants of the endoplasmic reticulum aminopeptidase 2 (ERAP2) gene (3–5). Endoplasmic reticulum aminopeptidase 1 (ERAP1) and ERAP2 are responsible for trimming peptides to an optimal size for binding to MHC-I molecules, which is crucial for antigen presentation to T cells (6). The combination of differential peptide processing by ERAP2 and the presence of HLA-A*29 may be pivotal for presentation of disease-associated self-peptides and molecular mimicker antigens, which break central T cell tolerance and initiate autoimmunity. Differential leukocyte migration across the inner and outer blood-retinal barrier (BRB) may contribute to the spatiotemporal activity differences and the progressive and relapsing course of BU.

In this study, we applied high-dimensional mass cytometry and multiplex cytokine profiling to comprehensively characterize peripheral immune signatures in BU (**Figure 1**). Our goal is to identify disease-specific immune alterations to find cues about disease etiology and potential biomarkers that may enhance diagnostic precision and guide individualized immunotherapy.

**Figure 1:**
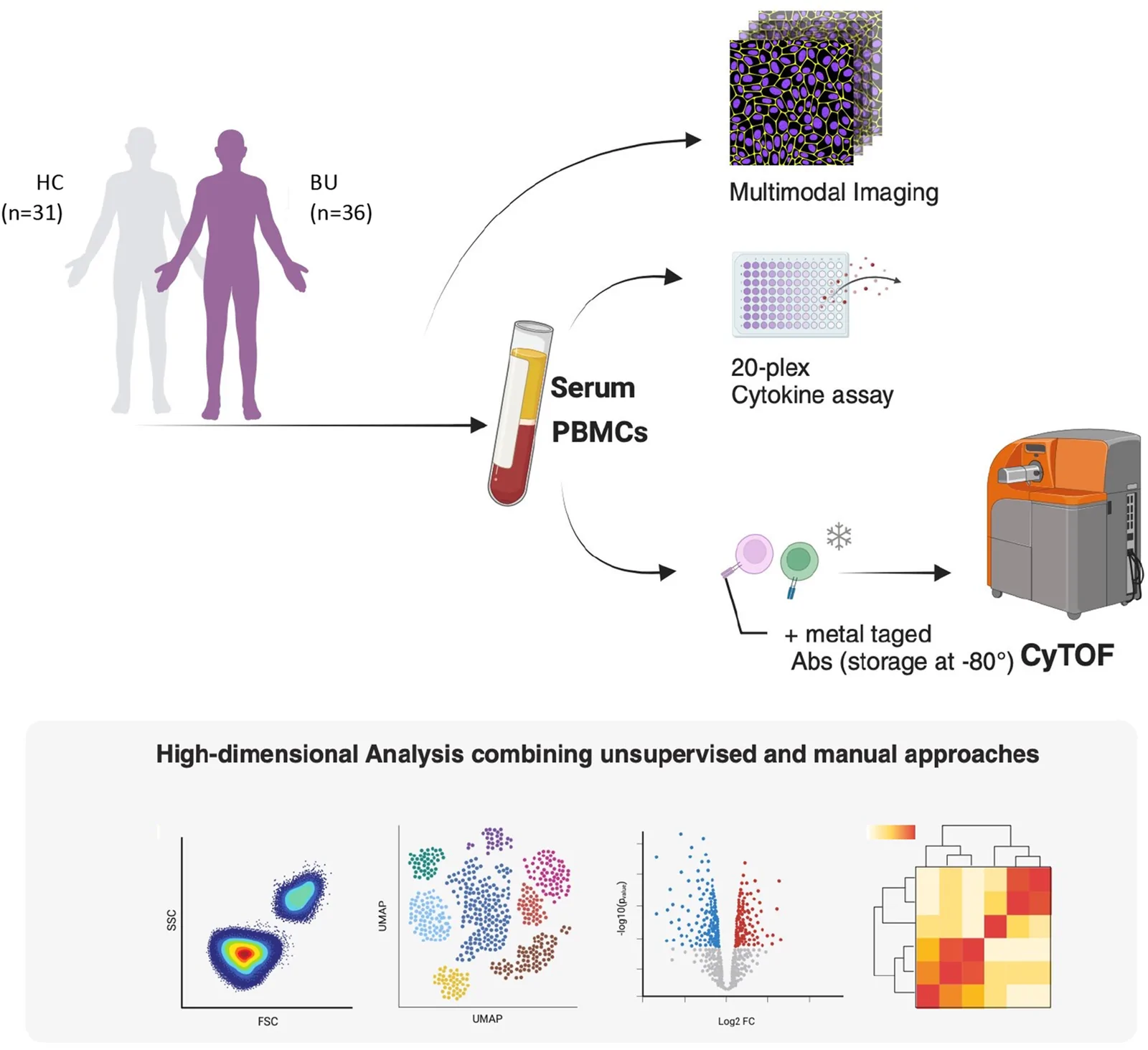
Study overview Peripheral blood and serum were collected from birdshot uveitis (BU) patients (n=36) and healthy controls (HC, n=31). Multimodal ocular imaging was performed and PBMCs were stained with metal-tagged antibodies and acquired by mass cytometry (CyTOF). In addition, serum samples were analyzed by a 20-plex cytokine assay. High-dimensional analyses combined complementary pipelines, including unsupervised clustering/UMAP, targeted manual gating and differential abundance testing. Created with Biorender. Created with Biorender.

## Methods

### Patients and study design

This single-center, prospective study was performed in accordance with the Declaration of Helsinki and approved by the local ethics committee (Ethikkommission der Charité- Universitätsmedizin Berlin, EA2/066/19). Recruitment took place between January 2019 and July 2019. Before participating in the study, each donor provided written informed consent. The study investigated a total of 36 patients with BU, affecting 72 eyes, along with a control group comprising 31 healthy controls (HCs). The diagnosis of BU was made according to the research criteria of the international consensus conference (Levinson 2006) (7). All BU patients had been previously screened for positivity of the HLA-A*29:02 allele. HCs were not HLA-typed. As patient recruitment preceded publication of the 2021 Standardization of Uveitis Nomenclature (SUN) classification criteria, the Levinson criteria were retained as the prospectively applied diagnostic scheme. However, the cohort was additionally reviewed according to the 2021 SUN classification criteria, incorporating the characteristic clinical phenotype, multimodal imaging findings, and HLA-A*29 status, and all included BU patients also fulfilled these criteria.

### Clinical assessment

Comprehensive ophthalmologic examination was performed by a board-certified ophthalmologist specializing in uveitis (D.P.-K.) and included the following assessments: 1) Visual acuity (VA) using the Early Treatment Diabetic Retinopathy Study (ETDRS) chart in decimal notation and converted into logarithm of the minimum angle of resolution (logMAR), 2) Slit-lamp examination and indirect fundoscopy: The ocular inflammation was classified according to the SUN Working Group criteria (8). Vitreous haze (VH) as the degree of cellular infiltration was graded as 0 = no infiltration, +0.5 = minimal, +1 = mild, +2 = moderate, and 3 = severe according to the SUN scale. 3) High-resolution imaging of the macula and optic nerve was performed using spectral-domain optical coherence tomography (SD-OCT, SPECTRALIS^®^, Heidelberg Engineering, Heidelberg, Germany). The macula assessment comprised the average central retinal thickness (CRT), the presence of cystoid macular edema (CME), the external limiting membrane (ELM), the ellipsoid zone, the complex of the retinal pigment epithelium (RPE) and Bruch’s membrane, and the presence of an epiretinal membrane (ERM). The optic nerve head assessment included measurement of the thickness of the retinal nerve fiber layer (RNFL, around 90 to 110 µm in the peripapillary region) (9). Swelling and thinning of the RNFL were recorded as additional information for inflammation or degeneration. 4) Fundus autofluorescence (FAF) was performed with BluePeak™ blue laser and infrared reflectance by SD-OCT (SPECTRALIS^®^, Heidelberg Engineering, Heidelberg, Germany). The following fluorescent patterns were assessed: a) Healthy RPE cells typically show a background level of autofluorescence; b) Areas with increased lipofuscin accumulation appear as brighter spots; c) Areas of RPE atrophy or damage may exhibit reduced or absent autofluorescence (10, 11). 5) Fluorescein angiography (FA) was performed after intravenous injection of 5 mL of 20% sodium fluorescein to visualize retinal and macular leakage. 6) Indocyanine green angiography (ICGA) and FA were performed simultaneously on the SD-OCT (SPECTRALIS^®^, Heidelberg Engineering, Heidelberg, Germany). For ICGA, 500 mg indocyanine green (ICG) (Cardiogreen; Paesel + Lorei GmbH & Co. KG, Rheinberg, Germany) diluted in 7.5 mL of 0.9% saline solution was administered intravenously. Findings from both FA and ICGA were graded based on Tugal-Tutkun et al. to quantify the active intraocular inflammation at the posterior pole (12), including hyperfluorescence of the optic disc, CME, staining of retinal blood vessels, and/or the presence of leakage from superficial vessels and the deep capillary plexus. Macular leakage was assessed using the grading system introduced by Yannuzzi (13). All assessments were based on observations made during the late phases of angiograms. Additionally, early-phase (<3 minutes), intermediate-phase (10 minutes), and late-phase (28–32 minutes) angiograms following ICGA injection were examined for signs of choroidal inflammation, such as the presence of hypofluorescent dots, fuzzy and ill-defined choroidal vessels, and late-diffuse choroidal hyperfluorescence. Areas displaying atrophy were specifically excluded from consideration in these analyses. 7) Fundus photography for the evaluation of choroidal lesions was performed using ZEISS FF450plus (Carl Zeiss Meditec AG, Jena, Germany). Clinical activity grading was performed before analysis of immunological data. Clinical stratification was based on the integrated assessment of both eyes. No patient showed clearly discordant inflammatory activity states between the two eyes, as bilateral involvement was generally concordant. In cases of minor inter-eye asymmetry, the eye with the more pronounced inflammatory findings informed the patient-level classification. Thus, no predefined study-eye or averaging approach was required. No study-specific numerical cutoffs were introduced for FA, ICGA, or OCT. Inflammatory activity was defined according to the presence and established grading of the above-mentioned angiographic features, whereas OCT parameters were used to characterize inflammatory complications and cumulative structural damage.

### Blood sampling and cell isolation

40 mL heparinized blood and 5 mL serum were collected, and peripheral blood mononuclear cells (PBMCs) were isolated by layering blood mixed 1:1 with phosphate-buffered saline containing bovine serum albumin (PBS/BSA) onto 10 mL Biocoll (Merck KGaA, Darmstadt, Germany) per 40 mL of blood/PBS suspension and centrifuging at 800 × g for 25 minutes at room temperature (RT). The interphase was resuspended in 50 mL of PBS/BSA and centrifuged at 310 × g for 10 minutes at RT. After a second washing step, the cell pellet was resuspended in 10 mL PBS/BSA containing ethylenediaminetetraacetic acid (EDTA). Cell counts were obtained using the CASY cell counter (Roche Innovatis, Basel, Switzerland).

### Multiplex cytokine assay

Cytokine and chemokine measurement from serum samples at a 1:4 dilution was performed using a 20-plex bead array kit (Bio-Plex Pro Human Immunotherapy 20-Plex Panel #12007975, Bio-Rad Laboratories, Inc., Hercules, CA, USA) including GM-CSF, IFN-γ, IL-2, IL-4, IL-5, IL-6, IL-7, IL-8, IL-10, IL-13, IL-15, IL-17A, IL-18, IP-10, MCP-1, MIG, MIP-1α, MIP-1β, RANTES, and TNF-α, according to the manufacturer’s instructions with a Bio-Plex Pro Wash Station and a Bio-Plex 200 system.

### Staining cells for mass cytometry

Up to 3 × 10^6^ PBMCs were transferred into individual wells and suspended in 2 mL CyFACS (PBS/BSA/2 mM EDTA/0.05% Natrium azide). After centrifugation at 310 × g for 7 minutes at RT, the supernatant was removed. Antibody-MasterMix was thawed and filtered through a 0.1 µm centrifugation column. The cell pellet was incubated with 50 µL of MasterMix at RT for 20 minutes. Dead cells were labeled with 5 µM cisplatin for an additional 10 minutes at RT. Staining was terminated by washing twice with 2 mL CyFACS buffer and centrifuging at 310 × g for 7 minutes at RT. Subsequently, DNA staining was performed with iridium intercalator solution at a final dilution of 1:5000 in MaxPar FixPerm buffer. The cell pellet was resuspended in 1 mL of the intercalator solution and left to incubate for 60 minutes at RT. 1 mL CyFACS was added, and the suspension was centrifuged at 800 × g for 7 minutes at RT. The supernatant was removed, and cells were resuspended in 250 µL of the 1.6% paraformaldehyde (PFA) solution and incubated overnight at 4 °C.

### Cryopreservation

The following day, antibody-labeled samples were washed with 2 mL of CyFACS and centrifuged at 800 × g for 7 minutes, and cell pellets were resuspended in 900 µL fetal calf serum (FCS). Cell suspensions were transferred to cryotubes and placed in freezing containers (Mr. Frosty™, Thermo Fisher Scientific, Waltham, MA, USA), and 100 µL dimethyl sulfoxide (DMSO) was added per cryotube. Finally, the cryotubes were moved to -80 °C for overnight storage and then to liquid nitrogen for long-term storage.

### Barcoding and measuring of stained and frozen PBMCs and data processing

Frozen and stained PBMCs were gently thawed at 37 °C until nearly completely thawed and transferred to 15 mL Falcon tubes with 10 mL Medium III (80% Roswell Park Memorial Institute (RPMI) medium and 20% FCS). After centrifugation at 800 × g for 10 minutes, the supernatant was carefully aspirated and the cell pellet was resuspended in 1 mL Benzonase medium (Pierce Universal Nuclease for Cell Lysis; Thermo Fisher Scientific, Waltham, MA, USA; 25 U/mL, 1:10,000 in Medium III) and incubated for 5 minutes at 37 °C. The suspension was filled up to 10 mL with CyFACS, centrifuged at 800 × g for 10 minutes, and the supernatant was aspirated.

The samples were barcoded using the Cell-ID 20-Plex Pd Barcoding Kit (Fluidigm Corporation, South San Francisco, CA, USA) to enable sample separation after pooled measurement. Maxpar Barcode tubes were thawed for 10 minutes at RT before 1 mL Maxpar FixPerm buffer (Fluidigm Corporation, South San Francisco, CA, USA) was aliquoted per individual sample. The contents of the barcode tubes were resuspended in 100 µL Maxpar FixPerm buffer from the respective aliquots and mixed back into the original tubes. The cell pellet was resuspended in 1 mL Barcoding solution and incubated for 60 minutes at RT. The reaction was terminated by adding 1 mL of CyFACS, followed by centrifugation at 800 × g for 7 minutes and a second washing step with 2 mL CyFACS. Finally, cell pellets were resuspended in 200 µL CyFACS and a sample volume of 2 µL was taken for cell counting. Then, samples were pooled to reach a final volume of 2 mL in CyFACS and centrifuged at 800 × g for 7 minutes. After supernatant aspiration, cell pellets were resuspended in 2 mL water and centrifuged at 800 × g for 7 minutes. Cell pellets were finally resuspended in 1 mL of water. A 10 µL sample volume was taken for cell counting. The pooled samples were divided into individual wells, each containing approximately 2 × 10^6^ cells after the final wash. Samples were brought up to a volume of 2 mL with water and centrifuged at 800 × g for 7 minutes. The supernatant was aspirated to completion, and the cell pellet was preserved on ice for subsequent cytometry by time of flight (CyTOF) measurement (performed by BIH Cytometry Core Facility). To achieve a high-resolution dataset of 1 million events per sample, we pooled three samples (each containing on average 3.25 × 10^6^ cells) and acquired 3 million events on the CyTOF per pool, yielding an average of 8.67 × 10^5^ PBMCs per data file. With this comparably high event count, we were able to identify and quantify even minor T cell subsets with confidence. To prevent measurement batch effects, BU and HC samples were consistently mixed in the sample pools.

The CyTOF raw data files were EQ-normalized by the BIH Cytometry Core Facility. Further normalization steps, e.g., of label intensity, were not necessary. The exported.fcs files were compensated using the CATALYST (Cytometry dATa anALYSis Tools) R package (14), employing a compensation matrix generated by a single-stained microbead pool (OneComp eBeads Compensation Beads, Invitrogen, Carlsbad, CA, USA). The pooled files were debarcoded using the Fluidigm Processing Toolbox software (v.1.04).

### Data/statistical analysis

The compensated and debarcoded mass cytometric data files were pre-gated to exclude normalization beads, dead cells, and doublets according to the gating scheme presented in **Supplementary Figure 3**, by which CD4+ T cells, CD8+ T cells, and natural killer (NK) cells were identified. These subsets were further analyzed using two complementary approaches (**Supplementary Figures 1**-**5**). Unsupervised clustering via FlowSOM and Uniform Manifold Approximation and Projection (UMAP) identified 240 cell clusters across CD4^+^ T cells, CD8^+^ T cells, and NK cells (80 clusters per lineage) (**Supplementary Figures 1A–C**). These were aggregated into 30 memory, 38 differentiation, or 73 unique phenotypic subsets (**Supplementary Figure 2**, **Supplementary Appendix**). Comprehensive manual gating focused on resolving rare T cell populations potentially missed by clustering and retrieved 229 distinct T cell and 54 NK cell populations (**Supplementary Figures 1D** and **3**-**5**). In brief, memory T cell subsets were defined based on CD45RA and CCR7 expression: naïve T cells (T_N_, CCR7^+^ CD45RA^+^), central memory T cells (T_CM_, CCR7^+/-^ CD45RA^−^), and terminally differentiated effector memory T cells re-expressing CD45RA (T_EMRA_, CCR7 ^−^CD45RA^+^). CD4^+^ and CD8^+^ memory T cells were further divided based on chemokine receptor expression (CCR6, CXCR3, CCR4, and CCR10) into helper and cytotoxic T cell (Th/Tc) subsets: Th1/Tc1, Th2/Tc2, Th17+1/Tc17+1, Th17/Tc17, and Th22/Tc22, as described by Loyal et al. (15) These subsets were further characterized regarding CD146, PD-1, CD57, TIM-3, HLA-DR, and CD38 expression. In addition, CD56^+^ CD3^-^ NK cells were split into CD8^+/-^ CD16^+^ CD56^dim^ and CD8^+/-^ CD16^-^ CD56^bright^ NK cells. Correlation analysis of comparable T cell populations obtained either from unsupervised clustering or manual gating demonstrated consistency between both analytical approaches (**Supplementary Figure 1E**).

Statistical analysis was carried out using R (version 4.5.1) and RStudio (version 2025.05.1 + 513). To delineate immune alterations specific to BU, we compared the immune profiles of BU patients to those of HCs using log_2_ fold-change and −log_10_(false discovery rate [FDR]) obtained by Wilcoxon rank-sum tests. Differences between disease activity stages or current immunomodulatory therapy (IMT) groups were determined by ANOVA with Tukey’s *post-hoc* test and results were displayed as effect size Eta (2) and −log_10_(FDR). Correlations were evaluated using Pearson’s correlation coefficient r and p-values were reported. Group-wise comparisons were performed with Wilcoxon tests and significance levels were displayed as p-values or as asterisks (*, p ≤ 0.05; **, p ≤ 0.01; ***, p ≤ *0.001*).

## Results

### Clinical characteristics, patient stratification, and therapeutic regimens

A total of 36 patients diagnosed with BU and 31 age-matched HCs were enrolled and underwent ophthalmologic, immunologic, and serologic investigations (**Figure 1**). All BU patients were HLA-A*29:02 positive. Detailed anthropometric characteristics, treatment regimens, and clinical and ophthalmologic status are listed in **Table 1**. To illustrate differences in disease stages of BU patients, all clinical, ophthalmologic, and multimodal imaging parameters, including OCT measurements, VH, VA, and findings from FAF, ICGA, and FA were summarized in a heatmap (**Figure 2A**). In line with our previous multimodal imaging-based characterization of BU (2), patient phenotyping incorporated both inflammatory imaging features and morphological parameters reflecting cumulative structural damage. Based on FA measurements, the expert investigator characterized current inflammatory disease activity (active, inactive) in four categories, namely, activity overall (Activity FA), activity in the superficial retina (Type I), activity in the deep retina (Type II), or activity in the choroid (Type III). By integrating the ophthalmologic expert rating, we generated the fine-mapped activity score (Activity Expert) with seven stages. Most groups with active disease were too small for meaningful statistical comparisons (n < 5). Thus, for the following analyses, we compared HCs and BU patients, with BU patients stratified into active and inactive inflammatory states and a separate Burned-out phenotype. Burned-out was not considered an inflammatory activity state but was defined by advanced morphological damage and analyzed separately to enable comparison of the peripheral immune profile. We further display five different stages of retina and choroid activity according to the ‘Activity Expert’ staging. Age, disease duration, current treatment, inflammatory activity, and structural disease stage are displayed separately (**Figures 2B–E**).

**Figure 2:**
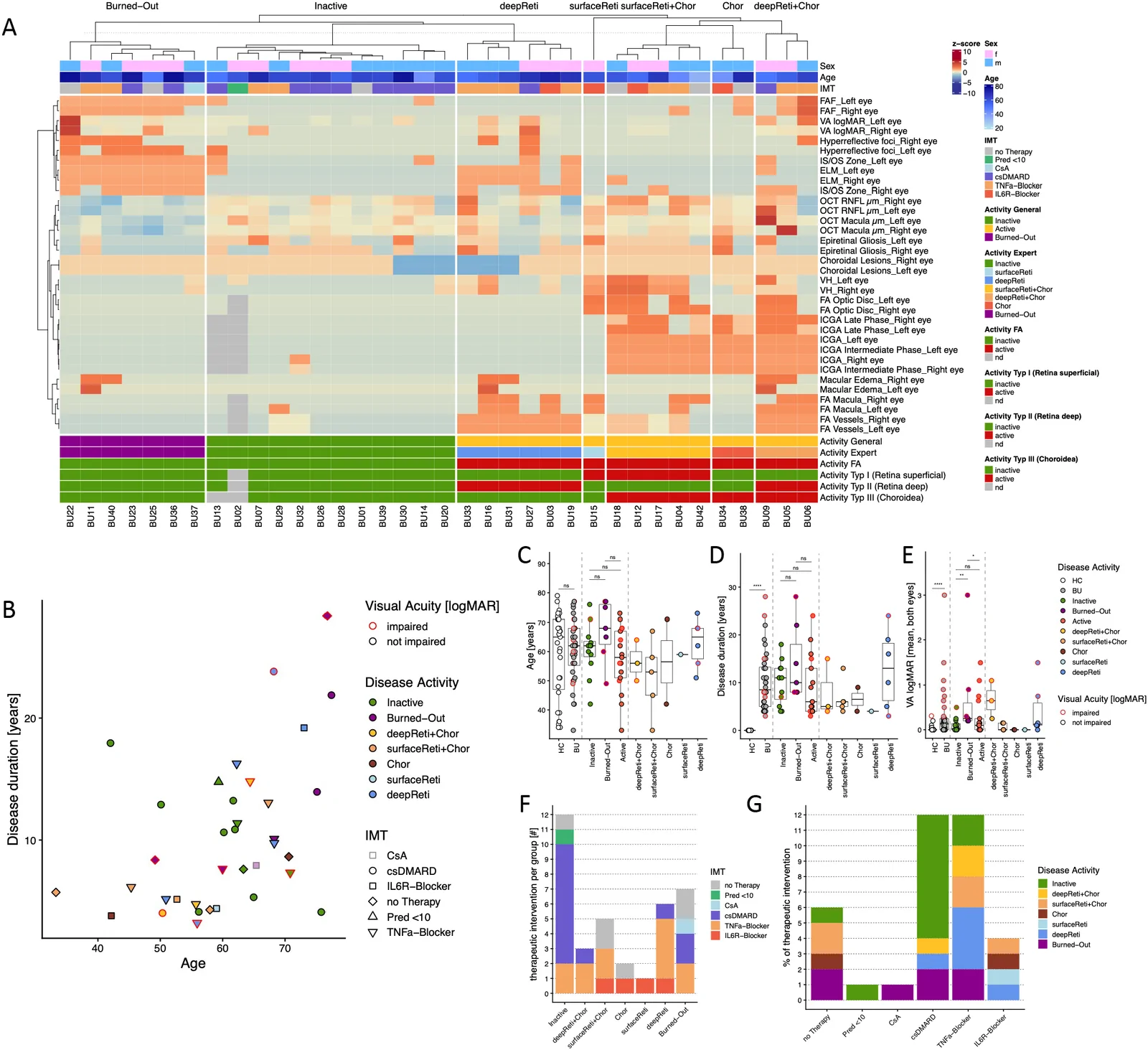
Multimodal clinical clustering of birdshot uveitis (BU) defines five disease activity groups, with accompanying age–disease duration distribution and treatment patterns across groups. **(A)**: Stratification of patients using key clinical and imaging variables. The heatmap shows clinical parameters split by expert defined activity stages. Top annotations indicate age, sex, and current immunomodulatory treatment (IMT) . Bottom annotation shows ophthalmologist-defined activity FA and different types (I = retina superficial; II = retina deep; III = choroid) based on fluorescence- and indocyaningreenangiography. These expert-defined activity gradings were summarised into Activity General with Inactive, Burned-Out and Active, which were further fine-mapped into surface Retina, surface Retina + Choroid, deep Retina, deep Retina + Choroid and Choroid (Activity Expert). **(B)**: Scatterplot of individual BU patients showing age versus disease duration. Points are color-coded by activity group and shaped by current immunosuppression; contouring in red indicates visual acuity (VA) impairment status. **(C-E)**: Box plots for age, disease duration and visual acuity with disease activity on the x-axis. Groups are HC and BU, split into Inactive, Burned-Out and Active, which were further split (indicated by dashed lines) according to activity affecting deep retina + choroid (deepReti+Chor), surface retina + choroid (surfaceReti+Chor), choroid (Chor), surface and deep retina (surfaceReti and deepReti); for colors see legend. Horizontal bars and boxes indicate medians and interquartile ranges, respectively. Groups HC, BU, Inactive, Burned-Out and Active were statistically compared using Wilcoxon test. Statistical comparisons were performed using Wilcoxon test (*, p<0.05; **, p<0.01; ****, p<0.0001) **(F, G)**: Treatment distribution by activity groups **(F)** and activity distribution by treatment **(G)** is displayed as stacked bar chart showing the number of current immunosuppressive regimens (IMT) within each group (Healthy control (HC), Inactive, Retina Activity, Retina + Choroid Activity, Choroid Activity, Burned-out).

**Table 1:** Baseline characteristics.

Parameter	BU N=36	HC N=31
Age (years) ^ns1^	60.4 (10.5)	58.9 (14.9)
Disease duration (years) [Mean (SD)]	10.3 (6.2)	–
Sex [N Female, %] ^ns2^	17 (47%)	21 (67.7%)
Current IMT [N (%)]		
Prednisolone < 10 mg	1 (3%)	–
csDMARDs (AZA, MMF, MTX)	12 (33%)	–
T cell inhibitor, CsA	1 (3%)	–
TNFalpha antagonists	12 (33%)	–
IL-6R antagonist	4 (11%)	–
None	6 (17%)	31 (100%)
Lens [Number of eyes (%)]		
Phakic	29 (40%)	40 (65%)
Cataract	22 (31%)	15 (24%)
Pseudophakic	21 (29%)	3 (5%)
NA	–	4 (6%)
OCT measurements		
CRT (µm) [Mean (SD)]	271.5 (98.3)	283.3 (30.5)
CRT (µm) [Median (range)]	267 (102 - 791)	280 (228 - 426)
Choroidal thickness (µm) [Mean (SD)]	211.1 (69.1)	–
Choroidal thickness (µm) [Median (range)]	202 (84 - 377)	–
RNFL (µm) [Mean (SD)]	93.8 (32.3)	95.8 (11.1)
RNFL (µm) [Median (range)]	94 (33 – 208)	97 (64 – 118)
CME [Number of eyes (%)]	9 (13%)	0 (0%)
Angiographic findings		
FA – vessels [Number of eyes (%)] FA – macula [Number of eyes (%)] FA – optic disc [Number of eyes (%)]	32 (44%) 17 (24%) 13 (18%)	- - -
ICGA Dark dots [Number of eyes (%)]	21 (29%)	–
FAF Hypofluorescent dots [Number of eyes (%)]	22 (31%)	–

OCT, Optical coherence tomography; IMT, Immunosuppressive treatment; AZA, Azathioprin; MTX, Methotrexate; CsA, Ciclosporin A; .

MMF, Mycophenolate mofetil; FAF, Fundus Autofluorescence, RNFL, Retinal nerve fiber layer; FA, Fluorescein angiography; .

ICGA, Indocyanine green angiography; CRT, Central retinal thickness; CME, Cystoid macula edema; .

csDMARD, conventional synthetic disease-modifying antirheumatic drugs.

^ns1^ : Wilcoxon(p=0.885) ^ns2^: Fisher’s exact test (OR=0.43, 95% CI 0.14-1.28, p=0.138).

BU patients younger than 60 years had shorter disease duration and exhibited activity predominantly in both retina (deep or surface) and choroid (surfaceReti+Chor; n = 5 or deepReti+Chor, n = 3), or just in the choroid (Chor, n = 2). This was indicated either by both FA and ICGA findings and the highest median VH or by dark dots on intermediate-phase ICGA and sparse FA leakage. Isolated retina activity (surfaceReti, n = 1 and deepReti, n = 6) was observed more often in older donors and was characterized by vascular leakage in the macular region and partially in the optic disc on FA imaging, without the presence of dark dots on ICGA. Inactive disease (n = 12), characterized by absence of vascular leakage in FA and ICGA imaging, was mainly seen in patients 55–70 years of age with a disease duration of 5–15 years. In contrast, the Burned-out patients represented a structurally advanced disease phenotype rather than a current inflammatory activity category. Burned-out patients with severe visual impairment were the oldest (range 49–77 years) and had the longest disease duration (range 8–28 years). Morphologically, this group was characterized by disruption of the integrity of the ELM and the inner segment/outer segment (IS/OS zone), and the lowest foveal and peripapillary retinal thickness on OCT. Impaired visual acuity was present at the highest rates in patients with activity in deep Retina and Choroid (deepReti+Chor, n = 2/3), followed by deepReti (n = 2/6), Burned-out (n = 3/7), and Inactive patients (n = 1/11) (**Figure 2E**).

No included BU patients were treatment-naïve, and at recruitment each patient had individualized treatment plans (**Figures 2F, G**). Most patients were treated with conventional synthetic disease-modifying antirheumatic drugs (n=12, csDMARDs; azathioprine, mycophenolate mofetil, or methotrexate) and were mainly Inactive (n = 8/12), or were treated with TNF-a antagonists (n = 12, infliximab or adalimumab) and exhibited mainly Retina activity (n = 8/12). Choroid active (surfReti+Chor, Chor). Four Active patients received the IL-6R antagonist tocilizumab. Low-dose prednisolone (<10 mg/day) and cyclosporin A (CsA) were each administered to only one patient. The Burned-out group had the highest number of patients without current treatment.

### Distinct immune signatures in BU

Next, we analyzed the peripheral immunological profile by mass cytometry and related the identified cellular differences to the clinical activity groups (**Figure 3A**). In BU patients, frequencies of Th17 (CCR6^+^ CXCR3^-^ CCR4^+^ CCR10^-^ memory CD4^+^) T_CM_ subsets were significantly elevated independently of disease activity (**Figure 3B**, **Supplementary Figure 6**), in line with the autoimmune etiology of BU and previous reports (16). Among Th17, a higher frequency of CD57^bright^ cells was observed in BU patients, specifically during Active disease (**Figure 3C**). CD57 is characteristic of T cells with reduced proliferative capacity and often associated with chronic immune activation (17, 18). Serum IL-18 was significantly upregulated in BU patients, and was highest in Inactive patients (**Figure 3D**). In BU patients, we observed more CD146^+^ cells among overall CD4^+^ memory T cells and Th22 cells (CCR6^+^ CXCR3^-^ CCR4^+^ CCR10^+^ memory CD4^+^), which were more abundant in Inactive patients (**Figures 3E, F**). CD146 (MCAM) is involved in endothelial homing and associated with autoimmune and neuroinflammatory diseases (19–22). In line with our prior study (23), Active (and Burned-out) patients had more Th1 (CCR6^-^ CXCR3^+^ CCR4^-^ memory CD4^+^) and fewer Th2 (CCR6^-^ CXCR3^-^ CCR4^+^ memory CD4^+^) (**Supplementary Figure 6**), resulting in a higher Th1/Th2 ratio in comparison to Inactive patients (**Figure 3G**). Additionally, effector memory CD4^+^ T cells with co-expression of Th1- and Th2-associated chemokine receptors (CCR6^-^ CXCR3^+^ CCR4^+^) were enriched in BU patients, especially in Active disease (**Figure 3H**). Finally, HCs displayed significantly higher frequencies of CD56^bright^ CD16⁻ NK cells (**Figure 3I**), a subset typically associated with regulatory functions. In contrast, CD56^dim^ NK cells were not altered in BU patients in general but significantly reduced in Inactive and increased in Active disease (**Figure 3J**). The altered CD56^dim^/CD56^bright^ NK cell ratio in BU patients is consistent with earlier reports of a potential imbalance in cytotoxic versus regulatory NK cell responses (24, 25).

**Figure 3:**
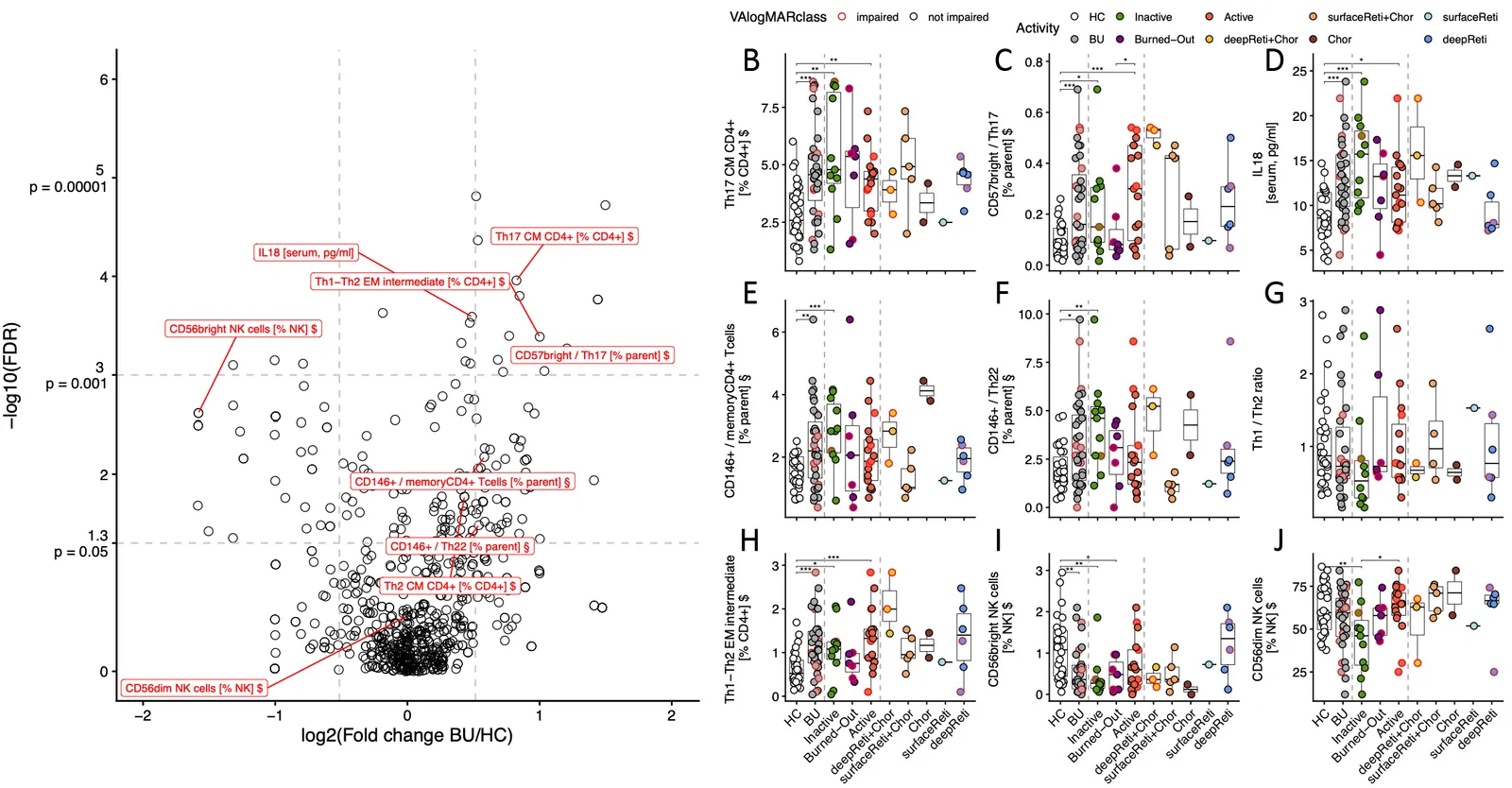
Peripheral immune signatures in birdshot uveitis (BU). C F I D G J **(A)**: Volcano plot of log_2_ fold change (BU vs HC) versus –log_10_(FDR) from CyTOF-derived immune cell subset frequencies. Labeled points denote the most significantly altered populations. Suffix “$” indicates subsets derived by FlowSOM. **(B–J)**: Box plots for representative parameters and subsets with disease activity on the x-axis. Groups are HC and BU, split into Inactive, Burned-Out and Active, which were further split (indicated by dashed lines) according to activity affecting deep retina + choroid (deepReti+Chor), surface retina + choroid (surfaceReti+Chor), choroid (Chor), surface and deep retina (surfaceReti and deepReti); for colors see legend. Horizontal bars and boxes indicate medians and interquartile ranges, respectively. Groups HC, BU, Inactive, Burned-Out and Active were statistically compared using Wilcoxon test. Statistical comparisons were performed using Wilcoxon test (*, p<0.05; **, p<0.01; ***, p<0.001).

### Disease activity-dependent immune signatures

To explore immune signatures associated with clinical disease state, we selected parameters correlating with Activity General and Activity Expert above a significance level of 0.05 and with Eta-Squared above 0.14 (**Supplementary Figures 7A, B**), and clustered the medians per disease-activity group for the most relevant parameters (**Figure 4A**). Given the high dimensionality of the dataset and the modest subgroup sizes, these analyses are considered exploratory and interpreted according to both statistical association and effect size. Accordingly, the Inactive group was characterized by higher levels of stem cell-like memory CD8^+^ T cells (T_SCM_, CD45RA^+^ CCR7^+^ CD95^+^ CD127^+ 26^ (26)) and more TIM-3^+^ cells among Tc17-Tc22 intermediate (CCR6^+^ CXCR3^+^ CCR4^+^) effector memory (EM) CD8^+^ T cells (**Figures 4B, C**). T_SCM_ have been associated with autoimmunity, since they may represent a reservoir of autoimmunity-maintaining T cells, as shown for autoimmune uveitis, rheumatoid arthritis, and systemic lupus erythematosus (SLE) (27–29). Especially the expansion of CD8^+^ T_SCM_ with a reduction of CD4^+^ T_SCM_ seem to be a signature for autoimmunity. TIM-3^+^ Tc17-Tc22 may accumulate in Inactive patients due to chronic autoimmune activation. TIM-3 is an immune checkpoint receptor with reduced expression in several autoimmune diseases, and polymorphisms have been described in inflammatory bowel disease and rheumatoid arthritis (30, 31). Active patients showed increased frequencies of HLA-DR^+^ PD-1^+^ CD25^+^ Th17 and HLA-DR^+^ CD38^+^ Tc1 (CCR6^-^ CXCR3^+^ CCR4^-^ memory CD8^+^) EM (**Figures 4D, E**), while Burned-out patients displayed more PD-1^+^ and CD57^+^ Tc1 EM cells (**Figures 4F, G**), associated with exhaustion and senescence (32–34). These observations point to the *in vivo* activation of autoimmunity-associated T cell subsets during Active disease, whereas Inactive and structurally defined Burned-out patients showed increased expression of immune checkpoint and terminal differentiation markers.

**Figure 4:**
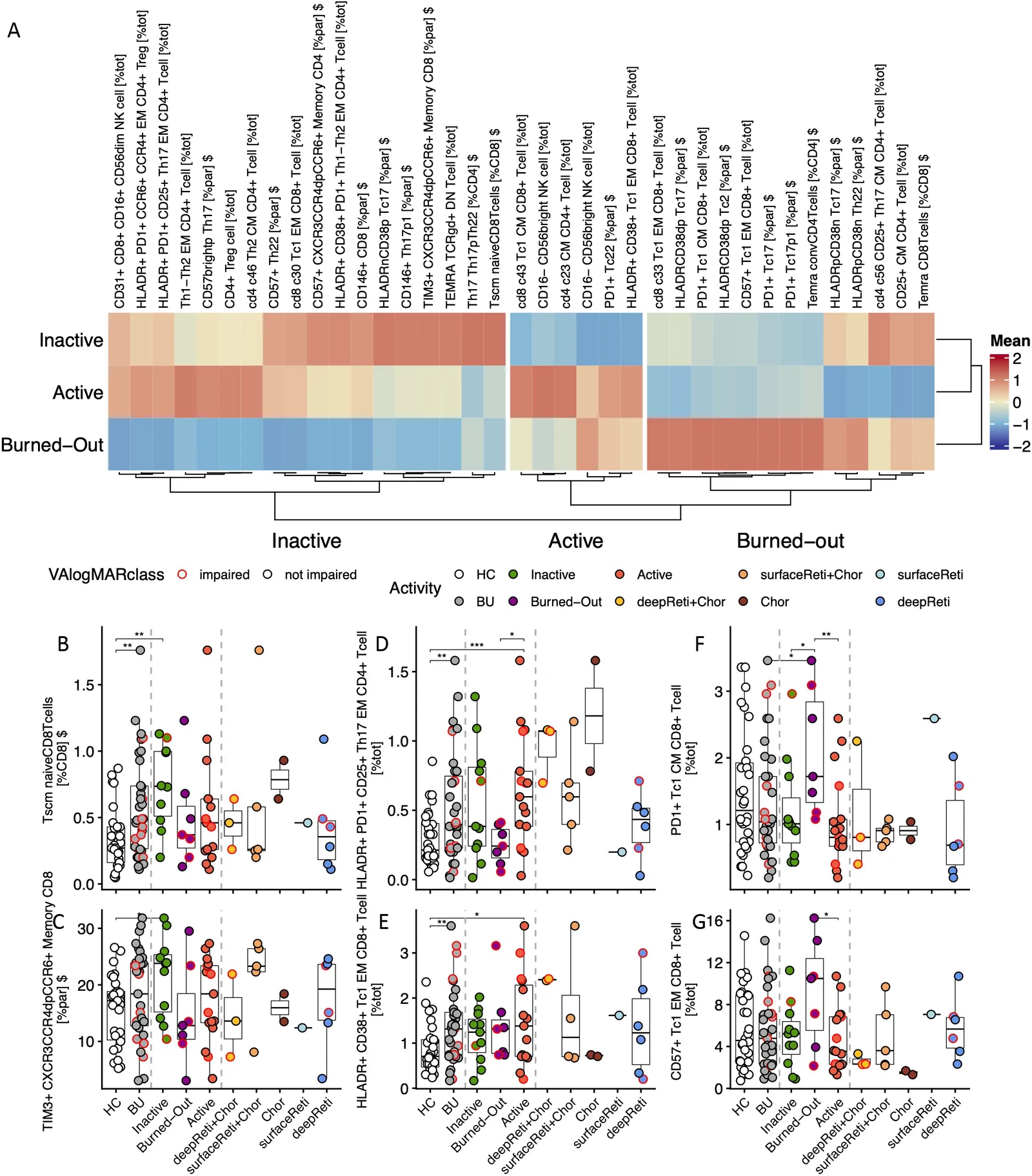
Disease activity–linked immune signatures delineate distinct clusters with shared features across groups. **(A)**: Heatmap of selected CyTOF subsets (rows) across groups (columns) showing median z-scores per group. Features were preselected by effect size (η² > 0.14) from ANOVA across activity states. **(B-G)**: Box plots for representative parameters and subsets with disease activity on the x-axis. Groups are HC and BU, split into Inactive, Burned-Out and Active, which were further split (indicated by dashed lines) according to activity affecting deep retina + choroid (deepReti+Chor), surface retina + choroid (surfaceReti+Chor), choroid (Chor), surface and deep retina (surfaceReti and deepReti); for colors see legend. Horizontal bars and boxes indicate medians and interquartile ranges, respectively. Groups HC, BU, Inactive, Burned-Out and Active were statistically compared using Wilcoxon test. Statistical comparisons were performed using Wilcoxon test (*, p<0.05; **, p<0.01; ***, p<0.001).

### Therapy-linked immune signatures

We next focused on immune profiles associated with current IMT, independent of the clinical activity status (**Supplementary Figure 7B**), and compared the IMT group means of relevant parameters (**Figure 5A**, **Supplementary Figure 7**). Because treatment allocation was clinically driven and strongly associated with disease activity, disease severity, and treatment history, these analyses were considered descriptive and were not interpreted as direct pharmacological treatment effects.

**Figure 5:**
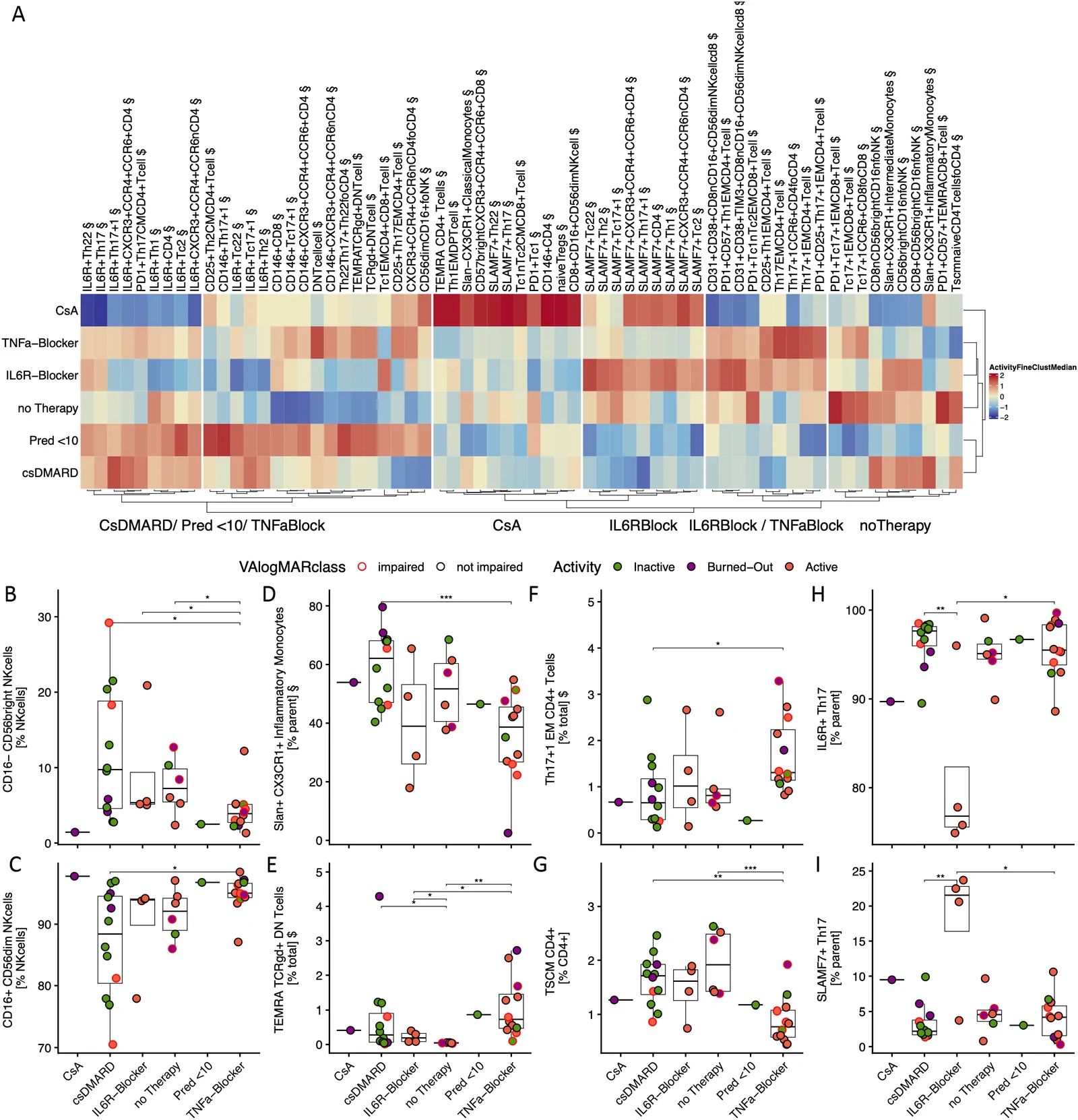
Therapy-linked immune signatures define distinct yet overlapping clusters. **(A)**: Heatmap of subsets (columns) stratified by current immunomodulatory therapy (rows: no therapy, csDMARDs, CsA, TNFα blocker, IL-6R blocker), showing median z-scores. **(B-I)**: Box plots for representative populations across therapy classes illustrate activity stage differences (e.g. SLAMF7^+^ and IL-6R^+^ Tc17+1, CD56^dim/bright^ NK, inflammatory monocytes). Box plots show current IMT on the x-axis and dots are colored according to disease activity. Statistical comparisons were performed using Wilcoxon test (*, p<0.05; **, p<0.01; ***, p<0.001).

Patients receiving csDMARDs and TNF-α antagonists showed contrasting immune profiles across several parameters. For example, CD56^bright^ CD16^–^ NK cells, Slan^+^ inflammatory monocytes, and CD4^+^ T_SCM_ were highest in the csDMARD group and lowest in patients receiving TNF-α antagonists, while CD56^dim^ NK cells, TCRγδ^+^ T_EMRA_, and Th17 + 1 (CCR6^+^ CXCR3^+^ CCR4^-^ memory CD4^+^) EM displayed the opposite trend (**Figures 5A–G**). The CsA subgroup consisted of a single patient and is therefore presented descriptively only. This patient showed elevated frequencies of CD57^bright^ and CD146^+^ CD4^+^ T cell subsets (**Figure 5A**) and a high proportion of CD56^dim^ CD16^+^ NK cells (**Figure 5C**). In the IL-6R antagonist-treated patients, a marked decline in IL-6R^+^ T helper subsets was accompanied by a reciprocal increase in SLAMF7^+^ populations (**Figures 5H, I**). Patients without current IMT had higher frequencies of PD-1^+^ and SLAMF7^+^ Tc17 + 1 cells (CCR6^+^ CXCR3^+^ CCR4^-^ memory CD8^+^), inflammatory monocytes, and PD-1^+^CD57^+^ CD8^+^ T_EMRA_ cells. These differences should be interpreted as treatment-associated patterns rather than treatment-specific effects because treatment groups differed substantially in clinical disease state and sample size.

## Discussion

In this study, we have utilized mass cytometry to immunophenotype and quantify more than 200 subsets of T cells and NK cells in BU patients and HCs. We combined the immunophenotyping data and multiplex serological readouts with disease activity staging based on expert ophthalmologic assessment to identify not only BU-related but also disease activity-associated immune signatures. Importantly, our clinical stratification incorporated two complementary dimensions: current inflammatory activity, defined by multimodal imaging, and cumulative structural disease stage, reflected by morphological damage, disease duration, and visual impairment. This distinction was particularly relevant for the Burned-out phenotype, which was considered an advanced structural disease state rather than an active inflammatory category.

In BU patients, we observed increased serum IL-18 levels, Th17 central memory (CM), CD146^+^ memory CD4^+^ T cells, and reduced CD56^bright^ NK cells. Increased CD146^+^ Th22 subsets and IL-18 levels in the peripheral circulation in BU patients may indicate recruitment to and/or recirculation from inflamed tissue sites. In addition, we observed more CXCR3^+^CCR4^+^ double-positive T cells, representing intermediates between Th1 and Th2. CXCR3 indicates Th1 priming and guides migration along a gradient of C-X-C motif chemokine ligands CXCL9/10/11 (35, 36). The enrichment of CXCR3^+^CCR4^+^ effector memory T (T_EM)_ cells resembles patterns seen in psoriasis, in which this intermediate phenotype marks tissue-homing CD8^+^ T cells associated with disease relapse, connecting our findings to another MHC-I-mediated pathology (37). Together, these findings represent T-cell-centric autoimmune signatures and underscore reports on T cell involvement in BU.

The IMT subgroups showed distinct peripheral immune profiles. However, these patterns should be interpreted descriptively because treatment allocation was clinically driven and confounded by disease activity, disease severity, and treatment history. Patients receiving csDMARDs were predominantly Inactive and showed expansion of CD56^bright^ CD16^-^ NK cells, associated with immunoregulatory properties. CD56^bright^ CD16^-^ NK cells are a small fraction of the NK cell compartment, but one case study of five uveitis patients linked their expansion to treatment efficacy of daclizumab, an IL-2R antagonist (38). NK cell-mediated immunoregulation of activated T cells during daclizumab therapy has previously been shown in multiple sclerosis patients, with expansion of CD56^bright^ NK cells correlating with treatment response (39). By comparison, in patients treated with TNF-α antagonists, CD56^bright^ NK cells remained comparatively low, while inflammatory monocytes declined markedly. Monocytes are a major source of TNF-α, and antagonizing TNF-α may effectively reduce inflammatory monocyte differentiation (40). Tocilizumab was effective in treating refractory BU. In our recently published longitudinal cohort and other studies, tocilizumab treatment was associated with sustained reduction of retinal vasculitis and CRT, whereas choroidal inflammatory activity showed a less consistent response over time (41–43). One patient from our study achieved a sustained reduction of clinical activity later during treatment (data not shown). In the present cohort, patients receiving tocilizumab showed markedly reduced detectable IL-6R^+^ subsets, accompanied by an increase in SLAMF7^+^ cells. While downregulation and internalization of the IL-6R with treatment can be expected, the pronounced reduction in detectable IL-6R^+^ subsets may additionally reflect epitope masking by tocilizumab rather than true biological downregulation. Apparently, tocilizumab can interfere with the staining antibody, which highlights the technical difficulties in monitoring treatment with biologics. Given the observational design and confounding by indication, these findings are interpreted as therapy-associated immune patterns rather than direct pharmacological effects.

We applied a disease activity staging approach that allows us to describe a cross-sectional pattern compatible with the following sequence of events in BU (summarized in **Figure 6**). Patients with shorter disease duration (<5 years after diagnosis) more frequently showed choroidal and combined Retina-Choroid activity, whereas isolated Retina activity was more often observed at longer disease duration (5–10 years after diagnosis). Because these observations are cross-sectional, they should not be interpreted as direct longitudinal evidence of a fixed temporal sequence. Active episodes alternate with inactive phases in a relapse-remission cycle typical of autoimmunity. This sequence can be explained by different immune-privileged states of choroid and retina, conveyed by the two BRBs. The outer BRB, encompassing choriocapillaris, RPE, and Bruch’s membrane, functions as the more immunomodulatory and dynamic epithelial barrier, whereas the inner BRB is more tightly regulated and less permeable, comparable to the blood–brain barrier (44, 45). In late stages (>10 years), patients predominantly exhibit an end-stage, Burned-out phenotype with simultaneous involvement of retina and choroid, dense T cell infiltrates, and structural loss of photoreceptor/RPE layers, underscoring chronic damage due to compartment-spanning inflammation (46). In our classification, Burned-out therefore represents cumulative structural disease rather than a current inflammatory activity state. The Burned-out disease stage is traditionally considered inactive, but our peripheral immune data indicate that these patients are not necessarily immunologically quiescent. This does not establish ongoing intraocular inflammation or a requirement for continued immunosuppression, but suggests that the absence of angiographic activity does not necessarily coincide with normalization of the peripheral immune profiles.

**Figure 6:**
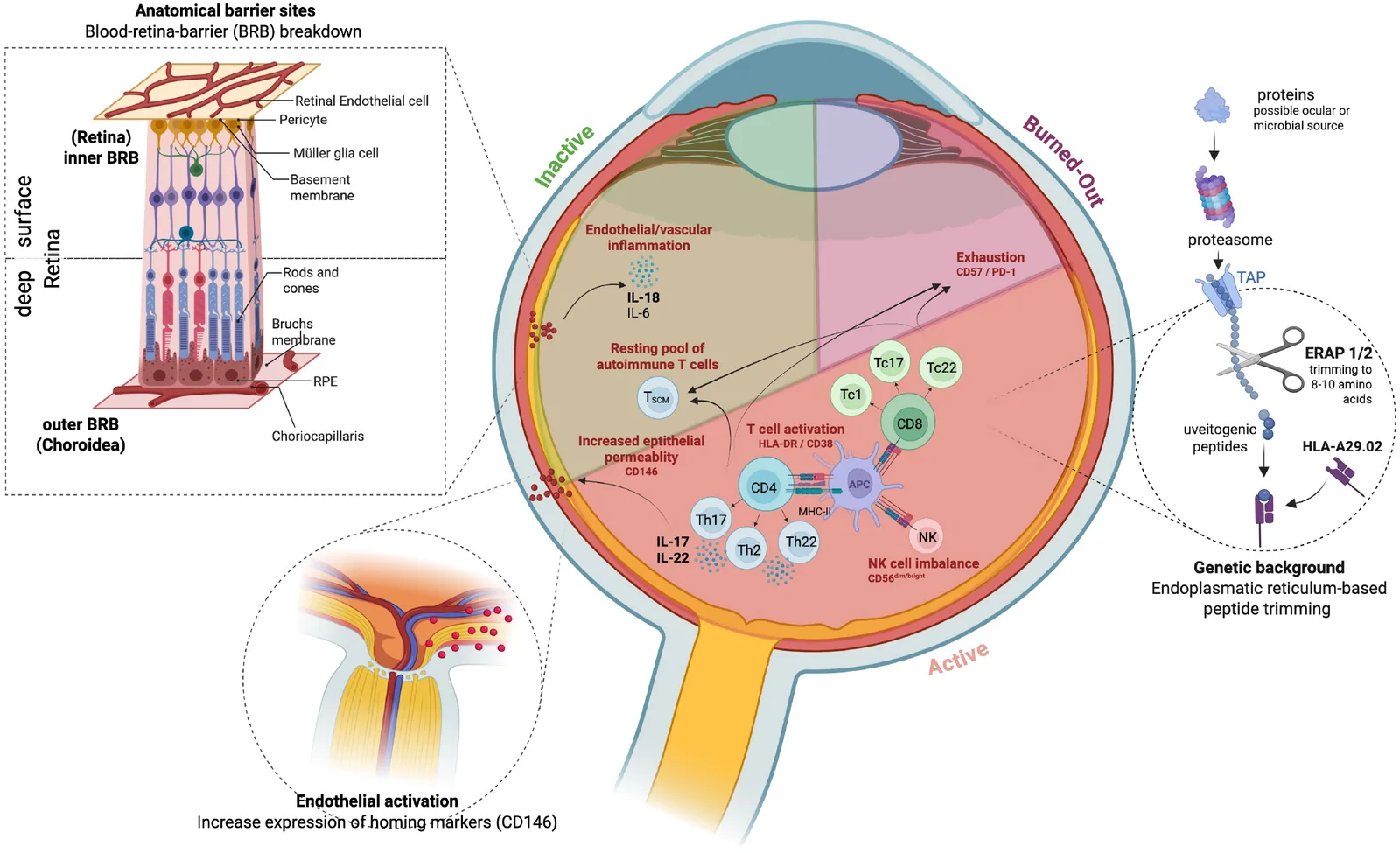
Schematic summary The HLA-A*29 allele and variants of the aminopeptidases ERAP1 and ERAP2 have been identified by prior genome-wide association studies as main risk factors for BU, influencing the antigenic landscape. Our peripheral blood profiling highlights the T and NK cell activation states that may be associated with immune-cell trafficking across the inner and outer blood-retinal barriers and contribute to the different disease activity phenotypes among patients. Created with BioRender.

Inactive phases were immunologically characterized by increased CD8^+^ T_SCM,_ serum IL-18, and a skew towards Th22 and Th/c17, suggesting endothelial involvement. Notably, IL-18 was the only serum cytokine showing significant differences between HCs and BU patients, while the remaining cytokines/chemokines were either not detected at all or did not show clear and significant differences between BU and HCs or among disease activity groups (**Supplementary Figure 8**). IL-18, often produced by epithelial cells, can increase IL-17 and IL-22 production in Th17/Th22 and CD146 expression (47, 48). IL-22, in turn, can increase endothelial permeability and proliferation, potentially sustaining leakage and vascular remodeling. Active disease was characterized by elevated Th17, Tc1, and CD146^+^ Th22, and a pronounced upregulation of exhaustion markers like PD-1 and CD57, e.g., on HLA-DR^+^ PD-1^+^ Th17 T_EM_ subsets. The PD-1 phenotype aligns with chronic antigen exposure and exhaustion-associated differentiation states in tissue-engaged T cells (32). Concomitant CD57^bright^ expression marks terminal differentiation or senescence with reduced proliferative capacity but preserved cytotoxic potential, typical of long-standing inflammation (49). Mature CD56^dim^ NK cells, increased in Active patients, may interact with inflamed epithelial cells and the IL-15/IL-18 microenvironment (50). Burned-out patients exhibited T cells with clear signs of exhaustion, including PD-1 and CD57 expression. Thus, although Burned-out patients were morphologically classified as end-stage and showed limited overt angiographic inflammation, they retained a distinct peripheral immune phenotype characterized by markers associated with chronic antigen experience and exhaustion. In our pilot study, we observed increased CD8^+^ T_EMRA_ cells in Inactive patients (23). T_EMRA_ subsets accumulate with age, which contributes to the findings in the older Burned-out patients but does not fully account for the increased frequencies when compared to age-matched HCs. While TCRγδ expression was not examined in the pilot study, both findings indicate that increased T_EMRA_ frequencies are indicative of advanced disease (23). These findings further support separating structural disease stage from current inflammatory activity when interpreting immune profiles.

In this study, we utilized mass cytometry on a clinically well-characterized BU cohort. The observed immune signatures support a working model in which choroidal and retinal inflammatory phenotypes are associated with different peripheral immune profiles, and advanced structural disease is accompanied by persistent immune alterations. Our cross-sectional data are compatible with earlier choroidal/combined involvement and later predominant retinal involvement, but do not establish a fixed temporal progression. That peripheral immune readouts change with disease activity explains why cross-sectional studies have been inconsistent and underscores the value of our disease activity-aware design. Equally, the persistence of altered immune signatures in structurally advanced Burned-out disease illustrates why inflammatory activity and cumulative damage should be considered as separate but complementary clinical dimensions. In future trials, stratification of BU patients by Retina vs. Choroid Activity may increase power and interpretability. A compact flow cytometry panel enabling assessment of functionally different Th/Tc subsets and CXCR3^+^CCR4^+^ intermediates, their *in vivo* activation (HLA-DR, CD38) or exhaustion-associated and terminal differentiation markers (PD-1/CD57/TIM-3), and the CD56^dim/bright^ NK cell ratio as candidate monitoring parameters may complement multimodal imaging to characterize disease activity and detect subclinical shifts.

Our analysis did not include treatment-naïve patients, thus limiting conclusions regarding BU pathogenesis versus therapeutic effects. Since treatment allocation was not randomized but clinically driven, resulting in substantial confounding by indication and collinearity between treatment, disease activity, and disease severity, the treatment-associated immune profiles presented here should be regarded as descriptive and hypothesis-generating rather than as evidence of treatment-specific effects. Furthermore, many immune changes that occur with age, such as T_EMRA_ expansion and NK cell differentiation, are associated with latent viral infections and their reactivation (51). Since we have not assessed latent viruses, e.g., cytomegalovirus (CMV) seropositivity, we cannot rule out that some observations may be associated with age-dependent virus reactivation rather than disease activity. Although age, sex, disease duration, and treatment status are potential confounders, full multivariable or mixed-effects adjustment for all four variables is not reliably feasible with the available sample size. Exploratory linear models adjusting for age and sex yielded only minor changes in the reported estimates. Inclusion of disease duration, however, attenuated many differences, largely because this variable is strongly associated with disease activity groups and is zero among healthy controls by definition. Residual confounding therefore remains a limitation of the present analyses. Further limitations include those inherent in peripheral sampling (e.g., intraocular events are inferred), modest sample sizes for certain disease activity and therapy subgroups, the lack of HLA typing of HCs to investigate potential HLA-A*29-associated effects, and a T/NK cell-focused panel that did not comprehensively profile myeloid or B cell subsets. Integration of antigen specificity and T cell receptor clonality will be an important next step in understanding BU immunopathology.

## Data Availability

The raw data supporting the conclusions of this article will be made available by the authors, without undue reservation.
