# Supplementary material for: Peripheral immune profiles separate disease activity stages in birdshot uveitis
Source: Front Immunol. 2026 Oct 2;17:1938305. doi: 10.3389/fimmu.2026.1938305 (PMC13672581; doi:10.3389/fimmu.2026.1938305)
Supplement: Supplementary file 1 [file DataSheet1.pdf]

Supplementary Table 1
Antibody Panel

Isotope	Antigen	Manufacturer	CatalogID	Clone
89Y	CD45	Biolegend	304045	HI30
141Pr	CD1c	Biolegend	331502	L161
142Nd	CD57	Biolegend	322325	HCD57
143Nd	TCRgd	Biolegend	331202	B1
144Nd	CD15	Biolegend	323035	W6D3
145Nd	CD146	Biolegend	361002	P1H12
146Nd	CCR4 / CD194	Biolegend	359402	L291H4
147Sm	Slan	Miltenyi	130-095-212	DD-1
148Nd	CD69	Biolegend	310939	FN50
149Sm	CD127	Biolegend	351337	A019D5
150Nd	CD27	Biolegend	302839	O323
151Eu	CD123	Biolegend	306027	6H6
152Sm	CD45RA	Biolegend	304143	HI100
153Eu	CX3CR1	Biolegend	341602	2A9-1
154Sm	TIM-3	Biolegend	345019	F38-2E2
155Gd	CD14	Biolegend	301843	M5E2
156Gd	CD183_CXCR3	Biolegend	353733	G025H7
158Gd	CCR10	Biolegend	341502	6588-5
159Tb	PD-1	Biolegend	329941	EH12.2H7
160Gd	CD28	Biolegend	302937	CD28.2
161Dy	CD8	In-house		GN11137
162Dy	IgD	Biolegend	348235	IA6-2
163Dy	CD56	Biolegend	318345	HCD56
164Dy	CD95	Biolegend	305631	DX2
165Ho	CCR6 / CD196	Biolegend	353427	G034E3
166Er	CD19	Biolegend	302247	HIB19
167Er	CCR7	Biolegend	353237	G043H7
168Er	CD4	In-house		TT1
169Tm	CD25	Biolegend	356102	M-A251
170Er	CD3	BD	555330	UCHT1
171Yb	SLAMF7 / CD319	Biolegend	331802	162.1
172Yb	CD38	Biolegend	303535	HIT2
173Yb	CD141	Biolegend	344102	M80
174Yb	CD31	Biolegend	303127	WM59
175Lu	CD126_IL6R	Biolegend	352802	UV4
176Yb	HLA-DR	Biolegend	307651	L243
209Bi	CD16	Biolegend	302051	3G8

Supplementary Figure 1

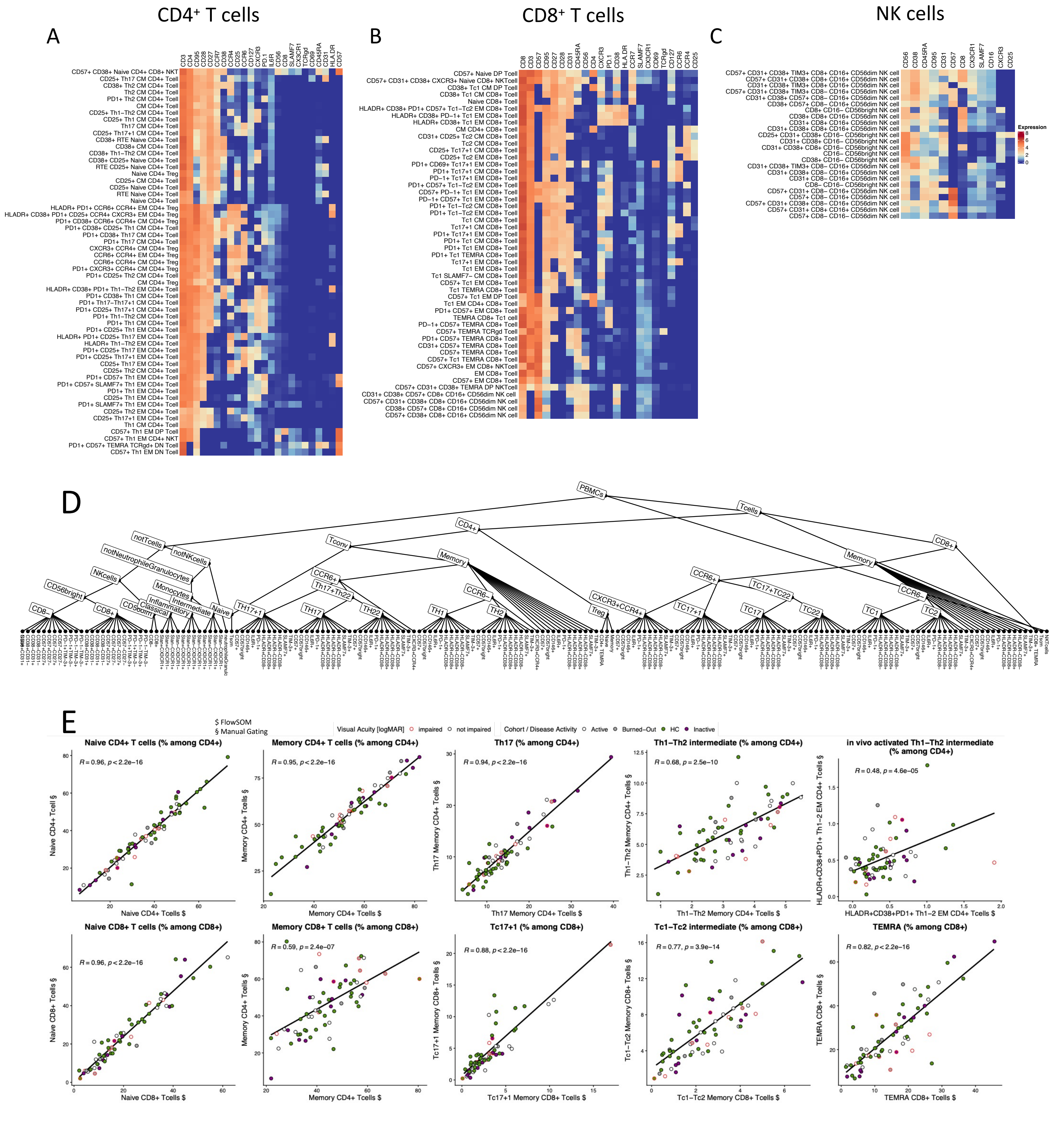

Supplementary Figure 1. Masscytometry immunoprofiling of T and NK cells using FlowSOM and manual gating confirming comparable results and focusing on rare T cell populations.

A-C: CD8⁺ and CD4⁺ T cells as well as NK cells were clustered using FlowSOM (240 cell clusters), followed by manual cluster annotation and UMAP embedding. **D:** Hierarchical scheme of the T and NK cell-focused gating strategy. **E:** Correlation of FlowSOM (indicated as "$")- and manual gating(§)-derived cell frequencies of matching populations revealed high comparability of results. Pearson correlation coefficients R and p-values are displayed.

Supplementary Figure 2

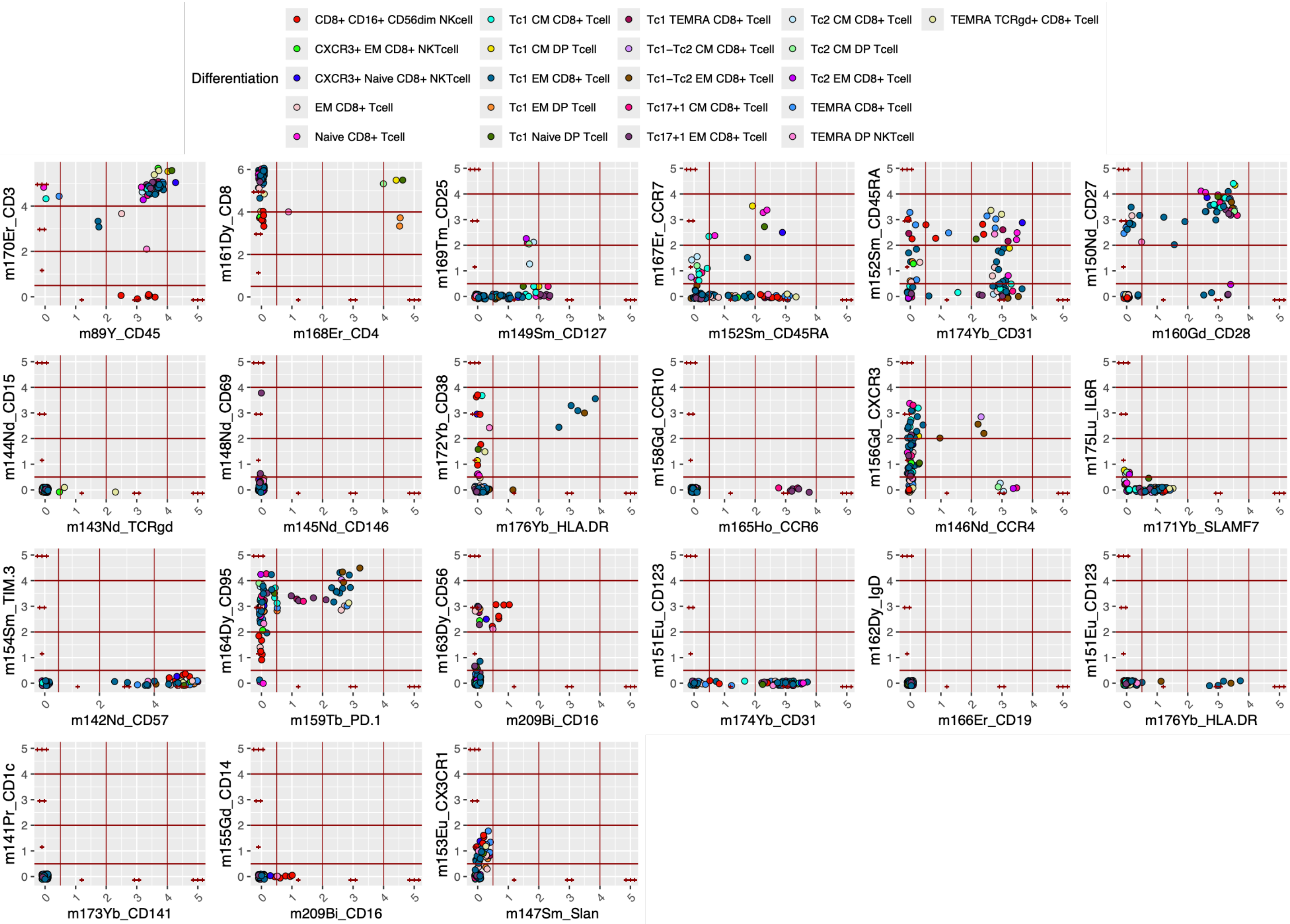

Supplementary Figure 2: Phenotype annotation of FlowSOM-derived clusters.

FlowSOM-derived clusters were assigned with phenotype annotations using means of marker expression. Here, exemplary differentiation phenotypes (such as Tc1, Tc17 etc.) among CD8 cells is shown. See Appendix for all phenotypes.

Supplementary Figure 3

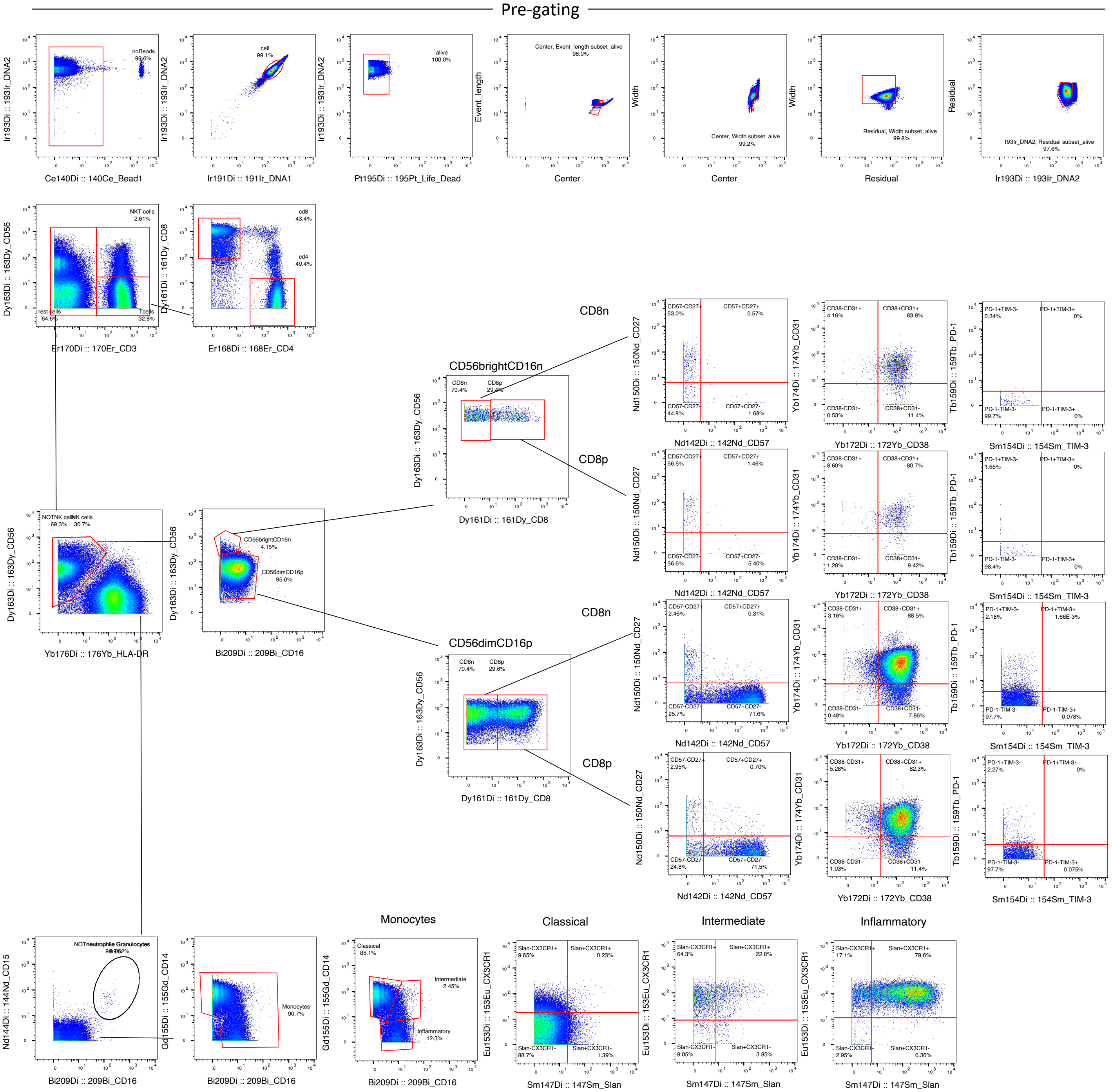

Supplementary Figure 3: Manual gating scheme including pre-gating and NK cells and Monocytes.

Total events are pre-gated by exluding normalization beads, gating on living cells (DNA+ and Pt(Platin)-) and then on single cells (via Event length / Center / Width / Residual). Living singl cells are separated via CD3 and CD56 into NK cells, T cells and NK T cells. T cells are separated into CD4 and CD8 (separate gating schemes in Supp. Fig. 4 and 5). HLA-DR+ cells are excluded from NK cells, which are further split into CD56dim and CD56bright and those sepatated into CD8- and CD8+. NK cell subsets were further characterized for CD57 vs. CD27, CD38 vs. CD31 and TIM-3 vs. PD-1 expression.

From the HLA-DR+ cells excluded from NK cell gate were further cleaned from CD15+ CD16+ neutrophil Granulocytes to then assess Monocytes, which are split into Classical (CD14++CD16-), Intermediate (CD14+CD16+) and Inflammatory Monocytes (CD14- CD16+), and further assessed for Slan vs. CX3CR1.

Supplementary Figure 4

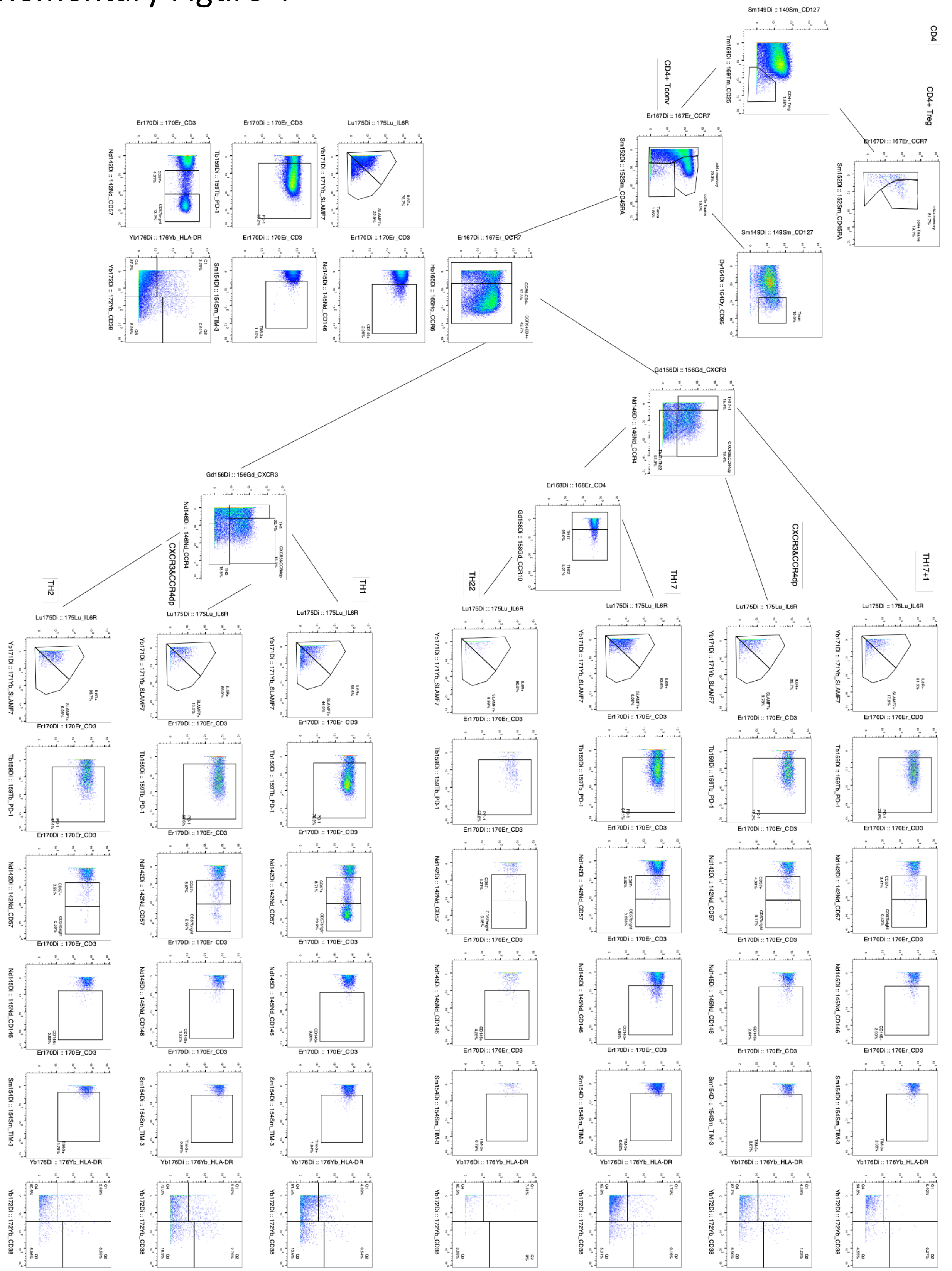

Supplementary Figure 4: Manual gating scheme of CD4+ T cells.

Pre-gated CD4+ T cells (see Supp. Fig. 3) were first split into conventional T cells (Tconv) and CD25^{bright} CD127^{low} regulatory T cells (Tregs). Tconv were further split into memory T cell subsets: naïve (T_N, CCR7$^+$CD45RA$^+$), memory (CCR7$^{+/-}$CD45RA$^-$), and terminally differentiated effector memory T cell re-expressing CD45RA (T_{EMRA}, CCR7$^-$CD45RA$^+$). In naïve, stem cell-like memory T cells (T_{SCM}) were identified as CD95$^+$ CD127$^+$. Memory T cells were further divided based on chemokine receptor expression (CCR6, CXCR3, CCR4, and CCR10) into helper and cytotoxic T cell (Th/Tc) subsets: Th1/Tc1, Th2/Tc2, Th17+1/Tc17+1, Th17/Tc17, and Th22/Tc22. These subsets were further characterized regarding PD-1, CD57, TIM-3, HLA-DR and CD38 expression.

Supplementary Figure 5

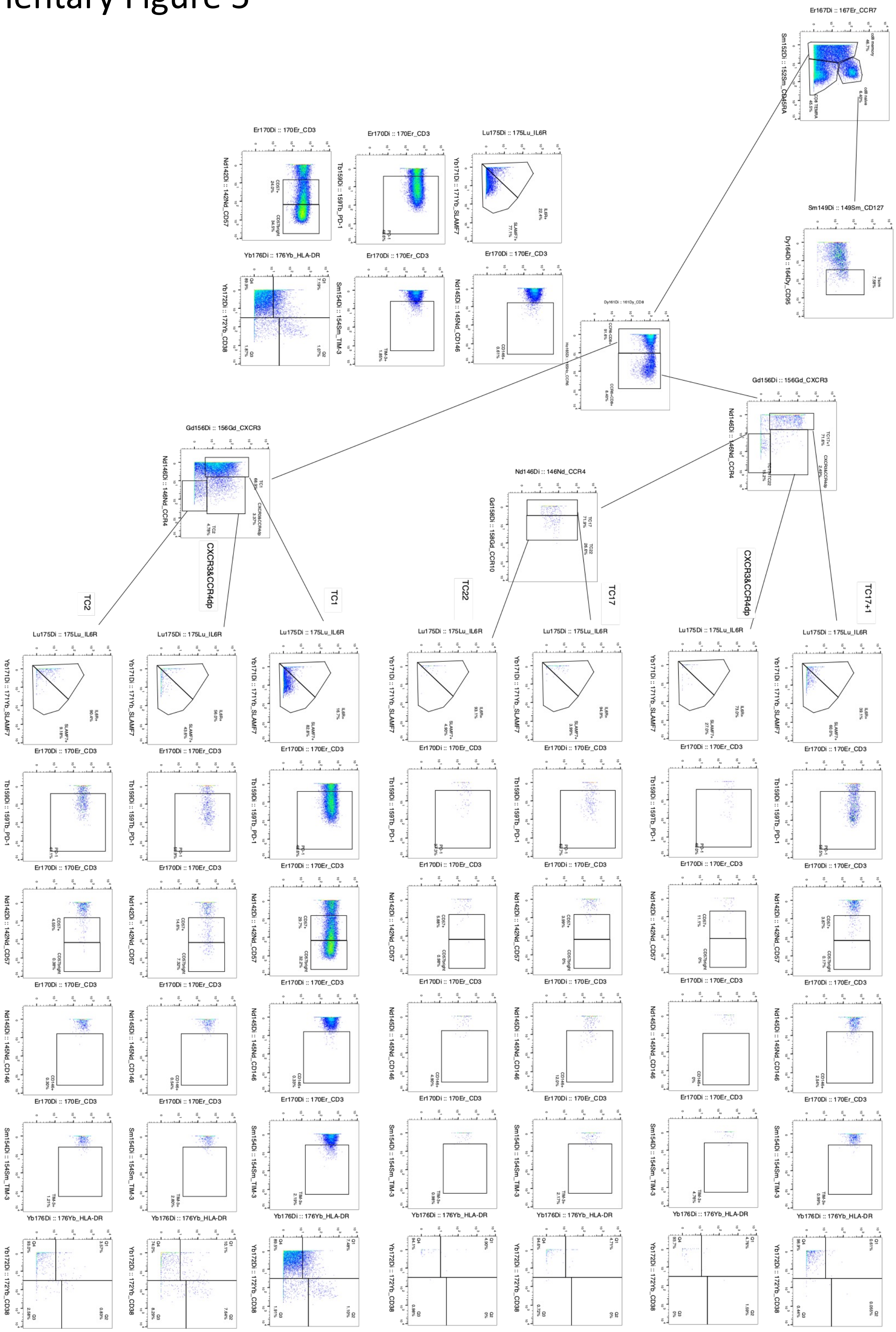

Supplementary Figure 5: Manual gating scheme of CD8+ T cells.

Pre-gated CD8+ T cells (see Supp. Fig. 3) were split into memory T cell subsets: naïve (T_N, $CCR7^+CD45RA^+$), memory ($CCR7^{+/-}CD45RA^-$), and terminally differentiated effector memory T cell re-expressing CD45RA (T_{EMRA}, $CCR7^-CD45RA^+$). In naïve, stem cell-like memory T cells (T_{SCM}) were identified as $CD95^+$ $CD127^+$. Memory T cells were further divided based on chemokine receptor expression (CCR6, CXCR3, CCR4, and CCR10) into helper and cytotoxic T cell (Th/Tc) subsets: Th1/Tc1, Th2/Tc2, Th17+1/Tc17+1, Th17/Tc17, and Th22/Tc22. These subsets were further characterized regarding PD-1, CD57, CD146, TIM-3, HLA-DR and CD38 expression.

Supplementary Figure 6

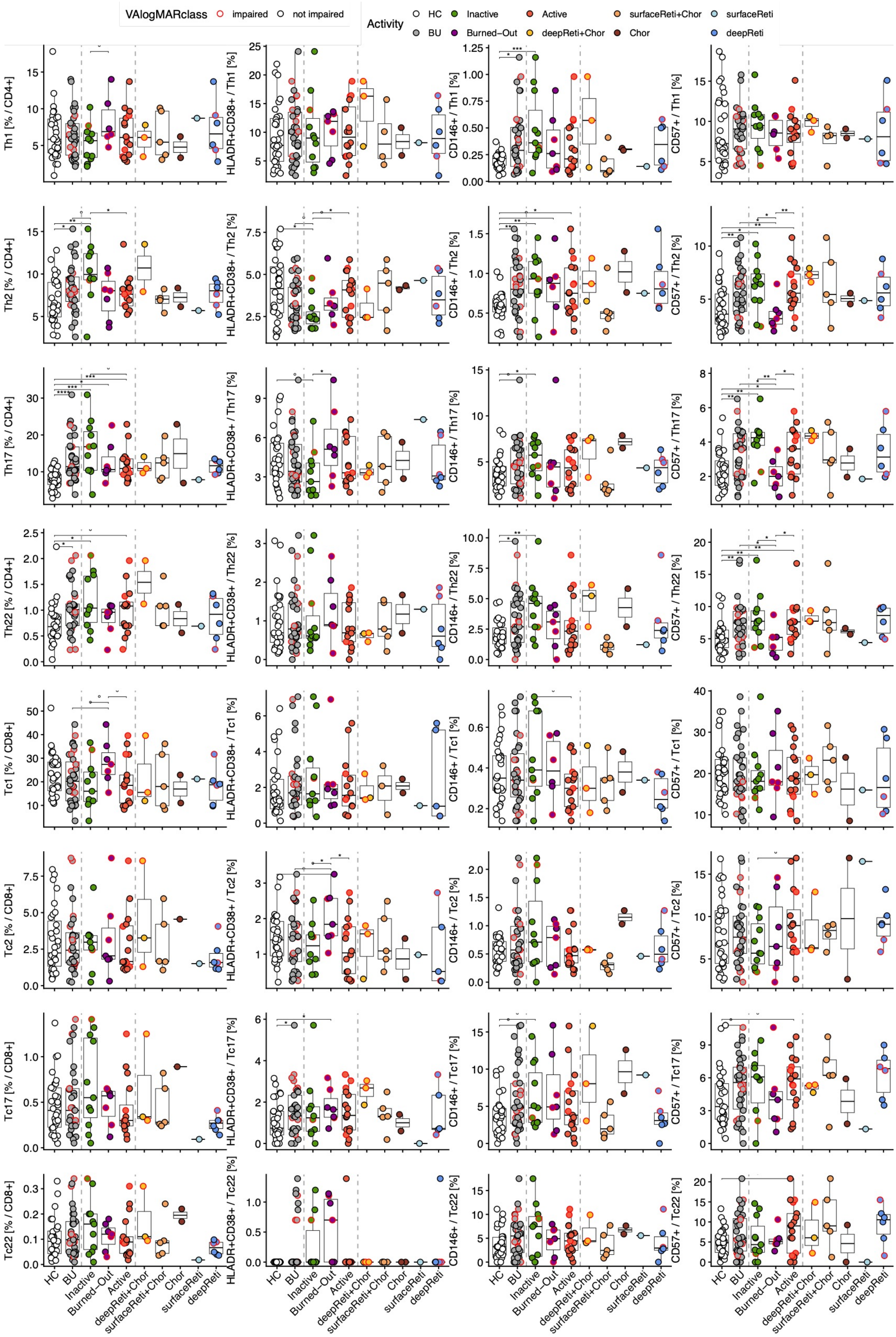

Supplementary Figure 6: Overview about TH and TC subsets.

B-J: Box plots for representative parameters and subsets with disease activity on the x-axis. Groups are HC and BU, split into Inactive, Burned-Out and Active, which were further split (indicated by dashed lines) according to activity affecting deep retina + choroid (deepReti+Chor), surface retina + choroid (surfaceReti+Chor), choroid (Chor), surface and deep retina (surfaceReti and deepReti); for colors see legend. Horizontal bars and boxes indicate medians and interquartile ranges, respectively. Groups HC, BU, Inactive, Burned-Out and Active were statistically compared using Wilcoxon test (*, p<0.05; **, p<0.01; ***, p<0.001

Supplementary Figure 7

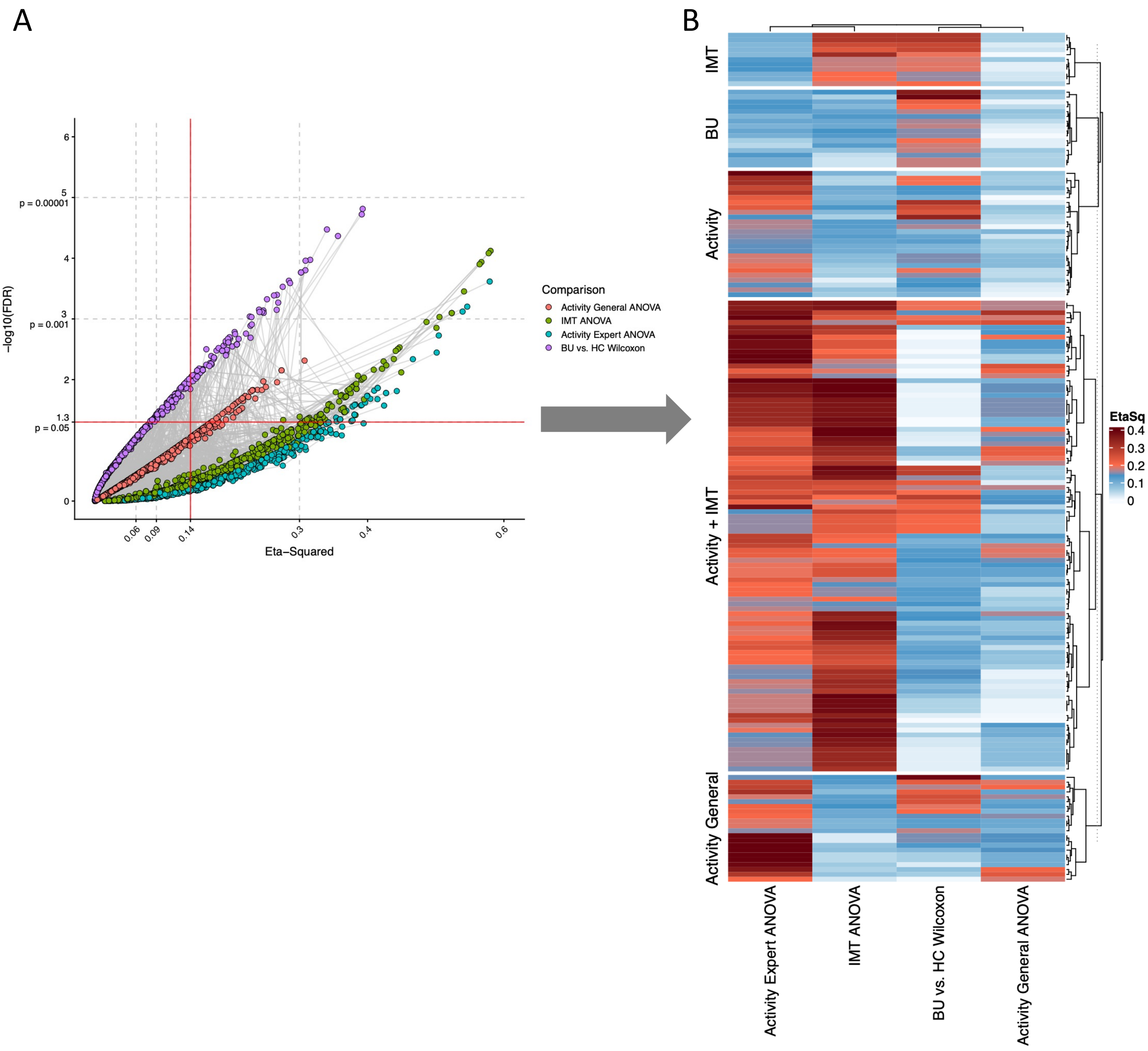

Supp. Figure 7: Effect sizes of several comparisons allow identifification of parameters associated with BU alone, Activity, IMT or Activity and IMT.

A: Volcano plot of Eta-Squared versus negative log_{10}(FDR) from Wilcoxon test for Cohort or Anova over different disease activity strata or treatment regiments (comparison indivated by color). **B:** Heatmap showing Eta-Squared across different comparisons of parameters filtered for significance in at least one comparison (p <= 0.05). Colors indicate Eta-Squared of indicated statistical test. Parameters with effect sizes greater 0.14 were grouped as indicated by the row group titles and further investigated separately in Figure 4 and 5.

Supplementary Figure 8

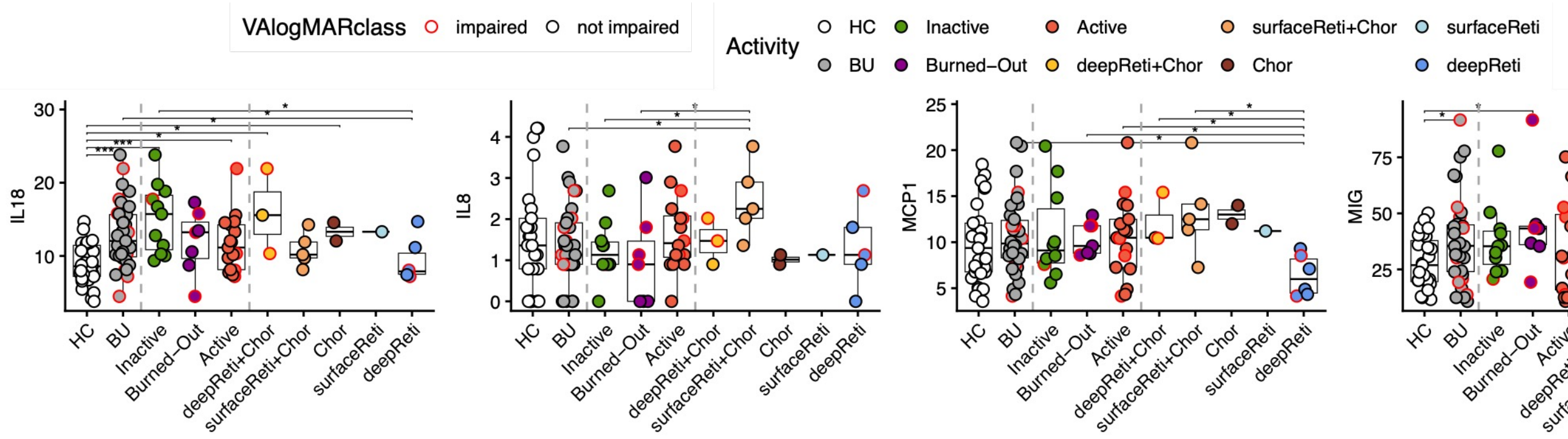

Supp. Figure 8: Overview over altered serum cytokines.

Box plots for the cytokines with any significant difference (IL-18, IL-8, MCP-1/CCL2 and MIG/CXCL9) with disease activity on the x-axis. Groups are HC and BU, split into Inactive, Burned-Out and Active, which were further split (indicated by dashed lines) according to activity affecting deep retina + choroid (deepReti+Chor), surface retina + choroid (surfaceReti+Chor), choroid (Chor), surface and deep retina (surfaceReti and deepReti); for colors see legend. Horizontal bars and boxes indicate medians and interquartile ranges, respectively. Groups HC, BU, Inactive, Burned-Out and Active were statistically compared using Wilcoxon test (*, $p<0.05$; **, $p<0.01$; ***, $p<0.001$).
