# Supplementary material for: Peripheral immune profiles separate disease activity stages in birdshot uveitis
Source: Front Immunol. 2026 Oct 2;17:1938305. doi: 10.3389/fimmu.2026.1938305 (PMC13672581; doi:10.3389/fimmu.2026.1938305)
Supplement: Supplementary file 2 [file DataSheet2.pdf]

APPENDIX

Pohlmann-Krappitz et al.

Peripheral immune profiles separate disease activity stages in Birdshot Uveitis

page		
1	**FlowSOM cluster phenotyping**	Staining intensity (x-axis) of all displayed markers (y-axis) for all clusters. Vertical cutoff lines used for phenotyping.
2-4	**FlowSOM cluster annotation of CD4, CD8 and NK**	Phenotypes in Column C are aggregated into groups at 4 levels: Cell population, Memory subset, Differentiation subsets. All compiled and additonal markers in Full label.
5	**Overview Clusters per panel**	FlowSOM clusters displayed in xy-plots as overview
6-8	**Overview Cell population for CD4, CD8 and NK**	FlowSOM clusters aggregated into cell populations (level 1) displayed in xy-plots for all relevant markers for CD4, CD8 and K on separate pages
9-11	**Overview Memory subsets for CD4, CD8 and NK**	FlowSOM clusters aggregated into memory subsets (level 2) displayed in xy-plots for all relevant markers for CD4, CD8 and K on separate pages
12-14	**Overview Differentiation for CD4, CD8 and NK**	FlowSOM clusters aggregated into Differentiation subsets (level 3) displayed in xy-plots for all relevant markers for CD4, CD8 and K on separate pages
15-17	**Overview Full label for CD4, CD8 and NK**	FlowSOM clusters aggregated into final, full label (level 4) displayed in xy-plots for all relevant markers for CD4, CD8 and K on separate pages

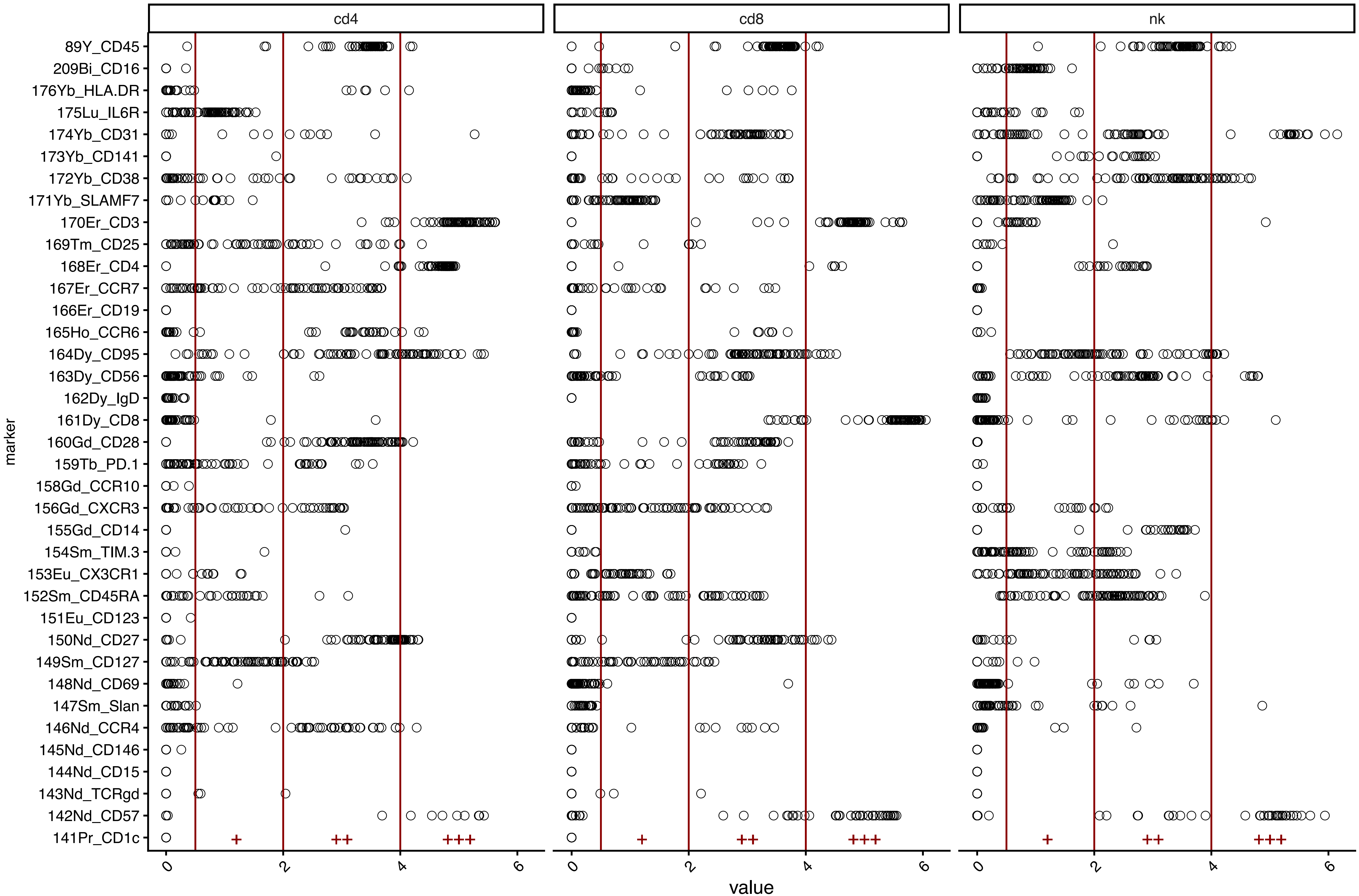
cd4
cd8
nk
89Y_CD45
209Bi_CD16
176Yb_HLA.DR
175Lu_IL6R
174Yb_CD31
173Yb_CD141
172Yb_CD38
171Yb_SLAMF7
170Er_CD3
169Tm_CD25
168Er_CD4
167Er_CCR7
166Er_CD19
165Ho_CCR6
164Dy_CD95
163Dy_CD56
162Dy_IgD
161Dy_CD8
160Gd_CD28
159Tb_PD.1
158Gd_CCR10
156Gd_CXCR3
155Gd_CD14
154Sm_TIM.3
153Eu_CX3CR1
152Sm_CD45RA
151Eu_CD123
150Nd_CD27
149Sm_CD127
148Nd_CD69
147Sm_Slan
146Nd_CCR4
145Nd_CD146
144Nd_CD15
143Nd_TCRgd
142Nd_CD57
141Pr_CD1c
marker
+
++
+++
0
2
4
6
value

	A	B	C	D	E	F	G
1	**cluster**	**panel**	phenotype	**Full label**	**Differentiation**	**Memory Subset**	**Cell population**
2	**c01**	**cd4**	CD45pppCD3pppCD4pppCD14ppCD127pCD45RApCCR7ppCD31pppCD28ppIL6RpHLA.DRppCD38ppTIM.3pCD95pppSlanpCD141pCD27pp	**Trash? Monocyte**	**Trash? Monocyte**	**Trash? Monocyte**	**Trash? Monocyte**
3	**c02**	**cd4**	CD45pppCD3pppCD56ppCD4pppCD8pCD127pCD45RAppCCR7ppCD31pCD28ppIL6RpSLAMF7pCD38ppCD57pppCX3CR1pCD95ppCD27pp	**CD57+ CD38+ Naive CD4+ CD8+ NKT**	**Naive CD4+ CD8+ NKT**	**Naive CD4+ CD8+ NKT**	**CD4+ CD8+ NKT**
4	**c03**	**cd4**	CD45ppCD3pppCD4pppCD127pCD45RApCCR7ppCD31ppCD28ppIL6RpCD38ppCD95pCD27ppp	**CD38+ RTE Naive CD4+ Tcell**	**RTE Naive CD4+ Tcell**	**Naive CD4+ Tcell**	**CD4+ Tcell**
5	**c04**	**cd4**	CD45ppCD3pppCD4pppCD127pCD45RApCCR7ppCD31ppCD28ppIL6RpCD38ppCD95ppCD27ppp	**CD38+ RTE Naive CD4+ Tcell**	**RTE Naive CD4+ Tcell**	**Naive CD4+ Tcell**	**CD4+ Tcell**
6	**c05**	**cd4**	CD45ppCD3pppCD4pppCD127pCCR7ppCD28ppIL6RpCD38ppCD95ppCD27ppp	**CD38+ CM CD4+ Tcell**	**CM CD4+ Tcell**	**CM CD4+ Tcell**	**CD4+ Tcell**
7	**c06**	**cd4**	CD45ppCD3pppCD4pppCD127pCCR7ppCCR4ppCD28ppIL6RpCD38ppCD95ppCD27ppp	**CD38+ Th2 CM CD4+ Tcell**	**Th2 CM CD4+ Tcell**	**CM CD4+ Tcell**	**CD4+ Tcell**
8	**c07**	**cd4**	CD45ppCD3pppCD4pppCD127pCCR7ppCCR6ppCCR4ppCD28pppIL6RpCD38ppPD.1pCD95pppCD27ppp	**PD1+ CD38+ Th17 CM CD4+ Tcell**	**Th17 CM CD4+ Tcell**	**CM CD4+ Tcell**	**CD4+ Tcell**
9	**c08**	**cd4**	CD45ppCD3pppCD4pppCCR4pCXCR3ppCD28pppHLA.DRppCD38ppPD.1ppCD95pppCD27pp	**HLADR+ CD38+ PD1+ Th1-Th2 EM CD4+ Tcell**	**Th1-Th2 EM CD4+ Tcell**	**EM CD4+ Tcell**	**CD4+ Tcell**
10	**c09**	**cd4**	CD45ppCD3pppCD4pppCD127pCCR4pCXCR3ppCD28ppCD57ppPD.1ppCD95pppCD27pp	**PD1+ CD57+ Th1 EM CD4+ Tcell**	**Th1 EM CD4+ Tcell**	**EM CD4+ Tcell**	**CD4+ Tcell**
11	**c10**	**cd4**	CD45ppCD3pppCD4pppCD25pCD127pCCR7ppCD31ppCCR6pCCR4ppCD28ppIL6RpCD95ppCD69pCD27pp	**CD25+ Th17 CM CD4+ Tcell**	**Th17 CM CD4+ Tcell**	**CM CD4+ Tcell**	**CD4+ Tcell**
12	**c11**	**cd4**	CD45ppCD3pppCD4pppCD127pCCR7ppCCR4pCXCR3ppCD28ppIL6RpCD38ppCD95ppCD27ppp	**CD38+ Th1-Th2 CM CD4+ Tcell**	**Th1 CM CD4+ Tcell**	**CM CD4+ Tcell**	**CD4+ Tcell**
13	**c12**	**cd4**	CD45ppCD3pppCD56pCD4pppCD25ppCCR7ppCCR4ppCXCR3ppCD28ppIL6RpCD38pppPD.1pCD95pppCD27ppp	**PD1+ CD38+ CD25+ Th1 CM CD4+ Tcell**	**Th1-Th2 CM CD4+ Tcell**	**CM CD4+ Tcell**	**CD4+ Tcell**
14	**c13**	**cd4**	CD45ppCD3pppCD4pppCCR7pCCR4pCXCR3ppCD28ppIL6RpCD38ppPD.1ppCD95pppCD27pp	**PD1+ CD38+ Th1 CM CD4+ Tcell**	**Th1 CM CD4+ Tcell**	**CM CD4+ Tcell**	**CD4+ Tcell**
15	**c14**	**cd4**	CD45ppCD3pppCD4pppCD127pCCR7pCCR4ppCXCR3ppCD28ppIL6RpPD.1ppCD95pppCD27pp	**PD1+ Th1-Th2 CM CD4+ Tcell**	**Th1-Th2 CM CD4+ Tcell**	**CM CD4+ Tcell**	**CD4+ Tcell**
16	**c15**	**cd4**	CD45ppCD3pppCD4pppCD127pCD45RApCCR7ppCD28ppIL6RpCD38pCD95pCD27ppp	**Naive CD4+ Tcell**	**Naive CD4+ Tcell**	**Naive CD4+ Tcell**	**CD4+ Tcell**
17	**c16**	**cd4**	CD45pCD3pppCD4pppCD127pCD45RApCCR7ppCD28ppIL6RpCD38pCD95pCD27pp	**Naive CD4+ Tcell**	**Naive CD4+ Tcell**	**Naive CD4+ Tcell**	**CD4+ Tcell**
18	**c17**	**cd4**	CD45ppCD3pppCD4pppCD25pCD127pCD45RApCCR7ppCXCR3ppCD28ppIL6RpCD38ppCD95pCD27pp	**CD38+ CD25+ Naive CD4+ Tcell**	**Naive CD4+ Tcell**	**Naive CD4+ Tcell**	**CD4+ Tcell**
19	**c18**	**cd4**	CD45ppCD3pppCD4pppCD127pCCR7ppCXCR3ppCD28ppIL6RpCD95ppCD27pp	**Th1 CM CD4+ Tcell**	**Th1 CM CD4+ Tcell**	**CM CD4+ Tcell**	**CD4+ Tcell**
20	**c19**	**cd4**	CD45ppCD3pppCD4pppCD25pCD127pCCR7ppCCR4ppCXCR3ppCD28ppIL6RpCD95pppCD27pp	**CD25+ Th1-Th2 CM CD4+ Tcell**	**Th1-Th2 CM CD4+ Tcell**	**CM CD4+ Tcell**	**CD4+ Tcell**
21	**c20**	**cd4**	CD45ppCD3pppCD4pppCD25pCD127pCXCR3pCD28ppPD.1ppCD95ppCD27pp	**PD1+ CD25+ Th1 EM CD4+ Tcell**	**Th1 EM CD4+ Tcell**	**EM CD4+ Tcell**	**CD4+ Tcell**
22	**c21**	**cd4**	CD45ppCD3pppCD4pppCD25pCD127pCD45RApCCR7ppCD31ppCD28ppIL6RpCD38pCD95pCD27ppp	**RTE CD25+ Naive CD4+ Tcell**	**RTE Naive CD4+ Tcell**	**Naive CD4+ Tcell**	**CD4+ Tcell**
23	**c22**	**cd4**	CD45ppCD3pppCD4pppCD25pCD127pCD45RApCCR7ppCD28ppIL6RpCD38pCD95pCD27pp	**CD25+ Naive CD4+ Tcell**	**Naive CD4+ Tcell**	**Naive CD4+ Tcell**	**CD4+ Tcell**
24	**c23**	**cd4**	CD45ppCD3pppCD4pppCD127pCCR7ppCD28ppIL6RpCD95ppCD27pp	**CM CD4+ Tcell**	**CM CD4+ Tcell**	**CM CD4+ Tcell**	**CD4+ Tcell**
25	**c24**	**cd4**	CD45ppCD3pppCD4pppCD25ppCD127pCCR7ppCXCR3ppCD28ppIL6RpCD95ppCD27pp	**CD25+ Th1 CM CD4+ Tcell**	**Th1 CM CD4+ Tcell**	**CM CD4+ Tcell**	**CD4+ Tcell**
26	**c25**	**cd4**	CD45ppCD3pppCD4pppCD127pCCR7ppCXCR3ppCD28ppIL6RpPD.1ppCD95ppCD27pp	**PD1+ Th1 CM CD4+ Tcell**	**Th1 CM CD4+ Tcell**	**CM CD4+ Tcell**	**CD4+ Tcell**
27	**c26**	**cd4**	CD45ppCD3pppCD4pppCD25pCD127ppCCR7pCXCR3ppCD28ppCD95ppCD27pp	**CD25+ Th1 CM CD4+ Tcell**	**Th1 CM CD4+ Tcell**	**CM CD4+ Tcell**	**CD4+ Tcell**
28	**c27**	**cd4**	CD45pCD3pppCD4pppCD127pCD45RApCCR7ppCD31ppCD28ppIL6RpCD38pCD27ppp	**RTE Naive CD4+ Tcell**	**RTE Naive CD4+ Tcell**	**Naive CD4+ Tcell**	**CD4+ Tcell**
29	**c28**	**cd4**	CD45ppCD3pppCD4pppCD25ppCD45RApCCR7ppCD31pCD28ppCD38pCD95pCD27pp	**Naive CD4+ Treg**	**Naive CD4+ Treg**	**Naive CD4+ Treg**	**CD4+ Treg**
30	**c29**	**cd4**	CD45ppCD3pppCD4pppCD25pCD127pCCR7ppCD28ppIL6RpCD38pCD95ppCD27ppp	**CD25+ CM CD4+ Tcell**	**CM CD4+ Tcell**	**CM CD4+ Tcell**	**CD4+ Tcell**
31	**c30**	**cd4**	CD45ppCD3pppCD4pppCD25pCD127pCCR7ppCCR6ppCD28ppIL6RpCD95ppCD27ppp	**CD25+ Th17+1 CM CD4+ Tcell**	**Th17+1 CM CD4+ Tcell**	**CM CD4+ Tcell**	**CD4+ Tcell**
32	**c31**	**cd4**	CD45ppCD3pppCD4pppCD25pCD127ppCCR7ppCCR6ppCXCR3ppCD28ppIL6RpCD95ppCD27ppp	**CD25+ CM CD4+ Tcell**	**Th17+1 CM CD4+ Tcell**	**CM CD4+ Tcell**	**CD4+ Tcell**
33	**c32**	**cd4**	CD45ppCD3ppCD4pppCD25pCD127pCCR6ppCXCR3pCD28ppCD95ppCD27pp	**CD25+ Th17+1 EM CD4+ Tcell**	**Th17+1 EM CD4+ Tcell**	**EM CD4+ Tcell**	**CD4+ Tcell**
34	**c33**	**cd4**	CD45ppCD3pppCD4pppCD127pCD45RApCCR7ppCD31ppCD28pCD38pCD27pp	**RTE Naive CD4+ Tcell**	**RTE Naive CD4+ Tcell**	**Naive CD4+ Tcell**	**CD4+ Tcell**
35	**c34**	**cd4**	CD45ppCD3pppCD4ppCD127pCD45RApCCR7pCD28pCD38pCD27pp	**Naive CD4+ Tcell**	**Naive CD4+ Tcell**	**Naive CD4+ Tcell**	**CD4+ Tcell**
36	**c35**	**cd4**	CD3ppCD4ppCD127pCCR7ppCD28ppCD95pCD27pp	**CM CD4+ Tcell**	**CM CD4+ Tcell**	**CM CD4+ Tcell**	**CD4+ Tcell**
37	**c36**	**cd4**	CD45ppCD3pppCD4pppCD25ppCCR7ppCD28ppCD95ppCD27ppp	**CM CD4+ Treg**	**CM CD4+ Treg**	**CM CD4+ Treg**	**CD4+ Treg**
38	**c37**	**cd4**	CD45ppCD3pppCD4pppCD25ppCD127pCCR7ppCCR4pCD28ppIL6RpCD95ppCD27ppp	**CD25+ Th2 CM CD4+ Tcell**	**Th2 CM CD4+ Tcell**	**CM CD4+ Tcell**	**CD4+ Tcell**
39	**c38**	**cd4**	CD45ppCD3pppCD4pppCD25pCD127pCCR7ppCCR6ppCCR4pCD28ppIL6RpCD95ppCD27ppp	**Th17 CM CD4+ Tcell**	**Th17 CM CD4+ Tcell**	**CM CD4+ Tcell**	**CD4+ Tcell**
40	**c39**	**cd4**	CD45ppCD3pppCD4pppCD25pCD127pCCR7ppCCR6ppCCR4ppCD28ppIL6RpCD95ppCD27pp	**CD25+ Th17 CM CD4+ Tcell**	**Th17 CM CD4+ Tcell**	**CM CD4+ Tcell**	**CD4+ Tcell**
41	**c40**	**cd4**	CD45ppCD3pppCD4pppCD25pCD127ppCCR7pCCR6ppCCR4ppCXCR3ppCD28ppIL6RpCD95pppCD27pp	**CD25+ Th17 CM CD4+ Tcell**	**Th17 CM CD4+ Tcell**	**CM CD4+ Tcell**	**CD4+ Tcell**
42	**c41**	**cd4**	CD45ppCD3pppCD4pppCD25pCD127ppCCR7pCCR6ppCXCR3ppCD28ppCD95ppCD27pp	**CD25+ Th17+1 CM CD4+ Tcell**	**Th17+1 CM CD4+ Tcell**	**CM CD4+ Tcell**	**CD4+ Tcell**
43	**c42**	**cd4**	CD45ppCD3pppCD56pTCRgdpCD45RApCXCR3pSLAMF7pCD57pppCX3CR1pCD95pp	**CD57+ Th1 EM DN Tcell**	**Th1 EM DN Tcell**	**EM DN Tcell**	**DN Tcell**
44	**c43**	**cd4**	CD45ppCD3ppCD4ppCD127pCCR7pCXCR3pCD28ppCD95ppCD27pp	**Th1 CM CD4+ Tcell**	**Th1 CM CD4+ Tcell**	**CM CD4+ Tcell**	**CD4+ Tcell**
45	**c44**	**cd4**	CD45ppCD3pppCD4pppCD127pCCR7ppCCR4pCD28ppIL6RpPD.1ppCD95pppCD27pp	**PD1+ Th2 CM CD4+ Tcell**	**Th2 CM CD4+ Tcell**	**CM CD4+ Tcell**	**CD4+ Tcell**
46	**c45**	**cd4**	CD45ppCD3pppCD4pppCD25ppCD127pCCR7ppCCR4ppCD28ppIL6RpCD95ppCD27pp	**CD25+ Th2 CM CD4+ Tcell**	**Th2 CM CD4+ Tcell**	**CM CD4+ Tcell**	**CD4+ Tcell**
47	**c46**	**cd4**	CD45ppCD3pppCD4pppCD127pCCR7ppCCR4ppCD28ppIL6RpCD95ppCD27pp	**Th2 CM CD4+ Tcell**	**Th2 CM CD4+ Tcell**	**CM CD4+ Tcell**	**CD4+ Tcell**
48	**c47**	**cd4**	CD45ppCD3pppCD4pppCD25ppCCR7ppCCR6ppCCR4ppCD28ppIL6RpCD95pppCD27ppp	**CCR6+ CCR4+ CM CD4+ Treg**	**CCR6+ CCR4+ CM CD4+ Treg**	**CM CD4+ Treg**	**CD4+ Treg**
49	**c48**	**cd4**	CD45ppCD3pppCD4pppCD127pCCR7ppCCR6ppCCR4ppCD28ppIL6RpCD95pppCD27pp	**Th17 CM CD4+ Tcell**	**Th17 CM CD4+ Tcell**	**CM CD4+ Tcell**	**CD4+ Tcell**
50	**c49**	**cd4**	CD45ppCD3pppCD4pppCD25pCD127pCCR7pCCR6ppCD28ppPD.1pCD95ppCD27pp	**PD1+ CD25+ Th17+1 CM CD4+ Tcell**	**Th17+1 CM CD4+ Tcell**	**CM CD4+ Tcell**	**CD4+ Tcell**
51	**c50**	**cd4**	CD45ppCD3pppCD4pppCD25pCD127pCCR6ppCCR4ppCXCR3ppCD28ppIL6RpPD.1pCD95ppp	**PD1+ CD25+ Th17 EM CD4+ Tcell**	**Th17 EM CD4+ Tcell**	**EM CD4+ Tcell**	**CD4+ Tcell**
52	**c51**	**cd4**	CD45ppCD3pppCD4pppCD127pCCR7pCCR6ppCCR4ppCXCR3ppCD28ppIL6RpPD.1ppCD95pppCD27pp	**PD1+ Th17-Th17+1 CM CD4+ Tcell**	**Th17-Th17+1 CM CD4+ Tcell**	**CM CD4+ Tcell**	**CD4+ Tcell**
53	**c52**	**cd4**	CD45ppCD3pppCD56pTCRgdppCD45RAppCD31ppSLAMF7pCD38pCD57pppPD.1pCX3CR1pCD95pp	**PD1+ CD57+ TEMRA TCRgd+ DN Tcell**	**TEMRA TCRgd+ DN Tcell**	**TCRgd+ DN Tcell**	**DN Tcell**
54	**c53**	**cd4**	CD45ppCD3pppTCRgdpCD45RApCD31pCXCR3pSLAMF7pCD57pppPD.1pCX3CR1pCD95ppCD27pp	**PD1+ CD57+ SLAMF7+ Th1 EM CD4+ Tcell**	**Th1 EM DN Tcell**	**EM DN Tcell**	**DN Tcell**
55	**c54**	**cd4**	CD45ppCD3ppCD4ppCD25pCD127pCCR4ppCD28ppCD95ppCD27pp	**CD25+ Th2 EM CD4+ Tcell**	**Th2 EM CD4+ Tcell**	**EM CD4+ Tcell**	**CD4+ Tcell**
56	**c55**	**cd4**	CD45ppCD3pppCD4pppCD127pCCR7pCCR4ppCD28ppIL6RpPD.1ppCD95pppCD27pp	**PD1+ Th2 CM CD4+ Tcell**	**Th2 CM CD4+ Tcell**	**CM CD4+ Tcell**	**CD4+ Tcell**
57	**c56**	**cd4**	CD45ppCD3pppCD4pppCD25ppCD127ppCCR7ppCCR6pppCCR4ppCD28ppIL6RpCD95pppCD27ppp	**CD25+ Th17 CM CD4+ Tcell**	**Th17 CM CD4+ Tcell**	**CM CD4+ Tcell**	**CD4+ Tcell**
58	**c57**	**cd4**	CD45ppCD3pppCD4pppCD25ppCD127ppCCR7pCCR6ppCCR4ppCD28ppIL6RpCD95pppCD27pp	**CD25+ Th17 CM CD4+ Tcell**	**Th17 CM CD4+ Tcell**	**CM CD4+ Tcell**	**CD4+ Tcell**
59	**c58**	**cd4**	CD45ppCD3pppCD4pppCD127pCCR7ppCCR6ppCCR4ppCD28ppIL6RpPD.1ppCD95pppCD27pp	**PD1+ Th17 CM CD4+ Tcell**	**Th17 CM CD4+ Tcell**	**CM CD4+ Tcell**	**CD4+ Tcell**
60	**c59**	**cd4**	CD45ppCD3pppCD4pppCD25pCD127ppCCR7pCCR6ppCXCR3ppCD28ppPD.1ppCD95pppCD27pp	**PD1+ CD25+ Th17+1 CM CD4+ Tcell**	**Th17+1 CM CD4+ Tcell**	**CM CD4+ Tcell**	**CD4+ Tcell**
61	**c60**	**cd4**	CD45ppCD3pppCD4pppCD25pCD127ppCCR6ppCXCR3pCD28ppPD.1pCD95pp	**PD1+ CD25+ Th17+1 EM CD4+ Tcell**	**Th17+1 EM CD4+ Tcell**	**EM CD4+ Tcell**	**CD4+ Tcell**
62	**c61**	**cd4**	CD45ppCD3pppCD56pCD4pppCD8ppCD45RApCXCR3pSLAMF7pCD57pppCX3CR1pCD95pp	**CD57+ Th1 EM DP Tcell**	**Th1 EM DP Tcell**	**EM DP Tcell**	**DP Tcell**
63	**c62**	**cd4**	CD45ppCD3pppCD56ppCD4pppCD127pCD45RApCXCR3pSLAMF7pCD57pppCD95pp	**CD57+ Th1 EM CD4+ NKT**	**Th1 EM CD4+ NKT**	**EM CD4+ NKT**	**CD4+ NKT**
64	**c63**	**cd4**	CD45ppCD3pppCD4pppCD127pCXCR3pCD28ppPD.1ppCD95ppp	**PD1+ Th1 EM CD4+ Tcell**	**Th1 EM CD4+ Tcell**	**EM CD4+ Tcell**	**CD4+ Tcell**
65	**c64**	**cd4**	CD45ppCD3pppCD4pppCD25pCD127ppCCR4pCXCR3ppCD28ppCD95pp	**CD25+ Th1 EM CD4+ Tcell**	**Th1 EM CD4+ Tcell**	**EM CD4+ Tcell**	**CD4+ Tcell**
66	**c65**	**cd4**	CD45ppCD3pppCD4pppCD25ppCD127pCCR7pCCR4ppCD28ppIL6RpPD.1pCD95pppCD27pp	**PD1+ CD25+ Th2 CM CD4+ Tcell**	**Th2 CM CD4+ Tcell**	**CM CD4+ Tcell**	**CD4+ Tcell**
67	**c66**	**cd4**	CD45ppCD3pppCD4pppCD25ppCCR7pCCR4ppCXCR3pCD28ppPD.1pCD95pppCD27pp	**PD1+ CXCR3+ CCR4+ CM CD4+ Treg**	**CXCR3+ CCR4+ CM CD4+ Treg**	**CM CD4+ Treg**	**CD4+ Treg**
68	**c67**	**cd4**	CD45ppCD3pppCD4pppCD25ppCCR7pCCR6ppCCR4ppCXCR3ppCD28ppIL6RpCD95pppCD27ppp	**CXCR3+ CCR4+ CM CD4+ Treg**	**CXCR3+ CCR4+ CM CD4+ Treg**	**CM CD4+ Treg**	**CD4+ Treg**
69	**c68**	**cd4**	CD45ppCD3pppCD56pCD4pppCD25ppCCR7pCCR6ppCCR4ppCD28pppIL6RpCD38ppPD.1pCD95pppCD27pp	**PD1+ CD38+ CCR6+ CCR4+ CM CD4+ Treg**	**CCR6+ CCR4+ CM CD4+ Treg**	**CM CD4+ Treg**	**CD4+ Treg**
70	**c69**	**cd4**	CD45ppCD3pppCD4pppCD25pCD127pCCR6pppCCR4ppCD28ppIL6RpPD.1ppCD95pppCD27pp	**PD1+ CD25+ Th17 EM CD4+ Tcell**	**Th17 EM CD4+ Tcell**	**EM CD4+ Tcell**	**CD4+ Tcell**
71	**c70**	**cd4**	CD45ppCD3pppCD4pppCD25ppCD127ppCCR6pppCCR4ppCD28ppIL6RpPD.1ppCD95ppp	**PD1+ CD25+ Th17 EM CD4+ Tcell**	**Th17 EM CD4+ Tcell**	**EM CD4+ Tcell**	**CD4+ Tcell**
72	**c71**	**cd4**	CD45ppCD3pppCD4pppCXCR3pSLAMF7pCD57pppPD.1pCX3CR1pCD95pp	**PD1+ CD57+ SLAMF7+ Th1 EM CD4+ Tcell**	**Th1 EM CD4+ Tcell**	**EM CD4+ Tcell**	**CD4+ Tcell**
73	**c72**	**cd4**	CD45ppCD3pppCD4pppCD127pCXCR3pCD28ppSLAMF7pCD57pppPD.1ppCD95pp	**PD1+ CD57+ SLAMF7+ Th1 EM CD4+ Tcell**	**Th1 EM CD4+ Tcell**	**EM CD4+ Tcell**	**CD4+ Tcell**
74	**c73**	**cd4**	CD45ppCD3pppCD56pCD4pppCD127pCXCR3pSLAMF7pPD.1pCX3CR1pCD95pp	**PD1+ SLAMF7+ Th1 EM CD4+ Tcell**	**Th1 EM CD4+ Tcell**	**EM CD4+ Tcell**	**CD4+ Tcell**
75	**c74**	**cd4**	CD45ppCD3pppCD4pppCD127pCCR4ppCXCR3pCD28ppHLA.DRppCD95ppp	**HLADR+ Th1-Th2 EM CD4+ Tcell**	**Th1-Th2 EM CD4+ Tcell**	**EM CD4+ Tcell**	**CD4+ Tcell**
76	**c75**	**cd4**	CD45ppCD3pppCD4pppCD25ppCD127pCCR7pCCR4ppCD28ppIL6RpCD95ppp	**CD25+ Th2 CM CD4+ Tcell**	**Th2 CM CD4+ Tcell**	**CM CD4+ Tcell**	**CD4+ Tcell**
77	**c76**	**cd4**	CD45ppCD3pppCD56pCD4pppCD25ppCCR4ppCXCR3pCD28pppIL6RpHLA.DRpppCD38pPD.1pCD95pppCD27pp	**HLADR+ CD38+ PD1+ CD25+ CCR4+ CXCR3+ E**	**CCR4+ CXCR3+ EM CD4+ Treg**	**EM CD4+ Treg**	**CD4+ Treg**
78	**c77**	**cd4**	CD45ppCD3pppCD56pCD4pppCD25pppCCR6ppCCR4pppCD28ppIL6RpHLA.DRppPD.1pCD95pppCD27pp	**HLADR+ PD1+ CCR6+ CCR4+ EM CD4+ Treg**	**CCR6+ CCR4+ EM CD4+ Treg**	**EM CD4+ Treg**	**CD4+ Treg**
79	**c78**	**cd4**	CD45ppCD3pppCD4pppCD25pppCCR6ppCCR4ppCD28ppIL6RpCD95pppCD27pp	**CCR6+ CCR4+ EM CD4+ Treg**	**CCR6+ CCR4+ EM CD4+ Treg**	**EM CD4+ Treg**	**CD4+ Treg**
80	**c79**	**cd4**	CD45ppCD3pppCD4pppCD25ppCD127pCCR6ppCCR4ppCD28ppIL6RpCD95ppp	**CD25+ Th17 EM CD4+ Tcell**	**Th17 EM CD4+ Tcell**	**EM CD4+ Tcell**	**CD4+ Tcell**
81	**c80**	**cd4**	CD45ppCD3pppCD4pppCD25pCD127pCCR6ppCCR4ppCD28ppIL6RpHLA.DRppPD.1pCD95ppp	**HLADR+ PD1+ CD25+ Th17 EM CD4+ Tcell**	**Th17 EM CD4+ Tcell**	**EM CD4+ Tcell**	**CD4+ Tcell**

	A	B	C	D	E	F	G
82	**cluster**	**panel**	phenotype	**Full label**	**Differentiation**	**Memory Subset**	**Cell population**
83	**c01**	**cd8**	CD45ppCD56ppCD8pppCD16pCD45RAppCD31pSLAMF7pCD38ppCD57pppCX3CR1pCD95p	**CD57+CD31+CD38+CD8+CD16+CD56dim NI**	**CD8+CD16+CD56dim NKcell**	**CD8+CD16+CD56dim NKcell**	**CD56dim NKcell**
84	**c02**	**cd8**	CD45ppCD56ppCD8ppCD16pCD45RAppCD31ppSLAMF7pCD38ppCD57pppCX3CR1pCD95p	**CD57+CD31+CD38+CD8+CD16+CD56dim NI**	**CD8+CD16+CD56dim NKcell**	**CD8+CD16+CD56dim NKcell**	**CD56dim NKcell**
85	**c03**	**cd8**	CD45pppCD3pppCD56ppCD8pppCD127pCD45RAppCCR7ppCD31ppCXCR3pCD28ppSLAMF7pCD38ppCD57pppCX3CR1pCD95ppCD27pp	**CD57+CD31+CD38+CXCR3+Naive CD8+NKT**	**CXCR3+Naive CD8+NKTcell**	**Naive CD8+NKTcell**	**CD8+NKTcell**
86	**c04**	**cd8**	CD45ppCD3pppCD8pppCD127pCD45RAppCCR7ppCD31ppCXCR3pCD28ppCD38pCD27ppp	**Naive CD8+Tcell**	**Naive CD8+Tcell**	**Naive CD8+Tcell**	**CD8+Tcell**
87	**c05**	**cd8**	CD45ppCD56ppCD8ppCD16pCD45RAppCD31pSLAMF7pCD38ppCD57pppCX3CR1pCD95p	**CD57+CD31+CD38+CD8+CD16+CD56dim NI**	**CD8+CD16+CD56dim NKcell**	**CD8+CD16+CD56dim NKcell**	**CD56dim NKcell**
88	**c06**	**cd8**	CD45ppCD3ppCD56ppCD4pCD8ppCD45RAppCD31ppSLAMF7pCD38ppCD57ppCX3CR1pCD95ppCD27pp	**CD57+CD31+CD38+TEMRA DP NKTcell**	**TEMRA DP NKTcell**	**TEMRA DP NKTcell**	**DP NKTcell**
89	**c07**	**cd8**	CD3pppCD8pppCD127pCD45RAppCCR7ppCD31ppCXCR3pCD28ppCD38pCD27ppp	**Naive CD8+Tcell**	**Naive CD8+Tcell**	**Naive CD8+Tcell**	**CD8+Tcell**
90	**c08**	**cd8**	CD45ppCD3pppCD8pppCD127pCD45RApCCR7ppCD31ppCXCR3ppCD28ppCD95ppCD27pp	**Naive CD8+Tcell**	**Naive CD8+Tcell**	**Naive CD8+Tcell**	**CD8+Tcell**
91	**c09**	**cd8**	CD45ppCD3pppCD4pppCD8pppCD127ppCD45RApCCR7ppCD31ppCXCR3ppCD28ppIL6RpCD38pCD95ppCD27ppp	**CD38+Tc1 CM DP Tcell**	**Tc1 CM DP Tcell**	**CM DP Tcell**	**DP Tcell**
92	**c10**	**cd8**	CD45ppCD3pppCD8pppCD127pCD45RApCCR7ppCD31ppCXCR3ppCD28ppCD38ppCD95ppCD27ppp	**CD38+Tc1 CM CD8+Tcell**	**Tc1 CM CD8+Tcell**	**CM CD8+Tcell**	**CD8+Tcell**
93	**c11**	**cd8**	CD45ppCD56ppCD8ppCD16pCD45RAppSLAMF7pCD38pCD57pppCX3CR1pCD95p	**CD57+CD38+CD8+CD16+CD56dim NK cell**	**CD8+CD16+CD56dim NKcell**	**CD8+CD16+CD56dim NKcell**	**CD56dim NKcell**
94	**c12**	**cd8**	CD45pCD3ppCD8pppCD127pCD31pCXCR3pCD28pCD95ppCD27p	**Tc1 EM CD8+Tcell**	**Tc1 EM CD8+Tcell**	**EM CD8+Tcell**	**CD8+Tcell**
95	**c13**	**cd8**	CD45pCD3ppCD8pppCD127pCD45RApCCR7pCD31ppCXCR3pCD28pCD27pp	**Tc1 EM CD8+Tcell**	**Tc1 EM CD8+Tcell**	**EM CD8+Tcell**	**CD8+Tcell**
96	**c14**	**cd8**	CD45ppCD3pppCD8pppCD25ppCD127pCCR7pCCR4ppCD28ppIL6RpCD95ppCD27pp	**Tc2 CM CD8+Tcell**	**Tc2 CM CD8+Tcell**	**CM CD8+Tcell**	**CD8+Tcell**
97	**c15**	**cd8**	CD45ppCD3pppCD8pppCD127pCCR7pCXCR3ppCD28ppCD95ppCD27pp	**Tc1 CM CD8+Tcell**	**Tc1 CM CD8+Tcell**	**CM CD8+Tcell**	**CD8+Tcell**
98	**c16**	**cd8**	CD45ppCD3pppCD4pppCD8pppCD25ppCD127pCCR7pCCR4ppCD28ppIL6RpCD95pppCD27pp	**CM CD4+CD8+Tcell**	**Tc2 CM DP Tcell**	**CM DP Tcell**	**DP Tcell**
99	**c17**	**cd8**	CD45ppCD56ppCD8ppCD16pCD45RAppSLAMF7pCD38pCD57pppCX3CR1pCD95pp	**CD38+CD57+CD8+CD16+CD56dim NK cell**	**CD8+CD16+CD56dim NKcell**	**CD8+CD16+CD56dim NKcell**	**CD56dim NKcell**
100	**c18**	**cd8**	CD45ppCD56ppCD8ppCD16pCD45RAppCD31ppSLAMF7pCD38pCD57pppCX3CR1pCD95p	**CD31+CD38+CD57+CD8+CD16+CD56dim NI**	**CD8+CD16+CD56dim NKcell**	**CD8+CD16+CD56dim NKcell**	**CD56dim NKcell**
101	**c19**	**cd8**	CD45ppCD3ppCD8pppCD45RApCD57pppCD95p	**CD57+EM CD8+Tcell**	**EM CD8+Tcell**	**EM CD8+Tcell**	**CD8+Tcell**
102	**c20**	**cd8**	CD45ppCD3pppCD8pppCD45RApCXCR3pSLAMF7pCD57pppCX3CR1pCD95ppCD27pp	**CD57+Tc1 EM CD8+Tcell**	**Tc1 EM CD8+Tcell**	**EM CD8+Tcell**	**CD8+Tcell**
103	**c21**	**cd8**	CD3pppCD8pppCD127pCCR7pCD31pCXCR3ppCD28ppCD95ppCD27pp	**Tc1 CM CD8+Tcell**	**Tc1 CM CD8+Tcell**	**CM CD8+Tcell**	**CD8+Tcell**
104	**c22**	**cd8**	CD45ppCD3pppCD8pppCD45RApCXCR3pSLAMF7pCD95ppCD27pp	**Tc1 TEMRA CD8+Tcell**	**Tc1 EM CD8+Tcell**	**EM CD8+Tcell**	**CD8+Tcell**
105	**c23**	**cd8**	CD45ppCD3pppCD8pppCD127pCD45RApCD31ppCXCR3ppCD95ppCD27pp	**Tc1 SLAMF7- CM CD8+Tcell**	**Tc1 EM CD8+Tcell**	**EM CD8+Tcell**	**CD8+Tcell**
106	**c24**	**cd8**	CD45ppCD3pppCD8pppCD127ppCCR7pCD31ppCCR6ppCXCR3ppCD28ppCD95ppCD27pp	**Tc17+1 CM CD8+Tcell**	**Tc17+1 CM CD8+Tcell**	**CM CD8+Tcell**	**CD8+Tcell**
107	**c25**	**cd8**	CD45ppCD3pppCD56ppCD8pppCD127ppCD31ppCCR6ppCXCR3pCD28ppSLAMF7pPD.1pCD95ppCD69pCD27pp	**PD1+CD69+Tc17+1 EM CD8+Tcell**	**Tc17+1 EM CD8+Tcell**	**EM CD8+Tcell**	**CD8+Tcell**
108	**c26**	**cd8**	CD45ppCD3pppCD56ppCD4pppCD8ppCD127pCD45RApCXCR3pSLAMF7pCD57pppCD95pp	**CD57+Tc1 EM DP Tcell**	**Tc1 EM DP Tcell**	**EM DP Tcell**	**DP Tcell**
109	**c27**	**cd8**	CD45ppCD3pppCD56pTCRgdppCD8ppCD45RAppCD31ppSLAMF7pCD38pCD57pppCX3CR1pCD95pp	**CD57+TEMRA TCRgd Tcell**	**TEMRA TCRgd+CD8+Tcell**	**TEMRA TCRgd+CD8+Tcell**	**TCRgd+CD8+Tcell**
110	**c28**	**cd8**	CD3pppCD8pppCD45RAppCD31pSLAMF7pCD57pppCX3CR1pCD95pp	**CD57+TEMRA CD8+Tcell**	**TEMRA CD8+Tcell**	**TEMRA CD8+Tcell**	**CD8+Tcell**
111	**c29**	**cd8**	CD45ppCD3pppCD8pppCD31ppCXCR3pSLAMF7pCD57ppCD95pp	**CD57+Tc1 EM CD8+Tcell**	**Tc1 EM CD8+Tcell**	**EM CD8+Tcell**	**CD8+Tcell**
112	**c30**	**cd8**	CD45ppCD3pppCD56pCD8pppCD45RApCXCR3pSLAMF7pCX3CR1pCD95pp	**Tc1 EM CD8+Tcell**	**Tc1 EM CD8+Tcell**	**EM CD8+Tcell**	**CD8+Tcell**
113	**c31**	**cd8**	CD45ppCD3pppCD8pppCD127pCD31ppCXCR3ppCD28ppSLAMF7pCD95pp	**Tc1 EM CD8+Tcell**	**Tc1 EM CD8+Tcell**	**EM CD8+Tcell**	**CD8+Tcell**
114	**c32**	**cd8**	CD45ppCD3pppCD8pppCD127pCD45RAppCD31ppCXCR3pCD28ppSLAMF7pPD.1pCD95ppCD27pp	**PD1+Tc1 TEMRA CD8+Tcell**	**Tc1 TEMRA CD8+Tcell**	**TEMRA CD8+Tcell**	**CD8+Tcell**
115	**c33**	**cd8**	CD45ppCD3pppCD8pppCD127pCD31ppCXCR3ppCD28ppSLAMF7pCD95ppCD27pp	**Tc1 EM CD8+Tcell**	**Tc1 EM CD8+Tcell**	**EM CD8+Tcell**	**CD8+Tcell**
116	**c34**	**cd8**	CD45ppCD3pppCD8pppCD127ppCD31ppCCR6ppCXCR3ppCD28ppCD95pp	**Tc17+1 EM CD8+Tcell**	**Tc17+1 EM CD8+Tcell**	**EM CD8+Tcell**	**CD8+Tcell**
117	**c35**	**cd8**	CD45ppCD3pppCD56ppCD8pppCD127ppCD31ppCCR6ppCXCR3pCD28ppSLAMF7pPD.1pCD95ppCD69ppCD27pp	**PD1+CD69+Tc17+1 EM CD8+Tcell**	**Tc17+1 EM CD8+Tcell**	**EM CD8+Tcell**	**CD8+Tcell**
118	**c36**	**cd8**	CD45ppCD3pppCD4pppCD8ppCD45RApCXCR3pSLAMF7pCD57pppCX3CR1pCD95pp	**Tc1 EM CD4+CD8+Tcell**	**Tc1 EM DP Tcell**	**EM DP Tcell**	**DP Tcell**
119	**c37**	**cd8**	CD45pppCD3pppCD56pCD4pppCD8pppCD127pCD45RAppCCR7ppCD31ppCXCR3pCD28ppSLAMF7pCD38pCD57pppCX3CR1pCD95ppCD27pp	**CD57+Naive DP Tcell**	**Tc1 Naive DP Tcell**	**Naive DP Tcell**	**DP Tcell**
120	**c38**	**cd8**	CD45ppCD3pppTCRgdpCD8pppCD45RAppCD31ppSLAMF7pCD38pCD57pppPD.1ppCX3CR1pCD95pp	**PD1+CD57+TEMRA CD8+Tcell**	**TEMRA TCRgd+CD8+Tcell**	**TEMRA TCRgd+CD8+Tcell**	**TCRgd+CD8+Tcell**
121	**c39**	**cd8**	CD45ppCD3pppCD8pppCD45RAppCD31ppSLAMF7pCD57ppCX3CR1pCD95pp	**CD57+TEMRA CD8+Tcell**	**TEMRA CD8+Tcell**	**TEMRA CD8+Tcell**	**CD8+Tcell**
122	**c40**	**cd8**	CD45ppCD3pppCD8pppCD45RAppCD31ppCXCR3pSLAMF7pCX3CR1pCD95pp	**Tc1 TEMRA CD8+Tcell**	**Tc1 TEMRA CD8+Tcell**	**TEMRA CD8+Tcell**	**CD8+Tcell**
123	**c41**	**cd8**	CD45ppCD3pppCD8pppCD45RApCD31ppCXCR3pSLAMF7pHLA.DRppCD38ppCD95ppp	**HLADR+CD38+Tc1 EM CD8+Tcell**	**Tc1 EM CD8+Tcell**	**EM CD8+Tcell**	**CD8+Tcell**
124	**c42**	**cd8**	CD45ppCD3pppCD8pppCD31ppCXCR3pSLAMF7pPD.1ppCX3CR1pCD95ppCD27pp	**PD1+Tc1 EM CD8+Tcell**	**Tc1 EM CD8+Tcell**	**EM CD8+Tcell**	**CD8+Tcell**
125	**c43**	**cd8**	CD45ppCD3pppCD8pppCD127pCCR7pCD31ppCXCR3pCD28ppCD95ppCD27pp	**Tc1 CM CD8+Tcell**	**Tc1 CM CD8+Tcell**	**CM CD8+Tcell**	**CD8+Tcell**
126	**c44**	**cd8**	CD45ppCD3pppCD8pppCD127ppCD31ppCCR6ppCXCR3pCD28ppSLAMF7pPD.1pCD95ppCD27pp	**PD1+Tc17+1 EM CD8+Tcell**	**Tc17+1 EM CD8+Tcell**	**EM CD8+Tcell**	**CD8+Tcell**
127	**c45**	**cd8**	CD45ppCD3pppCD56pCD8pppCD127ppCCR6ppCXCR3pCD28ppPD.1ppCD95pp	**PD-1+Tc17+1 EM CD8+Tcell**	**Tc17+1 EM CD8+Tcell**	**EM CD8+Tcell**	**CD8+Tcell**
128	**c46**	**cd8**	CD45ppCD3pppCD56ppCD8ppCD45RApCXCR3pSLAMF7pCD57pppCX3CR1pCD95pp	**CD57+CXCR3+EM CD8+NKTcell**	**CXCR3+EM CD8+NKTcell**	**EM CD8+NKTcell**	**CD8+NKTcell**
129	**c47**	**cd8**	CD45ppCD3pppCD56pCD8pppCD45RAppCD31ppSLAMF7pCD57pppCX3CR1pCD95pp	**CD31+CD57+TEMRA CD8+Tcell**	**TEMRA CD8+Tcell**	**TEMRA CD8+Tcell**	**CD8+Tcell**
130	**c48**	**cd8**	CD45ppCD3pppCD8pppCD45RApCD31ppCXCR3pSLAMF7pHLA.DRppCD38ppCD57pppCX3CR1pCD95pp	**HLADR+CD38+Tc1 EM CD8+Tcell**	**Tc1 EM CD8+Tcell**	**EM CD8+Tcell**	**CD8+Tcell**
131	**c49**	**cd8**	CD45ppCD3pppCD8pppCD45RApCD31ppCXCR3pSLAMF7pCD57ppCX3CR1pCD95ppCD27pp	**CD57+Tc1 EM CD8+Tcell**	**Tc1 EM CD8+Tcell**	**EM CD8+Tcell**	**CD8+Tcell**
132	**c50**	**cd8**	CD45ppCD3pppCD8pppCD45RApCD31ppCXCR3pSLAMF7pCX3CR1pCD95ppCD27pp	**CD57+Tc1 EM CD8+Tcell**	**Tc1 EM CD8+Tcell**	**EM CD8+Tcell**	**CD8+Tcell**
133	**c51**	**cd8**	CD45ppCD3pppCD8pppCD127pCD31ppCXCR3pCD28ppSLAMF7pPD.1ppCD95ppCD27pp	**PD1+Tc1 EM CD8+Tcell**	**Tc1 EM CD8+Tcell**	**EM CD8+Tcell**	**CD8+Tcell**
134	**c52**	**cd8**	CD45ppCD3pppCD8pppCD127pCCR7pCD31ppCXCR3ppCD28ppSLAMF7pPD.1ppCD95ppCD27pp	**PD1+Tc1 CM CD8+Tcell**	**Tc1 CM CD8+Tcell**	**CM CD8+Tcell**	**CD8+Tcell**
135	**c53**	**cd8**	CD45ppCD3pppCD8pppCD127pCXCR3ppCD28ppSLAMF7pCD95pp	**Tc1 EM CD8+Tcell**	**Tc1 EM CD8+Tcell**	**EM CD8+Tcell**	**CD8+Tcell**
136	**c54**	**cd8**	CD45ppCD3pppCD8pppCD127ppCCR6ppCXCR3pCD28ppPD.1pCD95ppCD27pp	**PD1+Tc17+1 CM CD8+Tcell**	**Tc17+1 CM CD8+Tcell**	**CM CD8+Tcell**	**CD8+Tcell**
137	**c55**	**cd8**	CD45ppCD3pppCD56ppCD8pppCD45RAppCXCR3pSLAMF7pCD57pppCX3CR1pCD95pp	**CD57+Tc1 TEMRA CD8+Tcell**	**Tc1 TEMRA CD8+Tcell**	**TEMRA CD8+Tcell**	**CD8+Tcell**
138	**c56**	**cd8**	CD45ppCD3pppCD56ppCD8pppCD45RApCD31ppCXCR3pSLAMF7pCD57pppCD95pp	**CD57+Tc1 EM CD8+Tcell**	**EM CD8+Tcell**	**EM CD8+Tcell**	**CD8+Tcell**
139	**c57**	**cd8**	CD45ppCD3pppCD8pppCD45RApCD31ppSLAMF7pCD57pppCX3CR1pCD95pp	**CD57+EM CD8+Tcell**	**EM CD8+Tcell**	**EM CD8+Tcell**	**CD8+Tcell**
140	**c58**	**cd8**	CD45ppCD3pppCD8pppCD45RAppCD31ppSLAMF7pCD57pppPD.1pCX3CR1pCD95ppCD27pp	**PD1+CD57+TEMRA CD8+Tcell**	**TEMRA CD8+Tcell**	**TEMRA CD8+Tcell**	**CD8+Tcell**
141	**c59**	**cd8**	CD45ppCD3pppCD56ppCD8pppCD127pCD45RApCD31ppCXCR3ppCD28pSLAMF7pCD95ppCD27pp	**Tc1 EM CD8+Tcell**	**Tc1 EM CD8+Tcell**	**EM CD8+Tcell**	**CD8+Tcell**
142	**c60**	**cd8**	CD45ppCD3pppCD8pppCD31ppSLAMF7pCD57pppPD.1ppCX3CR1pCD95ppCD27pp	**PD1+CD57+EM CD8+Tcell**	**EM CD8+Tcell**	**EM CD8+Tcell**	**CD8+Tcell**
143	**c61**	**cd8**	CD45ppCD3pppCD8pppCD127pCD31ppCXCR3pCD28ppSLAMF7pCD57ppPD.1ppCD95ppCD27pp	**PD1+Tc1 EM CD8+Tcell**	**Tc1 EM CD8+Tcell**	**EM CD8+Tcell**	**CD8+Tcell**
144	**c62**	**cd8**	CD45ppCD3pppCD8pppCD127pCCR7pCD31ppCCR4ppCXCR3ppCD28ppSLAMF7pPD.1ppCD95ppCD27pp	**PD1+Tc1-Tc2 CM CD8+Tcell**	**Tc1-Tc2 CM CD8+Tcell**	**CM CD8+Tcell**	**CD8+Tcell**
145	**c63**	**cd8**	CD45ppCD3pppCD8pppCD127pCXCR3ppCD28ppSLAMF7pPD.1ppCD95ppCD27pp	**PD1+Tc1 EM CD8+Tcell**	**Tc1 EM CD8+Tcell**	**EM CD8+Tcell**	**CD8+Tcell**
146	**c64**	**cd8**	CD45ppCD3pppCD8pppCD45RAppSLAMF7pCD57pppCX3CR1pCD95pp	**CD57+TEMRA CD8+Tcell**	**TEMRA CD8+Tcell**	**TEMRA CD8+Tcell**	**CD8+Tcell**
147	**c65**	**cd8**	CD45ppCD3pppCD8pppCD45RApSLAMF7pCD57pppCX3CR1pCD95pp	**EM CD8+Tcell**	**EM CD8+Tcell**	**EM CD8+Tcell**	**CD8+Tcell**
148	**c66**	**cd8**	CD45ppCD3pppCD8pppCD31ppCXCR3pSLAMF7pCD57pppCX3CR1pCD95ppCD27pp	**Tc1 EM CD8+Tcell**	**Tc1 EM CD8+Tcell**	**EM CD8+Tcell**	**CD8+Tcell**
149	**c67**	**cd8**	CD45ppCD3pppCD8pppCD45RApCXCR3pSLAMF7pCD57pppPD.1ppCX3CR1pCD95ppCD27pp	**TEMRA CD8+Tc1 cell**	**EM CD8+Tcell**	**EM CD8+Tcell**	**CD8+Tcell**
150	**c68**	**cd8**	CD45ppCD3pppCD8pppCD127pCD31ppCXCR3ppCD28ppSLAMF7pCD57ppCD95ppCD27pp	**CD57+Tc1 EM CD8+Tcell**	**Tc1 EM CD8+Tcell**	**EM CD8+Tcell**	**CD8+Tcell**
151	**c69**	**cd8**	CD45ppCD3pppCD8pppCD127pCD31ppCCR4ppCXCR3ppCD28ppSLAMF7pCD57pppPD.1ppCD95pppCD27pp	**PD1+CD57+Tc1-Tc2 EM CD8+Tcell**	**Tc1-Tc2 EM CD8+Tcell**	**EM CD8+Tcell**	**CD8+Tcell**
152	**c70**	**cd8**	CD45ppCD3pppCD8pppCXCR3ppCD28ppHLA.DRppCD38ppPD.1ppCD95pppCD27pp	**HLADR+CD38+Tc1 EM CD8+Tcell**	**Tc1 EM CD8+Tcell**	**EM CD8+Tcell**	**CD8+Tcell**
153	**c71**	**cd8**	CD45ppCD3pppCD8pppCD127pCCR4ppCXCR3ppCD28ppHLA.DRpPD.1ppCD95pppCD27pp	**PD1+Tc1-Tc2 EM CD8+Tcell**	**Tc1-Tc2 EM CD8+Tcell**	**EM CD8+Tcell**	**CD8+Tcell**
154	**c72**	**cd8**	CD45ppCD3pppCD8pppCD25ppCD127pCCR7pCCR6ppCCR4ppCD28ppIL6RpCD95pppCD27pp	**CD25+Tc17+1 CM CD8+Tcell**	**Tc17+1 CM CD8+Tcell**	**CM CD8+Tcell**	**CD8+Tcell**
155	**c73**	**cd8**	CD45ppCD3pppCD8pppCD45RApSLAMF7pCD57pppPD.1ppCX3CR1pCD95pp	**PD-1+CD57+TEMRA CD8+Tcell**	**TEMRA CD8+Tcell**	**TEMRA CD8+Tcell**	**CD8+Tcell**
156	**c74**	**cd8**	CD45ppCD3pppCD8pppCD45RAppSLAMF7pCD57pppCX3CR1pCD95ppCD27pp	**CD57+TEMRA CD8+Tcell**	**TEMRA CD8+Tcell**	**TEMRA CD8+Tcell**	**CD8+Tcell**
157	**c75**	**cd8**	CD45ppCD3pppCD8pppCD127pCXCR3ppCD28ppSLAMF7pCD57ppPD.1ppCD95ppCD27pp	**PD-1+CD57+Tc1 EM CD8+Tcell**	**Tc1 EM CD8+Tcell**	**EM CD8+Tcell**	**CD8+Tcell**
158	**c76**	**cd8**	CD45ppCD3pppCD8pppCD31ppCXCR3pCD28ppSLAMF7pCD57pppPD.1ppCD95ppCD27pp	**CD57+PD-1+Tc1 EM CD8+Tcell**	**Tc1 EM CD8+Tcell**	**EM CD8+Tcell**	**CD8+Tcell**
159	**c77**	**cd8**	CD45ppCD3pppCD8pppCD31ppCCR4pCXCR3ppCD28ppSLAMF7pHLA.DRppCD38ppCD57ppPD.1ppCD95pppCD27pp	**HLADR+CD38+PD1+CD57+Tc1-Tc2 EM CD8+**	**Tc1-Tc2 EM CD8+Tcell**	**EM CD8+Tcell**	**CD8+Tcell**
160	**c78**	**cd8**	CD45ppCD3pppCD8pppCD31ppCXCR3ppCD28ppSLAMF7pHLA.DRppCD38ppPD.1ppCD95pppCD27ppp	**HLADR+CD38+PD-1+Tc1 EM CD8+Tcell**	**Tc1 EM CD8+Tcell**	**EM CD8+Tcell**	**CD8+Tcell**
161	**c79**	**cd8**	CD45ppCD3pppCD8pppCD25pCD127pCCR7pCD31ppCCR4ppCD28ppCD95ppCD27pp	**CD31+CD25+Tc2 CM CD8+Tcell**	**Tc2 CM CD8+Tcell**	**CM CD8+Tcell**	**CD8+Tcell**
162	**c80**	**cd8**	CD45ppCD3pppCD8pppCD25ppCD127pCCR4ppCD28ppIL6RpCD95pppCD27p	**CD25+Tc2 EM CD8+Tcell**	**Tc2 EM CD8+Tcell**	**EM CD8+Tcell**	**CD8+Tcell**

	A	B	C	D	E	F	G
163	cluster	panel	phenotype	Full label	Differentiation	Memory Subset	Cell population
164	c01	nk	CD45ppCD56ppCD16pCD45RAppSLAMF7pCD38pCD57pppCX3CR1pCD95pp	CD57+ CD8- CD16+ CD56dim NK cell	CD8- CD16+ CD56dim NK cell	CD8- CD16+ CD56dim NK cell	CD56dim NK cell
165	c02	nk	CD45ppCD56ppCD16pCD45RAppSLAMF7pCD57pppCX3CR1pCD95p	CD57+ CD8- CD16+ CD56dim NK cell	CD8- CD16+ CD56dim NK cell	CD8- CD16+ CD56dim NK cell	CD56dim NK cell
166	c03	nk	CD45ppCD56ppCD45RApCD38pCD57pppCX3CR1pCD95p	CD57+ CD8- CD16- CD56dim NK cell	CD8- CD16- CD56dim NK cell	CD8- CD16- CD56dim NK cell	CD56dim NK cell
167	c04	nk	CD45ppCD56ppCD16pCD45RApCD31pSLAMF7pCD38ppCD57pppCX3CR1pCD95p	CD38+ CD57+ CD8- CD16+ CD56dim NK cell	CD8- CD16+ CD56dim NK cell	CD8- CD16+ CD56dim NK cell	CD56dim NK cell
168	c05	nk	CD45ppCD56ppCD16pCD45RAppSLAMF7pCD38pCD57ppCX3CR1pCD95pp	CD57+ CD8- CD16+ CD56dim NK cell	CD8- CD16+ CD56dim NK cell	CD8- CD16+ CD56dim NK cell	CD56dim NK cell
169	c06	nk	CD45ppCD56ppCD16pCD45RAppCD31pSLAMF7pCD38ppCX3CR1pCD95p	CD31+ CD38+ CD8- CD16+ CD56dim NK cell	CD8- CD16+ CD56dim NK cell	CD8- CD16+ CD56dim NK cell	CD56dim NK cell
170	c07	nk	CD45ppCD56ppCD16pCD45RAppCD31pSLAMF7pCD38ppCX3CR1pCD95p	CD31+ CD38+ CD8- CD16+ CD56dim NK cell	CD8- CD16+ CD56dim NK cell	CD8- CD16+ CD56dim NK cell	CD56dim NK cell
171	c08	nk	CD45ppCD56ppCD45RAppCXCR3pSLAMF7pCD95pp	CD8- CD16- CD56bright NK cell	CD8- CD16- CD56bright NK cell	CD8- CD16- CD56bright NK ce	CD56bright NK cell
172	c09	nk	CD45ppCD56ppCD45RAppCD31ppCXCR3pSLAMF7pCD38ppCD95pCD27pp	CD31+ CD38+ CD16- CD56bright NK cell	CD16- CD56bright NK cell	CD16- CD56bright NK cell	CD56bright NK cell
173	c10	nk	CD45ppCD56pppCD8ppCD45RApCD31ppCXCR3ppSLAMF7pCD38ppCD95pCD27pp	CD31+ CD38+ CD8+ CD16- CD56bright NK cell	CD8+ CD16- CD56bright NK cell	CD8+ CD16- CD56bright NK ce	CD56bright NK cell
174	c11	nk	CD45ppCD56ppCD16pCD45RAppSLAMF7pCD38ppCD57pppCX3CR1pCD95pp	CD38+ CD57+ CD8- CD16+ CD56dim NK cell	CD8- CD16+ CD56dim NK cell	CD8- CD16+ CD56dim NK cell	CD56dim NK cell
175	c12	nk	CD45ppCD56ppCD16pCD45RAppSLAMF7pCD38ppCD57pppCX3CR1ppCD95pp	CD38+ CD57+ CD8- CD16+ CD56dim NK cell	CD8- CD16+ CD56dim NK cell	CD8- CD16+ CD56dim NK cell	CD56dim NK cell
176	c13	nk	CD45ppCD56ppCD16pCD45RAppCD31pSLAMF7pCD38ppCD57pppCX3CR1pCD95pp	CD31+ CD38+ CD57+ CD8- CD16+ CD56dim NK	CD8- CD16+ CD56dim NK cell	CD8- CD16+ CD56dim NK cell	CD56dim NK cell
177	c14	nk	CD45ppCD56ppCD16pCD45RApCD31pSLAMF7pCD38ppCD57pppCX3CR1pCD95p	CD31+ CD38+ CD57+ CD8- CD16+ CD56dim NK	CD8- CD16+ CD56dim NK cell	CD8- CD16+ CD56dim NK cell	CD56dim NK cell
178	c15	nk	CD45ppCD56ppCD16pCD45RAppCD31pSLAMF7pCD38ppCD57ppCX3CR1pCD95p	CD31+ CD38+ CD57+ CD8- CD16+ CD56dim NK	CD8- CD16+ CD56dim NK cell	CD8- CD16+ CD56dim NK cell	CD56dim NK cell
179	c16	nk	CD45ppCD56pppCD45RApCD31pCXCR3pSLAMF7pCD38ppCD95pp	CD38+ CD16- CD56bright NK cell	CD16- CD56bright NK cell	CD16- CD56bright NK cell	CD56bright NK cell
180	c17	nk	CD45ppCD56ppCD16pCD45RAppCD31ppSLAMF7pCD38ppCX3CR1ppCD95p	CD31+ CD38+ CD8- CD16+ CD56dim NK cell	CD8- CD16+ CD56dim NK cell	CD8- CD16+ CD56dim NK cell	CD56dim NK cell
181	c18	nk	CD45ppCD56pppCD16pCD45RAppCD31ppCXCR3pSLAMF7pCD38pCX3CR1pCD95p	CD31+ CD8- CD16+ CD56dim NK cell	CD8- CD16+ CD56dim NK cell	CD8- CD16+ CD56dim NK cell	CD56dim NK cell
182	c19	nk	CD45ppCD56pppCD8ppCD45RApCD31ppCXCR3pCD38ppCD95pCD27p	CD31+ CD38+ CD8+ CD16- CD56bright NK cell	CD8+ CD16- CD56bright NK cell	CD8+ CD16- CD56bright NK ce	CD56bright NK cell
183	c20	nk	CD45ppCD56pppCD45RApCD31ppCXCR3ppSLAMF7pCD38ppCD95pCD27pp	CD31+ CD38+ CD16- CD56bright NK cell	CD16- CD56bright NK cell	CD16- CD56bright NK cell	CD56bright NK cell
184	c21	nk	CD45ppCD56pCD16pCD45RAppCD31pSLAMF7pCD38ppCD57pppCX3CR1pCD95pp	CD57+ CD31+ CD38+ CD8- CD16+ CD56dim NK	CD8- CD16+ CD56dim NK cell	CD8- CD16+ CD56dim NK cell	CD56dim NK cell
185	c22	nk	CD45ppCD56ppCD16pCD45RAppSLAMF7pCD38ppCD57pppCX3CR1pCD95p	CD38+ CD57+ CD8- CD16+ CD56dim NK cell	CD8- CD16+ CD56dim NK cell	CD8- CD16+ CD56dim NK cell	CD56dim NK cell
186	c23	nk	CD45pCD56ppCD16pCD45RAppCD31pSLAMF7pCD38ppCD57pppCX3CR1pCD95p	CD57+ CD31+ CD38+ CD8- CD16+ CD56dim NK	CD8- CD16+ CD56dim NK cell	CD8- CD16+ CD56dim NK cell	CD56dim NK cell
187	c24	nk	CD45ppCD56ppCD16pCD45RAppSLAMF7pCD38ppCD57pppCX3CR1ppCD95p	CD38+ CD57+ CD8- CD16+ CD56dim NK cell	CD8- CD16+ CD56dim NK cell	CD8- CD16+ CD56dim NK cell	CD56dim NK cell
188	c25	nk	CD45ppCD56ppCD16pCD45RAppCD31pSLAMF7pCD38pppCD57ppTIM.3pCX3CR1ppCD95pp	CD57+ CD31+ CD38+ TIM3+ CD8- CD16+ CD56	CD8- CD16+ CD56dim NK cell	CD8- CD16+ CD56dim NK cell	CD56dim NK cell
189	c26	nk	CD45ppCD56ppCD16pCD45RAppCD31pSLAMF7pCD38pppTIM.3pCX3CR1ppCD95pp	CD31+ CD38+ TIM3+ CD8- CD16+ CD56dim NK	CD8- CD16+ CD56dim NK cell	CD8- CD16+ CD56dim NK cell	CD56dim NK cell
190	c27	nk	CD45ppCD56ppCD45RAppCD31ppCXCR3pSLAMF7pCD38ppCD95ppCD69ppCD27pp	CD31+ CD38+ CD16- CD56bright NK cell	CD16- CD56bright NK cell	CD16- CD56bright NK cell	CD56bright NK cell
191	c28	nk	CD45ppCD56ppCD16pCD45RAppCD31ppSLAMF7pCD38pppTIM.3pCX3CR1pCD95p	CD31+ CD38+ TIM3+ CD8- CD16+ CD56dim NK	CD8- CD16+ CD56dim NK cell	CD8- CD16+ CD56dim NK cell	CD56dim NK cell
192	c29	nk	CD45ppCD56pppCD25ppCD127pCD45RApCD31ppCXCR3ppCD38ppCD95pp	CD25+ CD31+ CD38+ CD16- CD56bright NK ce	activated CD16- CD56bright NK cel	CD16- CD56bright NK cell	CD56bright NK cell
193	c30	nk	CD45ppCD56pppCD127pCD45RApCD31pCXCR3pCD38pCD95p	CD16- CD56bright NK cell	CD16- CD56bright NK cell	CD16- CD56bright NK cell	CD56bright NK cell
194	c31	nk	CD45ppCD56ppCD16pCD45RAppCD31ppSLAMF7pCD38ppCD57pppCX3CR1pCD95p	CD57+ CD31+ CD38+ TIM3+ CD8- CD16+ CD56	CD8- CD16+ CD56dim NK cell	CD8- CD16+ CD56dim NK cell	CD56dim NK cell
195	c32	nk	CD45ppCD56ppCD16pCD45RAppCD31ppSLAMF7pCD38pppCD57pppTIM.3pCX3CR1ppCD95p	CD57+ CD31+ CD38+ TIM3+ CD8- CD16+ CD56	CD8- CD16+ CD56dim NK cell	CD8- CD16+ CD56dim NK cell	CD56dim NK cell
196	c33	nk	CD45ppCD56ppCD16pCD45RAppCD31pSLAMF7pCD38ppCD57pppTIM.3pCX3CR1pCD95ppCD69pp	CD57+ CD31+ CD38+ TIM3+ CD8- CD16+ CD56	CD8- CD16+ CD56dim NK cell	CD8- CD16+ CD56dim NK cell	CD56dim NK cell
197	c34	nk	CD45ppCD56ppCD16pCD45RAppCD31ppSLAMF7pCD38pppCD57ppTIM.3pCX3CR1ppCD95pp	CD57+ CD31+ CD38+ TIM3+ CD8- CD16+ CD56	CD8- CD16+ CD56dim NK cell	CD8- CD16+ CD56dim NK cell	CD56dim NK cell
198	c35	nk	CD45ppCD56ppCD16pCD45RAppCD31ppSLAMF7pCD38pppTIM.3pCX3CR1pCD95ppCD69pp	CD31+ CD38+ TIM3+ CD8- CD16+ CD56dim NK	CD8- CD16+ CD56dim NK cell	CD8- CD16+ CD56dim NK cell	CD56dim NK cell
199	c36	nk	CD45ppCD56ppCD16pCD45RAppCD31ppSLAMF7pCD38pppTIM.3pCX3CR1ppCD95pp	CD31+ CD38+ TIM3+ CD8- CD16+ CD56dim NK	CD8- CD16+ CD56dim NK cell	CD8- CD16+ CD56dim NK cell	CD56dim NK cell
200	c37	nk	CD45ppCD56pppCD45RApCD31ppCXCR3pCD38ppCD95p	CD31+ CD38+ CD16- CD56bright NK cell	CD16- CD56bright NK cell	CD16- CD56bright NK cell	CD56bright NK cell
201	c38	nk	CD45ppCD3pCD56ppCD4ppCD14ppCD45RApCD31pppCD38ppTIM.3pCD95pppSlanpCD141pp	Trash!? CD4+ NKT	Trash!? CD4+ NKT	Trash!? CD4+ NKT	Trash!? CD4+ NKT
202	c39	nk	CD45ppCD56pCD16pCD45RAppCD31ppSLAMF7pCD38ppCD57pppCX3CR1pCD95p	CD57+ CD31+ CD38+ CD8- CD16+ CD56dim NK	CD8- CD16+ CD56dim NK cell	CD8- CD16+ CD56dim NK cell	CD56dim NK cell
203	c40	nk	CD45ppCD56ppCD16pCD45RAppCD31ppSLAMF7pCD38ppCD57pppTIM.3pCX3CR1pCD95pCD69pp	CD57+ CD31+ CD38+ TIM3+ CD8- CD16+ CD56	CD8- CD16+ CD56dim NK cell	CD8- CD16+ CD56dim NK cell	CD56dim NK cell
204	c41	nk	CD45ppCD56ppCD16pCD45RAppCD31ppSLAMF7pCD38ppCD57pppCX3CR1pCD95pp	CD57+ CD31+ CD38+ CD8- CD16+ CD56dim NK	CD8- CD16+ CD56dim NK cell	CD8- CD16+ CD56dim NK cell	CD56dim NK cell
205	c42	nk	CD45ppCD56pCD16pCD45RAppCD31ppSLAMF7pCD38ppCD57ppTIM.3pCX3CR1ppCD95p	CD57+ CD31+ CD38+ TIM3+ CD8- CD16+ CD56	CD8- CD16+ CD56dim NK cell	CD8- CD16+ CD56dim NK cell	CD56dim NK cell
206	c43	nk	CD45ppCD56pCD16pCD45RAppCD31ppSLAMF7pCD38pppTIM.3pCX3CR1ppCD95pp	CD31+ CD38+ TIM3+ CD8- CD16+ CD56dim NK	CD8- CD16+ CD56dim NK cell	CD8- CD16+ CD56dim NK cell	CD56dim NK cell
207	c44	nk	CD45ppCD56pCD16pCD45RAppCD31ppSLAMF7pCD38ppCX3CR1pCD95p	CD31+ CD38+ CD8- CD16+ CD56dim NK cell	CD8- CD16+ CD56dim NK cell	CD8- CD16+ CD56dim NK cell	CD56dim NK cell
208	c45	nk	CD45ppCD56ppCD16pCD45RAppCD31ppSLAMF7pCD38pCD57pppCX3CR1pCD95pp	CD57+ CD31+ CD8- CD16+ CD56dim NK cell	CD8- CD16+ CD56dim NK cell	CD8- CD16+ CD56dim NK cell	CD56dim NK cell
209	c46	nk	CD45ppCD56ppCD16pCD45RAppCD31ppSLAMF7pCD38ppCD57ppTIM.3pCX3CR1pCD95p	CD57+ CD31+ CD38+ TIM3+ CD8- CD16+ CD56	CD8- CD16+ CD56dim NK cell	CD8- CD16+ CD56dim NK cell	CD56dim NK cell
210	c47	nk	CD45pppCD56ppCD4pCD8pCD16pCD45RAppCD31pppSLAMF7pCD38ppCD57pppTIM.3pCX3CR1ppCD95ppSlanpppCD141pp	CD16+ Trash!? Monocyte	CD16+ Trash!? Monocyte	CD16+ Trash!? Monocyte	CD16+ Trash!? Monoc
211	c48	nk	CD45pppCD3pCD56ppCD4ppCD14ppCD16pCD45RAppCD31pppSLAMF7pCD38pppCD57pppTIM.3pCX3CR1ppCD95pppSlanpCD141p	CD16+ Trash!? CD4+ NKT	CD16+ Trash!? CD4+ NKT	CD16+ Trash!? CD4+ NKT	CD16+ Trash!? CD4+ N
212	c49	nk	CD45ppCD3pCD4ppCD14ppCD31pppIL6RpCD38ppTIM.3ppCX3CR1ppCD95ppCD141pp	Trash!? CD4+ Tcell	Trash!? CD4+ Tcell	Trash!? CD4+ Tcell	Trash!? CD4+ Tcell
213	c50	nk	CD45ppCD4ppCD14ppCD31pppCD38ppTIM.3ppCX3CR1pCD95ppCD141pp	Trash!? Monocyte	Trash!? Monocyte	Trash!? Monocyte	Trash!? Monocyte
214	c51	nk	CD45ppCD56ppCD8pCD16pCD45RAppCD31ppSLAMF7pCD38pCD57ppCX3CR1pCD95p	CD57+ CD31+ CD8+ CD16+ CD56dim NK cell	CD8+ CD16+ CD56dim NK cell	CD8- CD16+ CD56dim NK cell	CD56dim NK cell
215	c52	nk	CD45ppCD56ppCD8ppCD16pCD45RAppCD31ppSLAMF7pCD38ppCD57ppTIM.3pCX3CR1pCD95p	CD57+ CD31+ CD38+ TIM3+ CD8+ CD16+ CD56	CD8+ CD16+ CD56dim NK cell	CD8+ CD16+ CD56dim NK cell	CD56dim NK cell
216	c53	nk	CD45ppCD56ppCD8ppCD16pCD45RAppCD31pSLAMF7pCD38ppCD57ppCX3CR1pCD95p	CD57+ CD31+ CD38+ CD8+ CD16+ CD56dim N	CD8+ CD16+ CD56dim NK cell	CD8+ CD16+ CD56dim NK cell	CD56dim NK cell
217	c54	nk	CD45pppCD56ppCD4pCD8ppCD14ppCD16pCD45RAppCD31pppSLAMF7pCD38pppCD57pppTIM.3pCX3CR1ppCD95ppSlanpCD141p	CD16+ Trash!? Monocyte	CD16+ Trash!? Monocyte	CD16+ Trash!? Monocyte	CD16+ Trash!? Monoc
218	c55	nk	CD45pppCD3pppCD56pCD4ppCD8pppCD14ppCD45RAppCD31pppSLAMF7pCD38ppCD57pppTIM.3pCX3CR1pCD95pppSlanpCD141pp	Trash!? Monocyte	Trash!? Monocyte	Trash!? Monocyte	Trash!? Monocyte
219	c56	nk	CD45ppCD3pCD4ppCD14ppCD45RAppCD31pppIL6RpSLAMF7pCD38ppTIM.3ppCX3CR1ppCD95ppSlanppCD141pp	Trash!? Monocyte	Trash!? Monocyte	Trash!? Monocyte	Trash!? Monocyte
220	c57	nk	CD45ppCD3pCD4ppCD14ppCD45RApCD31pppIL6RpCD38ppTIM.3ppCX3CR1ppCD95ppSlanppCD141pp	Trash!? Monocyte	Trash!? Monocyte	Trash!? Monocyte	Trash!? Monocyte
221	c58	nk	CD45ppCD3pCD4ppCD14ppCD45RApCD31pppCD38ppTIM.3ppCX3CR1pCD95pppSlanppCD141p	Trash!? Monocyte	Trash!? Monocyte	Trash!? Monocyte	Trash!? Monocyte
222	c59	nk	CD45ppCD3pCD4ppCD14ppCD45RApCD31pppCD38ppTIM.3ppCX3CR1pCD95ppSlanppCD141pp	Trash!? Monocyte	Trash!? Monocyte	Trash!? Monocyte	Trash!? Monocyte
223	c60	nk	CD45ppCD3pCD56ppCD4ppCD14ppCD45RApCD31pppCD38ppTIM.3ppCD95pppSlanppCD141pp	Trash!? CD4+ NKT	Trash!? CD4+ NKT	Trash!? CD4+ NKT	Trash!? CD4+ NKT
224	c61	nk	CD45ppCD56ppCD8ppCD16pCD45RAppCD31ppSLAMF7pCD38pCX3CR1pCD95p	CD31+ CD8+ CD16+ CD56dim NK cell	CD8+ CD16+ CD56dim NK cell	CD8+ CD16+ CD56dim NK cell	CD56dim NK cell
225	c62	nk	CD45ppCD56ppCD8pppCD16pCD45RAppCD31ppSLAMF7pCD38pppTIM.3pCX3CR1ppCD95pp	CD31+ CD38+ TIM3+ CD8+ CD16+ CD56dim N	CD8+ CD16+ CD56dim NK cell	CD8+ CD16+ CD56dim NK cell	CD56dim NK cell
226	c63	nk	CD45ppCD56pCD8ppCD16pCD45RAppCD31ppSLAMF7pCD38pppTIM.3pCX3CR1ppCD95p	CD31+ CD38+ TIM3+ CD8+ CD16+ CD56dim N	CD8+ CD16+ CD56dim NK cell	CD8+ CD16+ CD56dim NK cell	CD56dim NK cell
227	c64	nk	CD45pppCD3pCD56ppCD4ppCD8pCD14ppCD16pCD45RAppCD31pppSLAMF7pCD38pppTIM.3ppCX3CR1ppCD95pppCD69pCD141p	CD16+ Trash!? CD4+ NKT	CD16+ Trash!? CD4+ NKT	CD16+ Trash!? CD4+ NKT	CD16+ Trash!? CD4+ N
228	c65	nk	CD45ppCD3pCD4ppCD14ppCD31pppCD38ppTIM.3ppCX3CR1ppCD95ppCD141pp	Trash!? CD4+ Tcell	Trash!? CD4+ Tcell	Trash!? CD4+ Tcell	Trash!? CD4+ Tcell
229	c66	nk	CD45ppCD3pCD4ppCD14ppCD45RAppCD31pppIL6RpCD38ppTIM.3ppCX3CR1ppCD95ppSlanpCD141pp	Trash!? Monocyte	Trash!? Monocyte	Trash!? Monocyte	Trash!? Monocyte
230	c67	nk	CD45ppCD3pCD4ppCD14ppCD45RApCD31pppIL6RpCD38ppTIM.3ppCX3CR1pCD95ppSlanppCD141pp	Trash!? Monocyte	Trash!? Monocyte	Trash!? Monocyte	Trash!? Monocyte
231	c68	nk	CD45ppCD56ppCD8ppCD45RAppCD31pCXCR3pSLAMF7pCD38pCX3CR1pCD95p	CD31+ CD38+ CD8+ CD16+ CD56dim NK cell	CD8+ CD16+ CD56dim NK cell	CD8+ CD16+ CD56dim NK cell	CD56dim NK cell
232	c69	nk	CD45ppCD56ppCD8pppCD16pCD45RAppCD31ppSLAMF7pCD38ppCX3CR1pCD95p	CD31+ CD38+ CD8+ CD16+ CD56dim NK cell	CD8+ CD16+ CD56dim NK cell	CD8+ CD16+ CD56dim NK cell	CD56dim NK cell
233	c70	nk	CD45ppCD56ppCD8ppCD16pCD45RAppCD31pSLAMF7pCD38pppTIM.3pCX3CR1ppCD95p	CD31+ CD38+ TIM3+ CD8+ CD16+ CD56dim N	CD8+ CD16+ CD56dim NK cell	CD8+ CD16+ CD56dim NK cell	CD56dim NK cell
234	c71	nk	CD45ppCD56ppCD8ppCD16pCD45RAppCD31ppSLAMF7pCD38pppTIM.3pCX3CR1ppCD95p	CD31+ CD38+ TIM3+ CD8+ CD16+ CD56dim N	CD8+ CD16+ CD56dim NK cell	CD8+ CD16+ CD56dim NK cell	CD56dim NK cell
235	c72	nk	CD45ppCD3pCD4ppCD14ppCD16pCD45RApCD31pppSLAMF7pCD38ppTIM.3ppCX3CR1ppCD95ppCD141pp	Trash!? NK cell	Trash!? NK cell	Trash!? NK cell	Trash!? NK cell
236	c73	nk	CD45ppCD3pCD4ppCD14ppCD31pppCCR4pIL6RpCD38ppTIM.3pCX3CR1pCD95pppCD69pCD141pp	Trash!? CD4+ Tcell	Trash!? CD4+ Tcell	Trash!? CD4+ Tcell	Trash!? CD4+ Tcell
237	c74	nk	CD45ppCD3pCD4ppCD14ppCD45RApCD31pppIL6RpSLAMF7ppCD38ppTIM.3ppCX3CR1pCD95ppSlanpCD141pp	Trash!? Monocyte	Trash!? Monocyte	Trash!? Monocyte	Trash!? Monocyte
238	c75	nk	CD45ppCD56ppCD8pppCD45RAppCXCR3ppSLAMF7pCD95pp	CD8+ CD16- CD56bright NK cell	CD8+ CD16- CD56bright NK cell	CD8+ CD16- CD56bright NK ce	CD56bright NK cell
239	c76	nk	CD45ppCD56pCD8ppCD16pCD45RApCD31pSLAMF7pCD38ppCX3CR1pCD95p	CD31+ CD38+ CD8+ CD16+ CD56dim NK cell	CD8+ CD16+ CD56dim NK cell	CD8+ CD16+ CD56dim NK cell	CD56dim NK cell
240	c77	nk	CD45ppCD56ppCD8pppCD16pCD45RAppSLAMF7pCD38ppCX3CR1pCD95p	CD38+ CD8+ CD16+ CD56dim NK cell	CD8+ CD16+ CD56dim NK cell	CD8+ CD16+ CD56dim NK cell	CD56dim NK cell
241	c78	nk	CD45ppCD3pCD56pCD4pCD8pCD14pCD45RApCD31pppCD38ppTIM.3pCX3CR1ppCD95ppCD141p	Trash!? CD4+ NKT	Trash!? CD4+ NKT	Trash!? CD4+ NKT	Trash!? CD4+ NKT
242	c79	nk	CD45ppCD3pCD4ppCD14ppCD45RApCD31pppCCR4ppCD38ppTIM.3pCD95ppCD69ppSlanpCD141pp	Trash!? Monocyte	Trash!? Monocyte	Trash!? Monocyte	Trash!? Monocyte
243	c80	nk	CD45ppCD3pCD4ppCD14ppCD45RApCD31pppCCR4pIL6RpCD38ppTIM.3ppCX3CR1pCD95pppCD69ppSlanppCD141pp	Trash!? Monocyte	Trash!? Monocyte	Trash!? Monocyte	Trash!? Monocyte

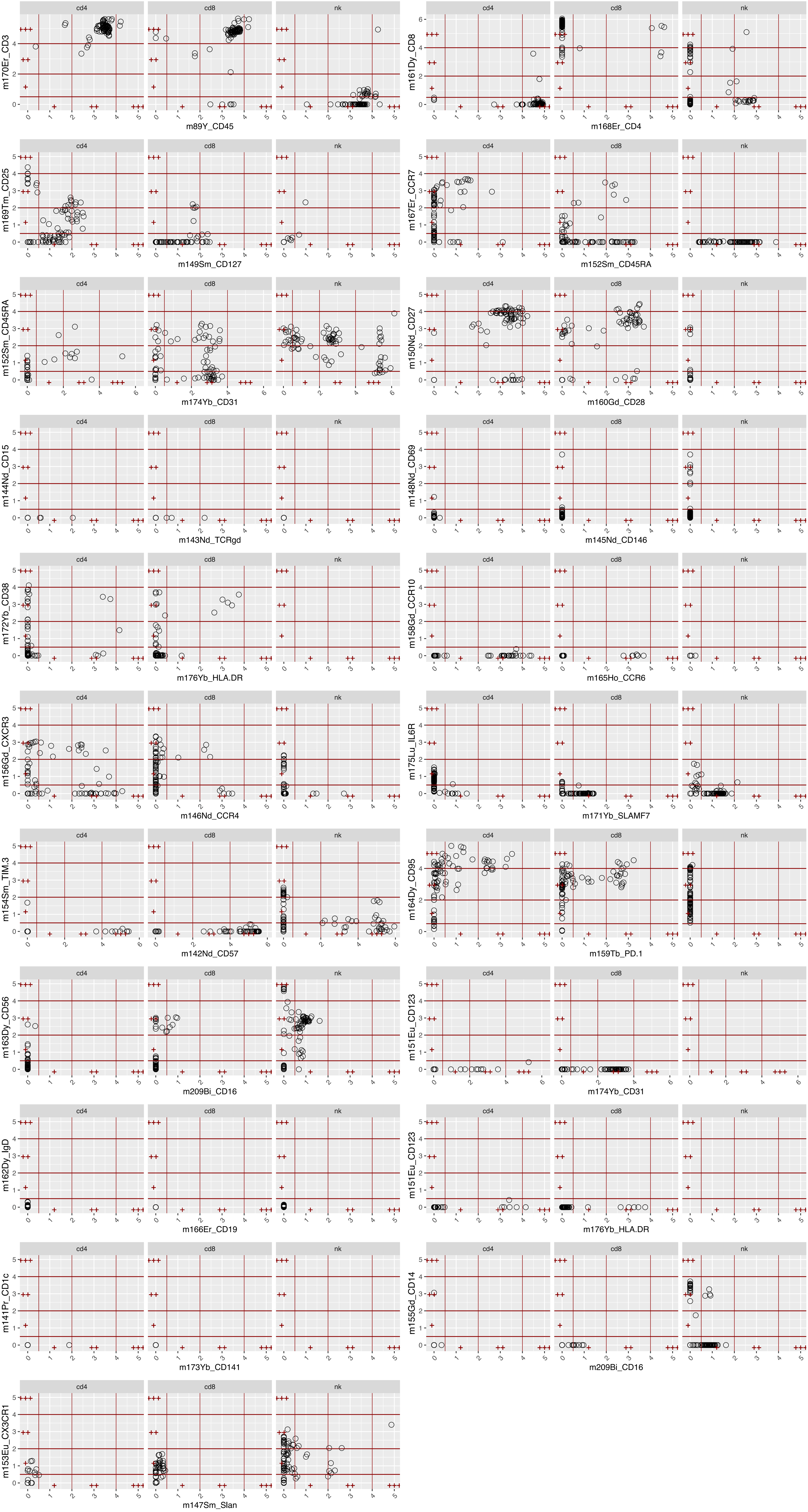
cd4
cd8
nk
m170Er_CD3
m89Y_CD45
m161Dy_CD8
m168Er_CD4
m169Tm_CD25
m149Sm_CD127
m167Er_CCR7
m152Sm_CD45RA
m152Sm_CD45RA
m174Yb_CD31
m150Nd_CD27
m160Gd_CD28
m144Nd_CD15
m143Nd_TCRgd
m148Nd_CD69
m145Nd_CD146
m172Yb_CD38
m176Yb_HLA.DR
m158Gd_CCR10
m165Ho_CCR6
m156Gd_CXCR3
m146Nd_CCR4
m175Lu_IL6R
m171Yb_SLAMF7
m154Sm_TIM.3
m142Nd_CD57
m164Dy_CD95
m159Tb_PD.1
m163Dy_CD56
m209Bi_CD16
m151Eu_CD123
m174Yb_CD31
m162Dy_IgD
m166Er_CD19
m151Eu_CD123
m176Yb_HLA.DR
m141Pr_CD1c
m173Yb_CD141
m155Gd_CD14
m209Bi_CD16
m153Eu_CX3CR1
m147Sm_Slan

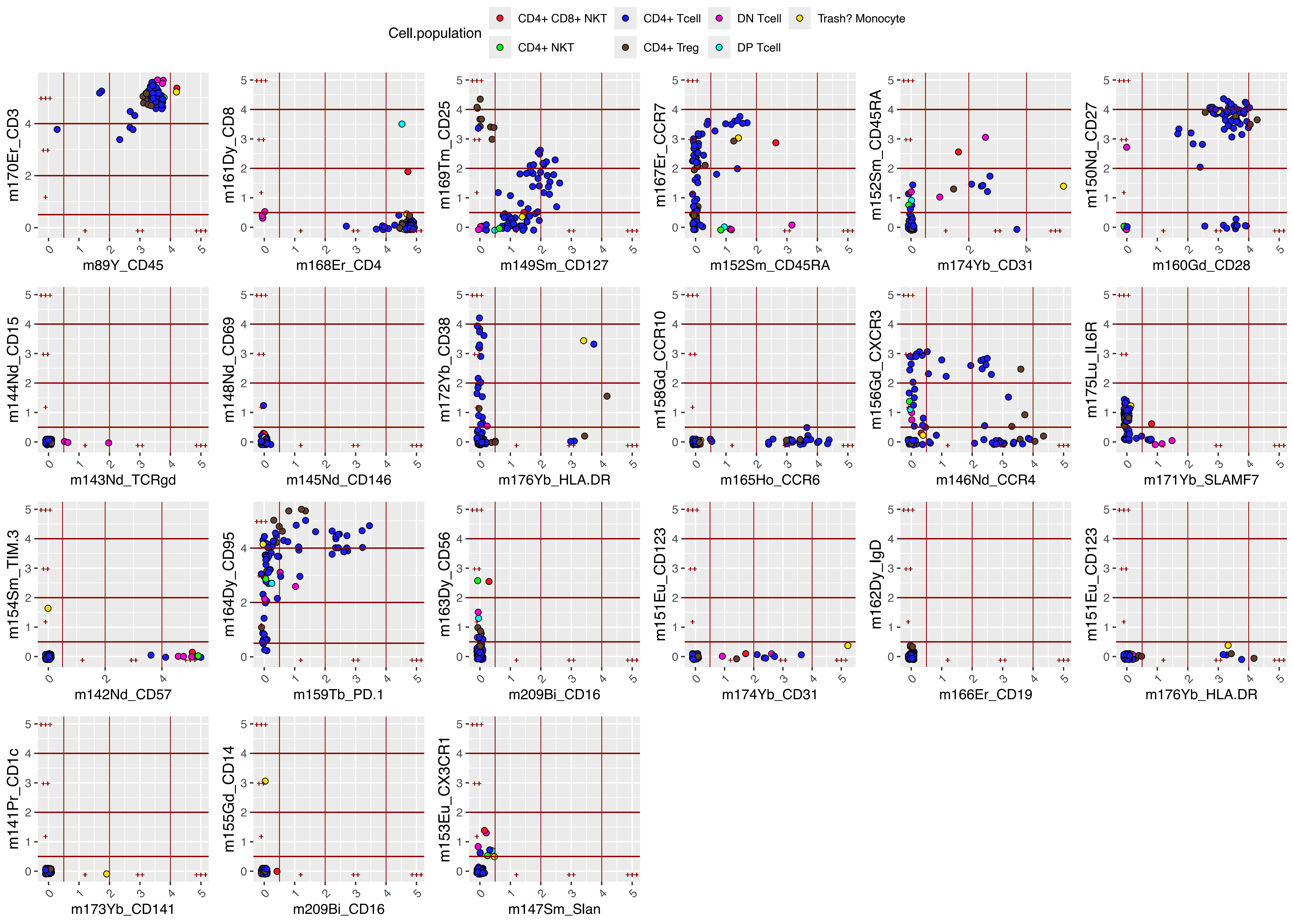

Cell.population
CD4+ CD8+ NKT
CD4+ Tcell
DN Tcell
Trash? Monocyte
CD4+ NKT
CD4+ Treg
DP Tcell
m170Er_CD3
m89Y_CD45
m161Dy_CD8
m168Er_CD4
m169Tm_CD25
m149Sm_CD127
m167Er_CCR7
m152Sm_CD45RA
m152Sm_CD45RA
m174Yb_CD31
m150Nd_CD27
m160Gd_CD28
m144Nd_CD15
m143Nd_TCRgd
m148Nd_CD69
m145Nd_CD146
m172Yb_CD38
m176Yb_HLA.DR
m158Gd_CCR10
m165Ho_CCR6
m156Gd_CXCR3
m146Nd_CCR4
m175Lu_IL6R
m171Yb_SLAMF7
m154Sm_TIM.3
m142Nd_CD57
m164Dy_CD95
m159Tb_PD.1
m163Dy_CD56
m209Bi_CD16
m151Eu_CD123
m174Yb_CD31
m162Dy_IgD
m166Er_CD19
m151Eu_CD123
m176Yb_HLA.DR
m141Pr_CD1c
m173Yb_CD141
m155Gd_CD14
m209Bi_CD16
m153Eu_CX3CR1
m147Sm_Slan

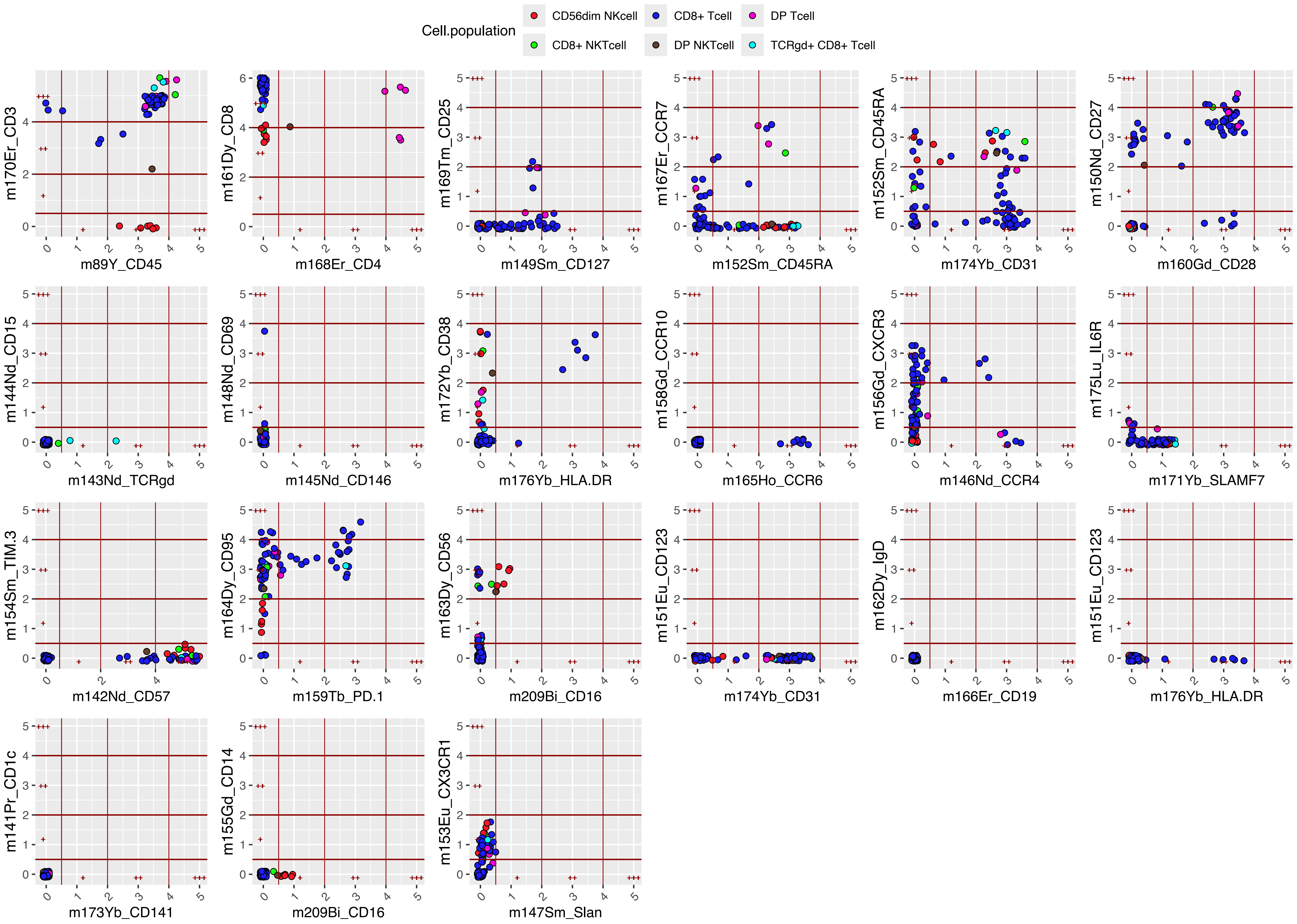
Cell.population
CD56dim NKcell
CD8+ Tcell
DP Tcell
CD8+ NKTcell
DP NKTcell
TCRgd+ CD8+ Tcell
m170Er_CD3
m89Y_CD45
m161Dy_CD8
m168Er_CD4
m169Tm_CD25
m149Sm_CD127
m167Er_CCR7
m152Sm_CD45RA
m152Sm_CD45RA
m174Yb_CD31
m150Nd_CD27
m160Gd_CD28
m144Nd_CD15
m143Nd_TCRgd
m148Nd_CD69
m145Nd_CD146
m172Yb_CD38
m176Yb_HLA.DR
m158Gd_CCR10
m165Ho_CCR6
m156Gd_CXCR3
m146Nd_CCR4
m175Lu_IL6R
m171Yb_SLAMF7
m154Sm_TIM.3
m142Nd_CD57
m164Dy_CD95
m159Tb_PD.1
m163Dy_CD56
m209Bi_CD16
m151Eu_CD123
m174Yb_CD31
m162Dy_IgD
m166Er_CD19
m151Eu_CD123
m176Yb_HLA.DR
m141Pr_CD1c
m173Yb_CD141
m155Gd_CD14
m209Bi_CD16
m153Eu_CX3CR1
m147Sm_Slan

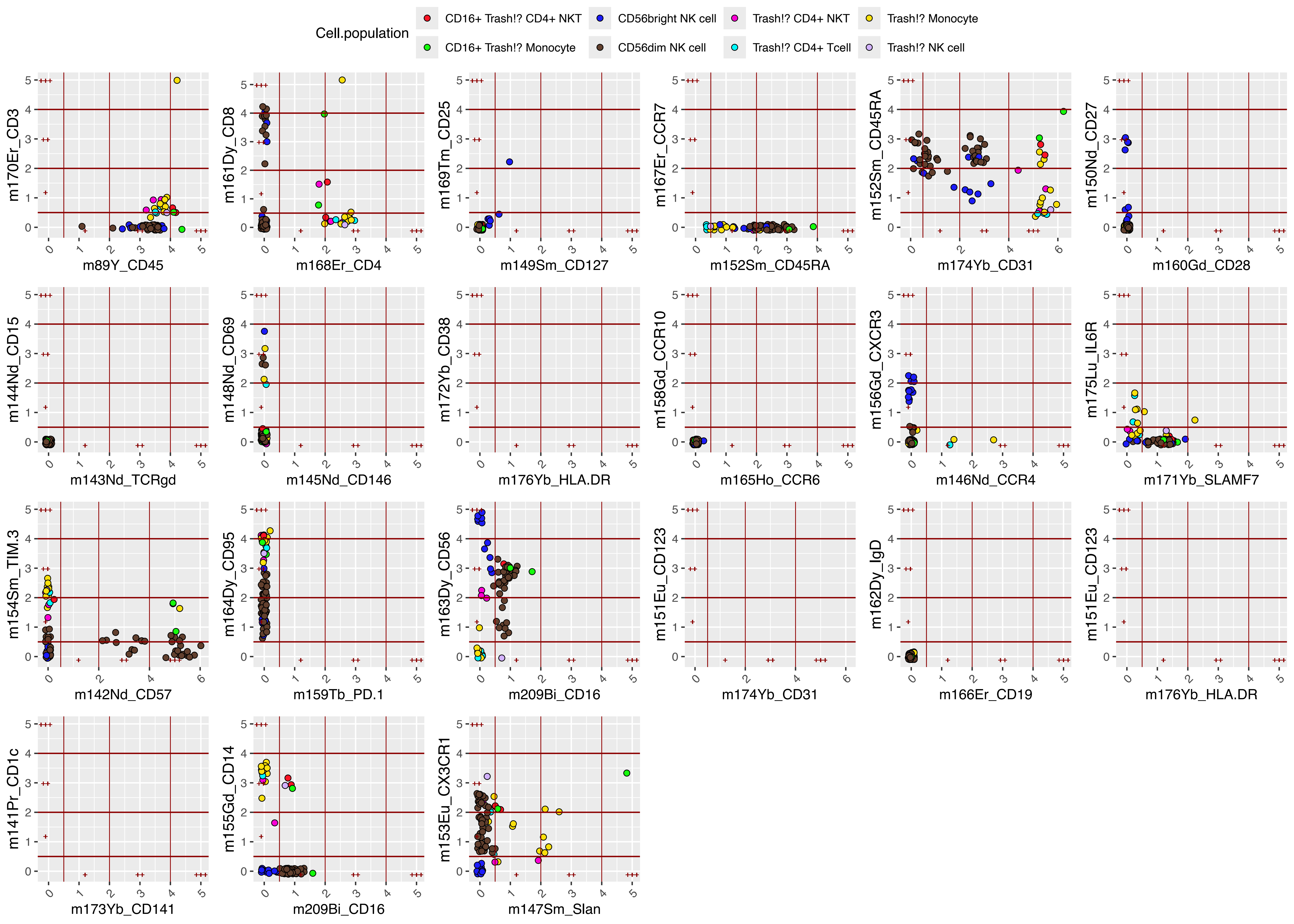

Cell.population
CD16+ Trash!? CD4+ NKT
CD56bright NK cell
Trash!? CD4+ NKT
Trash!? Monocyte
CD16+ Trash!? Monocyte
CD56dim NK cell
Trash!? CD4+ Tcell
Trash!? NK cell
m170Er_CD3
m89Y_CD45
m161Dy_CD8
m168Er_CD4
m169Tm_CD25
m149Sm_CD127
m167Er_CCR7
m152Sm_CD45RA
m152Sm_CD45RA
m174Yb_CD31
m150Nd_CD27
m160Gd_CD28
m144Nd_CD15
m143Nd_TCRgd
m148Nd_CD69
m145Nd_CD146
m172Yb_CD38
m176Yb_HLA.DR
m158Gd_CCR10
m165Ho_CCR6
m156Gd_CXCR3
m146Nd_CCR4
m175Lu_IL6R
m171Yb_SLAMF7
m154Sm_TIM.3
m142Nd_CD57
m164Dy_CD95
m159Tb_PD.1
m163Dy_CD56
m209Bi_CD16
m151Eu_CD123
m174Yb_CD31
m162Dy_IgD
m166Er_CD19
m151Eu_CD123
m176Yb_HLA.DR
m141Pr_CD1c
m173Yb_CD141
m155Gd_CD14
m209Bi_CD16
m153Eu_CX3CR1
m147Sm_Slan

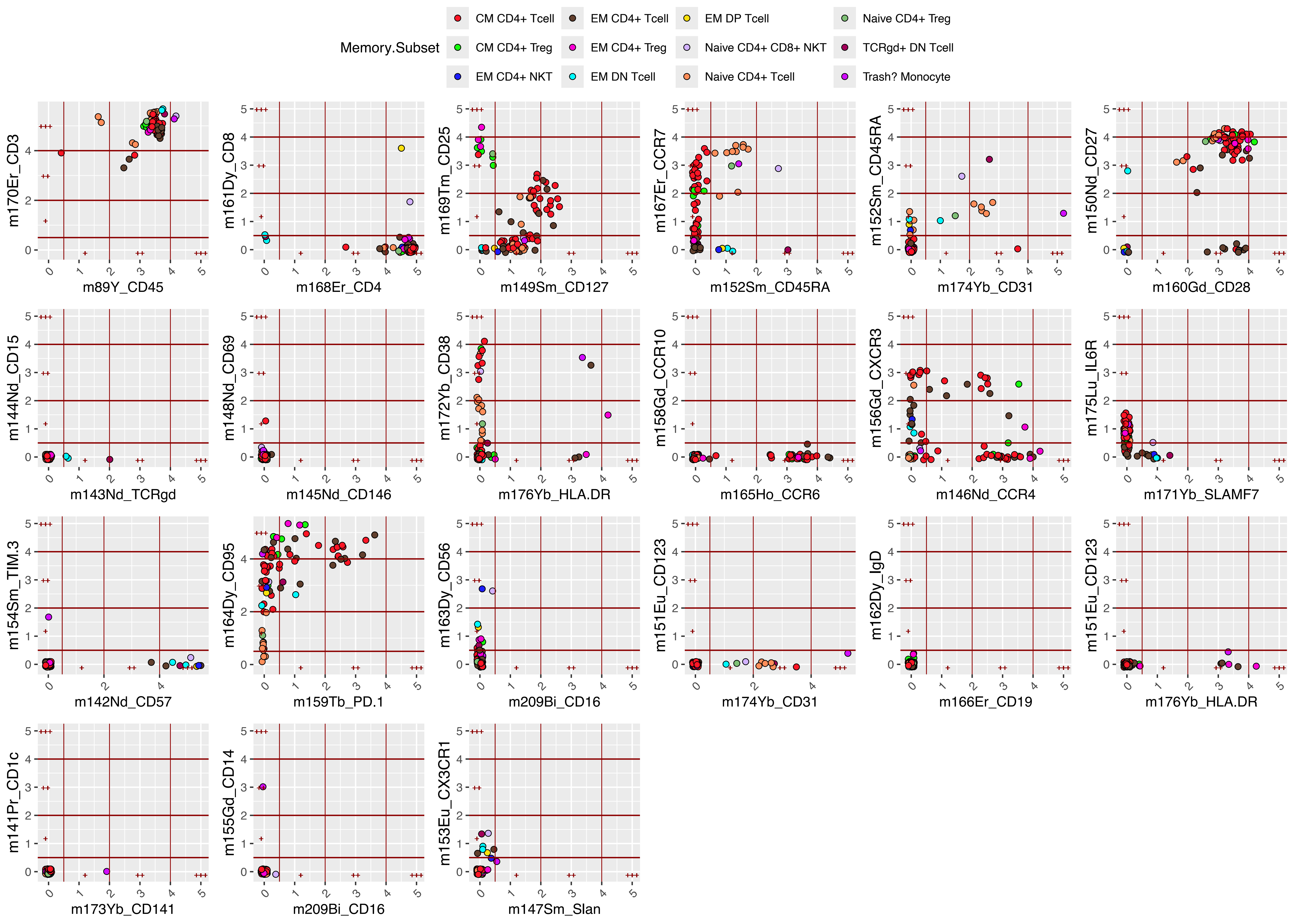

Memory.Subset
CM CD4+ Tcell
EM CD4+ Tcell
EM DP Tcell
Naive CD4+ Treg
CM CD4+ Treg
EM CD4+ Treg
Naive CD4+ CD8+ NKT
TCRgd+ DN Tcell
EM CD4+ NKT
EM DN Tcell
Naive CD4+ Tcell
Trash? Monocyte
m170Er_CD3
m89Y_CD45
m161Dy_CD8
m168Er_CD4
m169Tm_CD25
m149Sm_CD127
m167Er_CCR7
m152Sm_CD45RA
m152Sm_CD45RA
m174Yb_CD31
m150Nd_CD27
m160Gd_CD28
m144Nd_CD15
m143Nd_TCRgd
m148Nd_CD69
m145Nd_CD146
m172Yb_CD38
m176Yb_HLA.DR
m158Gd_CCR10
m165Ho_CCR6
m156Gd_CXCR3
m146Nd_CCR4
m175Lu_IL6R
m171Yb_SLAMF7
m154Sm_TIM.3
m142Nd_CD57
m164Dy_CD95
m159Tb_PD.1
m163Dy_CD56
m209Bi_CD16
m151Eu_CD123
m174Yb_CD31
m162Dy_IgD
m166Er_CD19
m151Eu_CD123
m176Yb_HLA.DR
m141Pr_CD1c
m173Yb_CD141
m155Gd_CD14
m209Bi_CD16
m153Eu_CX3CR1
m147Sm_Slan

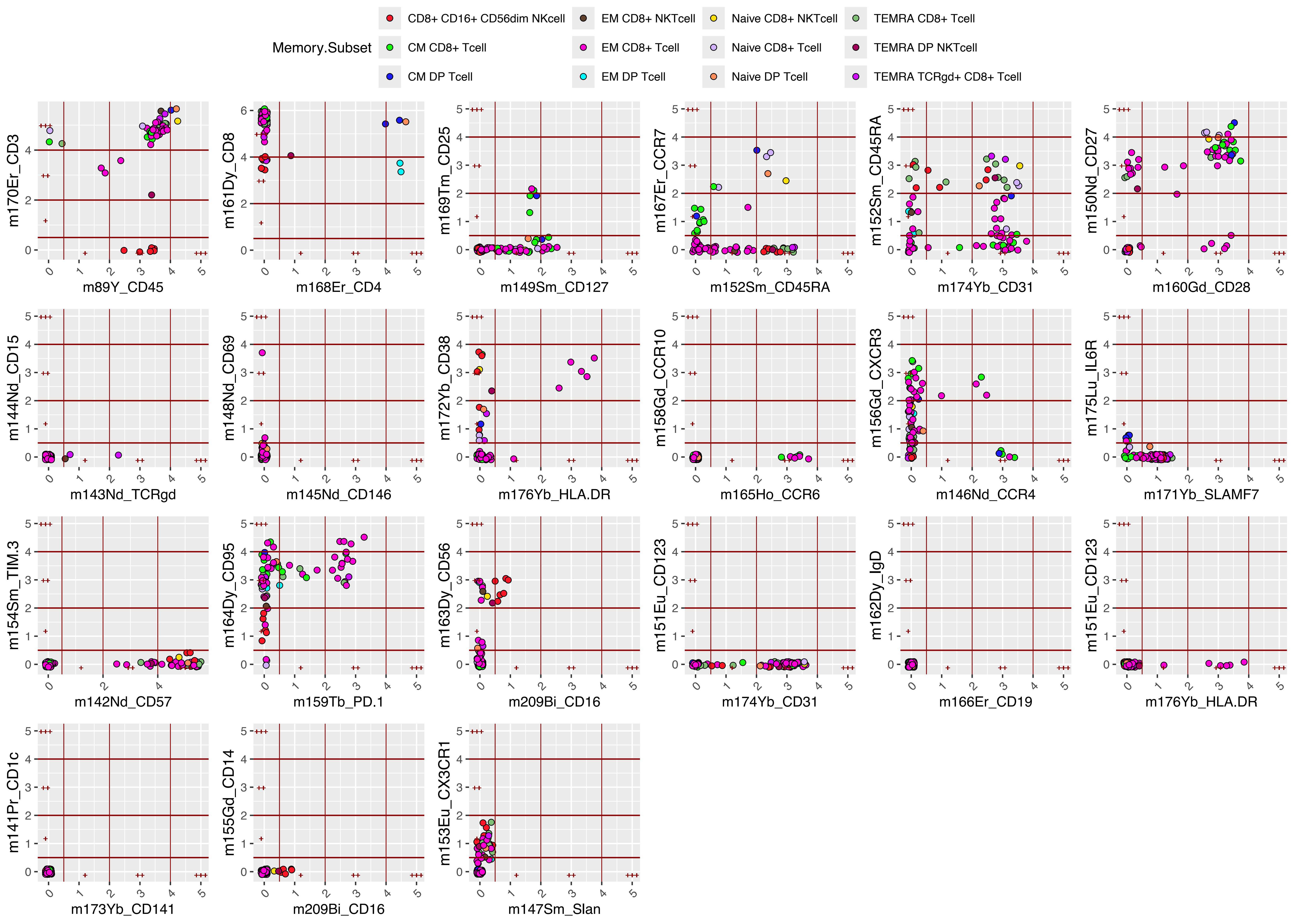

Memory.Subset
CD8+ CD16+ CD56dim NKcell
CM CD8+ Tcell
CM DP Tcell
EM CD8+ NKTcell
EM CD8+ Tcell
EM DP Tcell
Naive CD8+ NKTcell
Naive CD8+ Tcell
Naive DP Tcell
TEMRA CD8+ Tcell
TEMRA DP NKTcell
TEMRA TCRgd+ CD8+ Tcell
m170Er_CD3
m89Y_CD45
m161Dy_CD8
m168Er_CD4
m169Tm_CD25
m149Sm_CD127
m167Er_CCR7
m152Sm_CD45RA
m152Sm_CD45RA
m174Yb_CD31
m150Nd_CD27
m160Gd_CD28
m144Nd_CD15
m143Nd_TCRgd
m148Nd_CD69
m145Nd_CD146
m172Yb_CD38
m176Yb_HLA.DR
m158Gd_CCR10
m165Ho_CCR6
m156Gd_CXCR3
m146Nd_CCR4
m175Lu_IL6R
m171Yb_SLAMF7
m154Sm_TIM.3
m142Nd_CD57
m164Dy_CD95
m159Tb_PD.1
m163Dy_CD56
m209Bi_CD16
m151Eu_CD123
m174Yb_CD31
m162Dy_IgD
m166Er_CD19
m151Eu_CD123
m176Yb_HLA.DR
m141Pr_CD1c
m173Yb_CD141
m155Gd_CD14
m209Bi_CD16
m153Eu_CX3CR1
m147Sm_Slan

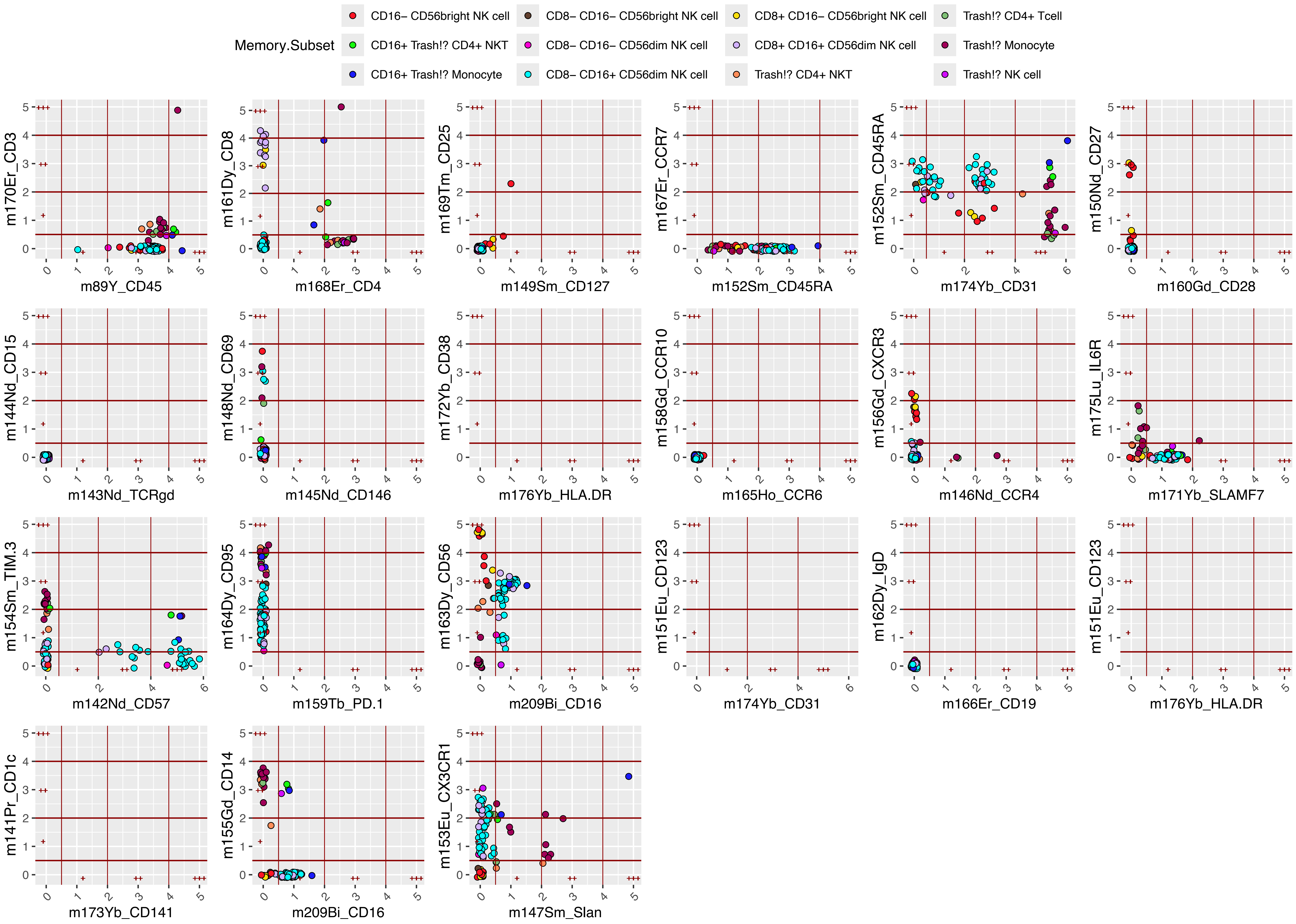
Memory.Subset
CD16− CD56bright NK cell
CD8− CD16− CD56bright NK cell
CD8+ CD16− CD56bright NK cell
Trash!? CD4+ Tcell
CD16+ Trash!? CD4+ NKT
CD8− CD16− CD56dim NK cell
CD8+ CD16+ CD56dim NK cell
Trash!? Monocyte
CD16+ Trash!? Monocyte
CD8− CD16+ CD56dim NK cell
Trash!? CD4+ NKT
Trash!? NK cell
m170Er_CD3
m89Y_CD45
m161Dy_CD8
m168Er_CD4
m169Tm_CD25
m149Sm_CD127
m167Er_CCR7
m152Sm_CD45RA
m152Sm_CD45RA
m174Yb_CD31
m150Nd_CD27
m160Gd_CD28
m144Nd_CD15
m143Nd_TCRgd
m148Nd_CD69
m145Nd_CD146
m172Yb_CD38
m176Yb_HLA.DR
m158Gd_CCR10
m165Ho_CCR6
m156Gd_CXCR3
m146Nd_CCR4
m175Lu_IL6R
m171Yb_SLAMF7
m154Sm_TIM.3
m142Nd_CD57
m164Dy_CD95
m159Tb_PD.1
m163Dy_CD56
m209Bi_CD16
m151Eu_CD123
m174Yb_CD31
m162Dy_IgD
m166Er_CD19
m151Eu_CD123
m176Yb_HLA.DR
m141Pr_CD1c
m173Yb_CD141
m155Gd_CD14
m209Bi_CD16
m153Eu_CX3CR1
m147Sm_Slan

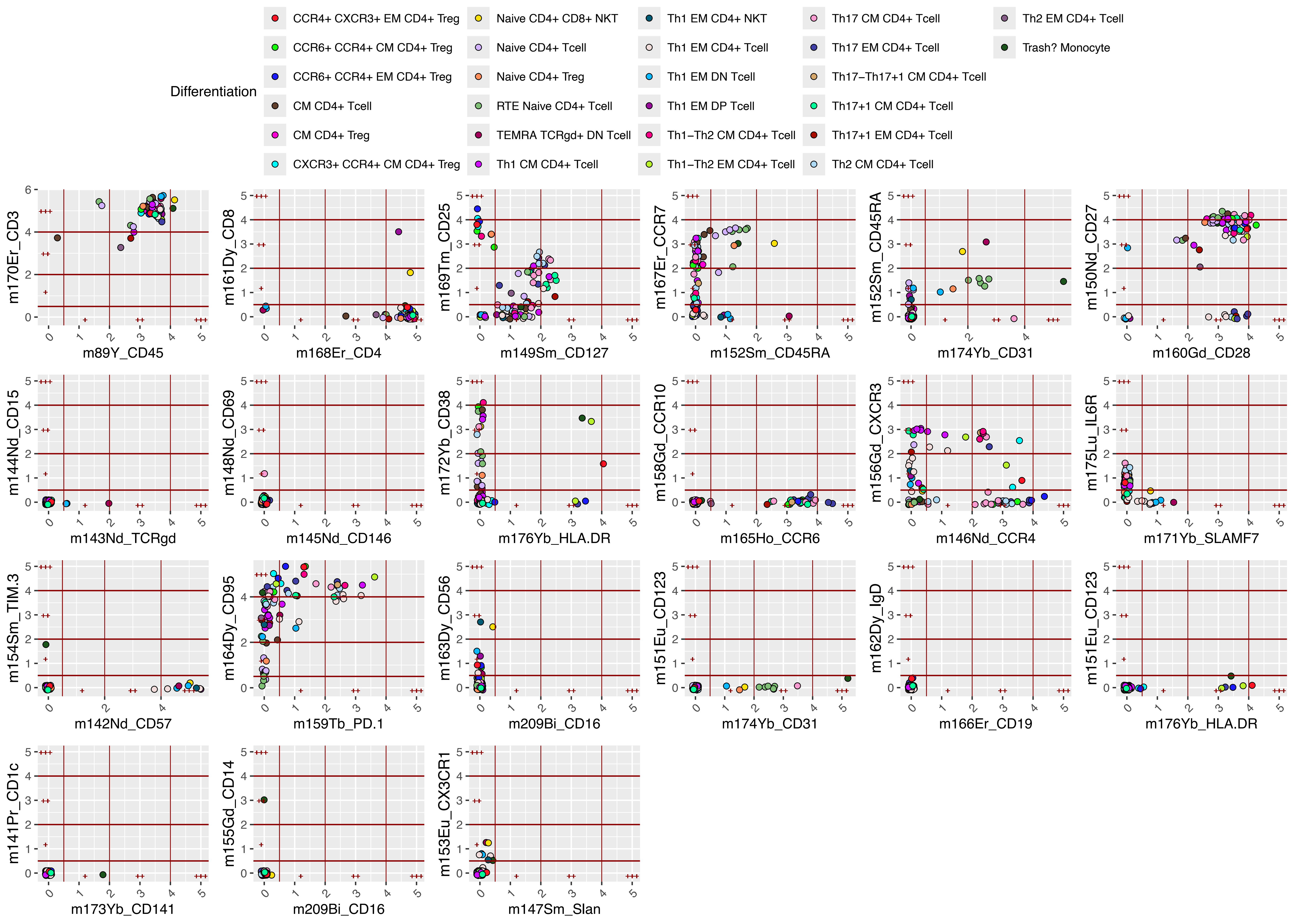
Differentiation
CCR4+ CXCR3+ EM CD4+ Treg
CCR6+ CCR4+ CM CD4+ Treg
CCR6+ CCR4+ EM CD4+ Treg
CM CD4+ Tcell
CM CD4+ Treg
CXCR3+ CCR4+ CM CD4+ Treg
Naive CD4+ CD8+ NKT
Naive CD4+ Tcell
Naive CD4+ Treg
RTE Naive CD4+ Tcell
TEMRA TCRgd+ DN Tcell
Th1 CM CD4+ Tcell
Th1 EM CD4+ NKT
Th1 EM CD4+ Tcell
Th1 EM DN Tcell
Th1 EM DP Tcell
Th1−Th2 CM CD4+ Tcell
Th1−Th2 EM CD4+ Tcell
Th17 CM CD4+ Tcell
Th17 EM CD4+ Tcell
Th17−Th17+1 CM CD4+ Tcell
Th17+1 CM CD4+ Tcell
Th17+1 EM CD4+ Tcell
Th2 CM CD4+ Tcell
Th2 EM CD4+ Tcell
Trash? Monocyte
m170Er_CD3
m89Y_CD45
m161Dy_CD8
m168Er_CD4
m169Tm_CD25
m149Sm_CD127
m167Er_CCR7
m152Sm_CD45RA
m152Sm_CD45RA
m174Yb_CD31
m150Nd_CD27
m160Gd_CD28
m144Nd_CD15
m143Nd_TCRgd
m148Nd_CD69
m145Nd_CD146
m172Yb_CD38
m176Yb_HLA.DR
m158Gd_CCR10
m165Ho_CCR6
m156Gd_CXCR3
m146Nd_CCR4
m175Lu_IL6R
m171Yb_SLAMF7
m154Sm_TIM.3
m142Nd_CD57
m164Dy_CD95
m159Tb_PD.1
m163Dy_CD56
m209Bi_CD16
m151Eu_CD123
m174Yb_CD31
m162Dy_IgD
m166Er_CD19
m151Eu_CD123
m176Yb_HLA.DR
m141Pr_CD1c
m173Yb_CD141
m155Gd_CD14
m209Bi_CD16
m153Eu_CX3CR1
m147Sm_Slan

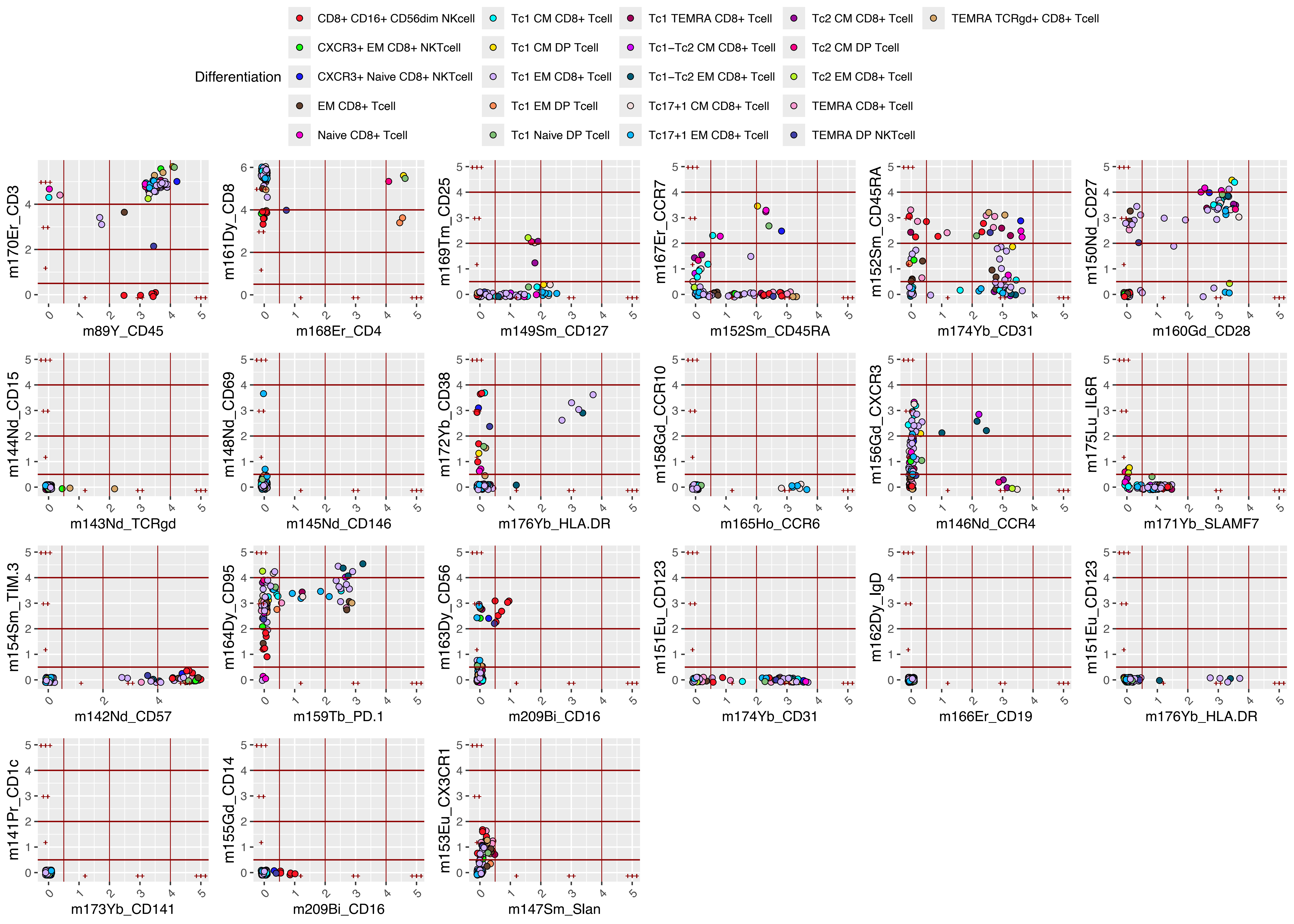
Differentiation
CD8+ CD16+ CD56dim NKcell
CXCR3+ EM CD8+ NKTcell
CXCR3+ Naive CD8+ NKTcell
EM CD8+ Tcell
Naive CD8+ Tcell
Tc1 CM CD8+ Tcell
Tc1 CM DP Tcell
Tc1 EM CD8+ Tcell
Tc1 EM DP Tcell
Tc1 Naive DP Tcell
Tc1 TEMRA CD8+ Tcell
Tc1−Tc2 CM CD8+ Tcell
Tc1−Tc2 EM CD8+ Tcell
Tc17+1 CM CD8+ Tcell
Tc17+1 EM CD8+ Tcell
Tc2 CM CD8+ Tcell
Tc2 CM DP Tcell
Tc2 EM CD8+ Tcell
TEMRA CD8+ Tcell
TEMRA DP NKTcell
TEMRA TCRgd+ CD8+ Tcell
m170Er_CD3
m89Y_CD45
m161Dy_CD8
m168Er_CD4
m169Tm_CD25
m149Sm_CD127
m167Er_CCR7
m152Sm_CD45RA
m152Sm_CD45RA
m174Yb_CD31
m150Nd_CD27
m160Gd_CD28
m144Nd_CD15
m143Nd_TCRgd
m148Nd_CD69
m145Nd_CD146
m172Yb_CD38
m176Yb_HLA.DR
m158Gd_CCR10
m165Ho_CCR6
m156Gd_CXCR3
m146Nd_CCR4
m175Lu_IL6R
m171Yb_SLAMF7
m154Sm_TIM.3
m142Nd_CD57
m164Dy_CD95
m159Tb_PD.1
m163Dy_CD56
m209Bi_CD16
m151Eu_CD123
m174Yb_CD31
m162Dy_IgD
m166Er_CD19
m151Eu_CD123
m176Yb_HLA.DR
m141Pr_CD1c
m173Yb_CD141
m155Gd_CD14
m209Bi_CD16
m153Eu_CX3CR1
m147Sm_Slan

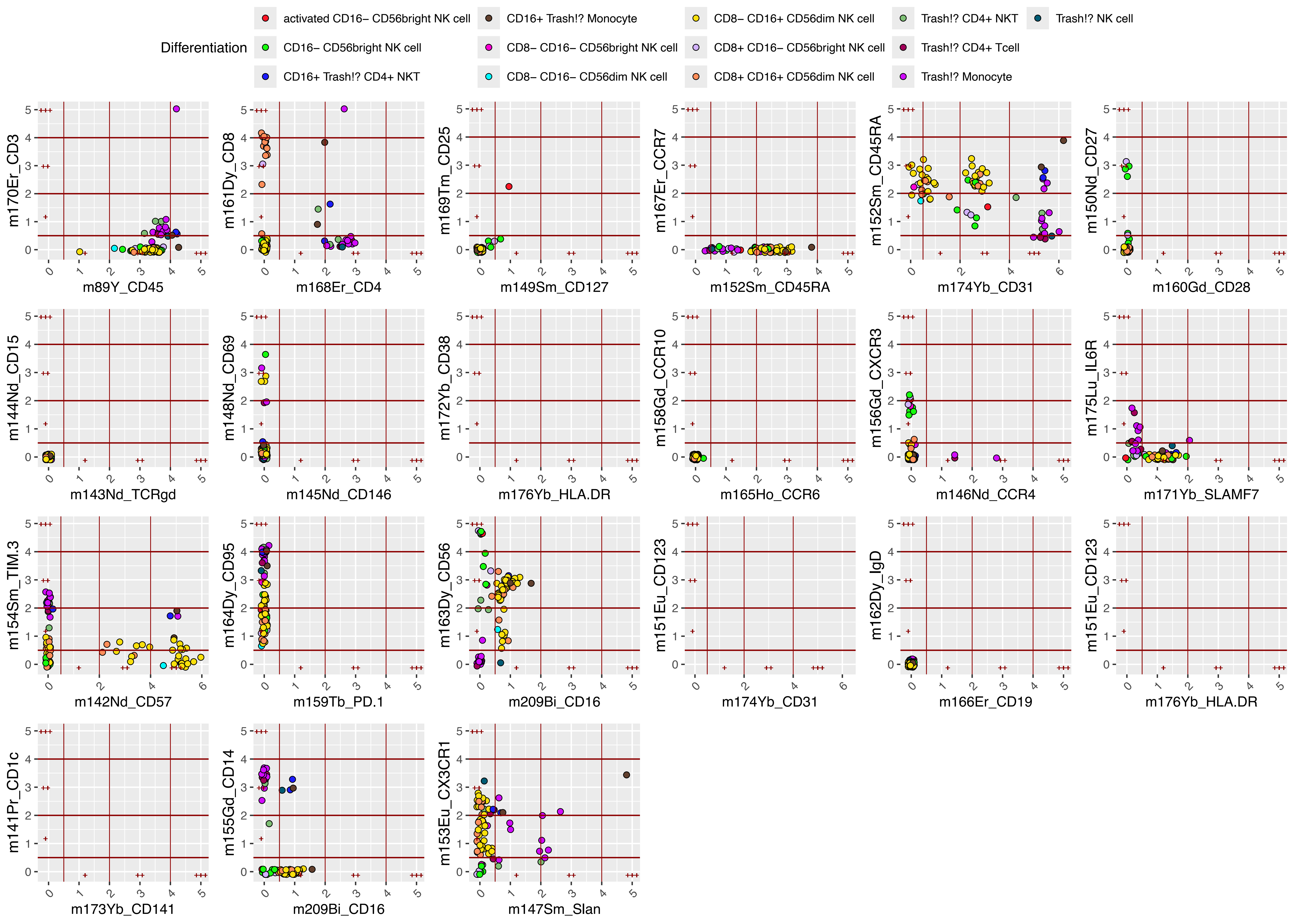

Differentiation
activated CD16– CD56bright NK cell
CD16– CD56bright NK cell
CD16+ Trash!? CD4+ NKT
CD16+ Trash!? Monocyte
CD8– CD16– CD56bright NK cell
CD8– CD16– CD56dim NK cell
CD8– CD16+ CD56dim NK cell
CD8+ CD16– CD56bright NK cell
CD8+ CD16+ CD56dim NK cell
Trash!? CD4+ NKT
Trash!? CD4+ Tcell
Trash!? Monocyte
Trash!? NK cell
m170Er_CD3
m89Y_CD45
m161Dy_CD8
m168Er_CD4
m169Tm_CD25
m149Sm_CD127
m167Er_CCR7
m152Sm_CD45RA
m152Sm_CD45RA
m174Yb_CD31
m150Nd_CD27
m160Gd_CD28
m144Nd_CD15
m143Nd_TCRgd
m148Nd_CD69
m145Nd_CD146
m172Yb_CD38
m176Yb_HLA.DR
m158Gd_CCR10
m165Ho_CCR6
m156Gd_CXCR3
m146Nd_CCR4
m175Lu_IL6R
m171Yb_SLAMF7
m154Sm_TIM.3
m142Nd_CD57
m164Dy_CD95
m159Tb_PD.1
m163Dy_CD56
m209Bi_CD16
m151Eu_CD123
m174Yb_CD31
m162Dy_IgD
m166Er_CD19
m151Eu_CD123
m176Yb_HLA.DR
m141Pr_CD1c
m173Yb_CD141
m155Gd_CD14
m209Bi_CD16
m153Eu_CX3CR1
m147Sm_Slan

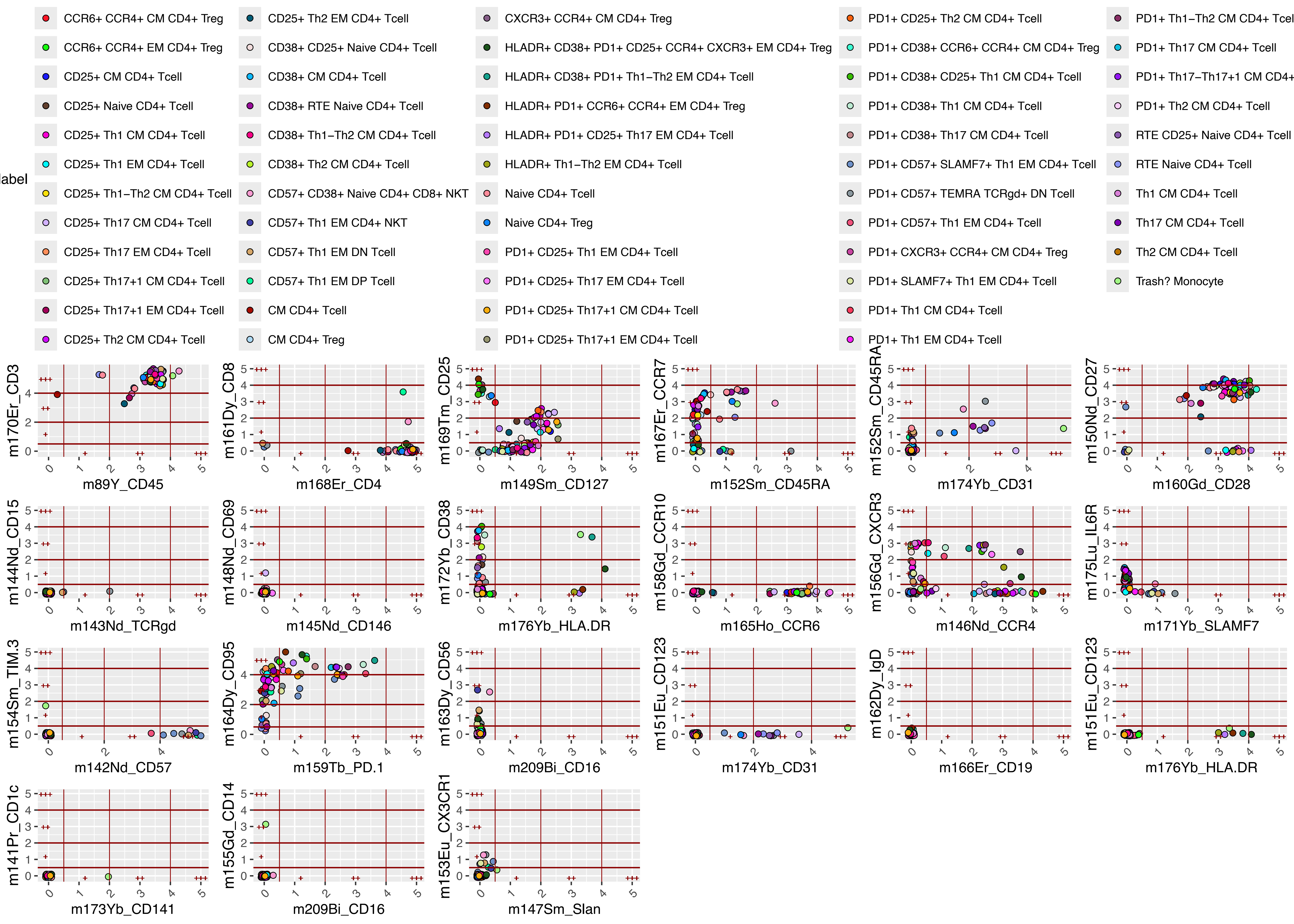
label
CCR6+ CCR4+ CM CD4+ Treg
CCR6+ CCR4+ EM CD4+ Treg
CD25+ CM CD4+ Tcell
CD25+ Naive CD4+ Tcell
CD25+ Th1 CM CD4+ Tcell
CD25+ Th1 EM CD4+ Tcell
CD25+ Th1−Th2 CM CD4+ Tcell
CD25+ Th17 CM CD4+ Tcell
CD25+ Th17 EM CD4+ Tcell
CD25+ Th17+1 CM CD4+ Tcell
CD25+ Th17+1 EM CD4+ Tcell
CD25+ Th2 CM CD4+ Tcell
CD25+ Th2 EM CD4+ Tcell
CD38+ CD25+ Naive CD4+ Tcell
CD38+ CM CD4+ Tcell
CD38+ RTE Naive CD4+ Tcell
CD38+ Th1−Th2 CM CD4+ Tcell
CD38+ Th2 CM CD4+ Tcell
CD57+ CD38+ Naive CD4+ CD8+ NKT
CD57+ Th1 EM CD4+ NKT
CD57+ Th1 EM DN Tcell
CD57+ Th1 EM DP Tcell
CM CD4+ Tcell
CM CD4+ Treg
CXCR3+ CCR4+ CM CD4+ Treg
HLADR+ CD38+ PD1+ CD25+ CCR4+ CXCR3+ EM CD4+ Treg
HLADR+ CD38+ PD1+ Th1−Th2 EM CD4+ Tcell
HLADR+ PD1+ CCR6+ CCR4+ EM CD4+ Treg
HLADR+ PD1+ CD25+ Th17 EM CD4+ Tcell
HLADR+ Th1−Th2 EM CD4+ Tcell
Naive CD4+ Tcell
Naive CD4+ Treg
PD1+ CD25+ Th1 EM CD4+ Tcell
PD1+ CD25+ Th17 EM CD4+ Tcell
PD1+ CD25+ Th17+1 CM CD4+ Tcell
PD1+ CD25+ Th17+1 EM CD4+ Tcell
PD1+ CD25+ Th2 CM CD4+ Tcell
PD1+ CD38+ CCR6+ CCR4+ CM CD4+ Treg
PD1+ CD38+ CD25+ Th1 CM CD4+ Tcell
PD1+ CD38+ Th1 CM CD4+ Tcell
PD1+ CD38+ Th17 CM CD4+ Tcell
PD1+ CD57+ SLAMF7+ Th1 EM CD4+ Tcell
PD1+ CD57+ TEMRA TCRgd+ DN Tcell
PD1+ CD57+ Th1 EM CD4+ Tcell
PD1+ CXCR3+ CCR4+ CM CD4+ Treg
PD1+ SLAMF7+ Th1 EM CD4+ Tcell
PD1+ Th1 CM CD4+ Tcell
PD1+ Th1 EM CD4+ Tcell
PD1+ Th1−Th2 CM CD4+ Tcel
PD1+ Th17 CM CD4+ Tcell
PD1+ Th17−Th17+1 CM CD4+
PD1+ Th2 CM CD4+ Tcell
RTE CD25+ Naive CD4+ Tcell
RTE Naive CD4+ Tcell
Th1 CM CD4+ Tcell
Th17 CM CD4+ Tcell
Th2 CM CD4+ Tcell
Trash? Monocyte
m170Er_CD3 / m89Y_CD45
m161Dy_CD8 / m168Er_CD4
m169Tm_CD25 / m149Sm_CD127
m167Er_CCR7 / m152Sm_CD45RA
m152Sm_CD45RA / m174Yb_CD31
m150Nd_CD27 / m160Gd_CD28
m144Nd_CD15 / m143Nd_TCRgd
m148Nd_CD69 / m145Nd_CD146
m172Yb_CD38 / m176Yb_HLA.DR
m158Gd_CCR10 / m165Ho_CCR6
m156Gd_CXCR3 / m146Nd_CCR4
m175Lu_IL6R / m171Yb_SLAMF7
m154Sm_TIM.3 / m142Nd_CD57
m164Dy_CD95 / m159Tb_PD.1
m163Dy_CD56 / m209Bi_CD16
m151Eu_CD123 / m174Yb_CD31
m162Dy_IgD / m166Er_CD19
m151Eu_CD123 / m176Yb_HLA.DR
m141Pr_CD1c / m173Yb_CD141
m155Gd_CD14 / m209Bi_CD16
m153Eu_CX3CR1 / m147Sm_Slan

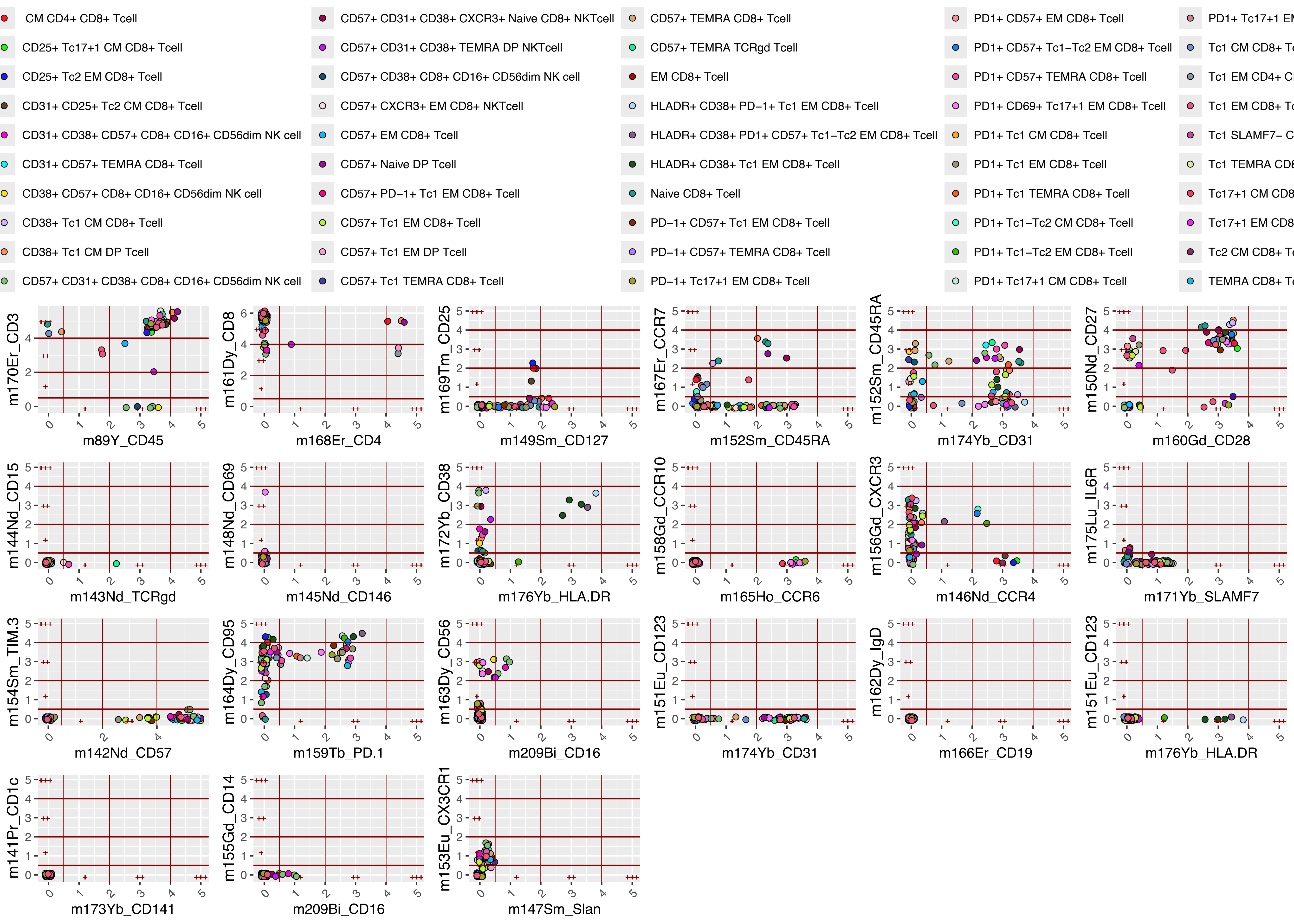
CM CD4+ CD8+ Tcell
CD25+ Tc17+1 CM CD8+ Tcell
CD25+ Tc2 EM CD8+ Tcell
CD31+ CD25+ Tc2 CM CD8+ Tcell
CD31+ CD38+ CD57+ CD8+ CD16+ CD56dim NK cell
CD31+ CD57+ TEMRA CD8+ Tcell
CD38+ CD57+ CD8+ CD16+ CD56dim NK cell
CD38+ Tc1 CM CD8+ Tcell
CD38+ Tc1 CM DP Tcell
CD57+ CD31+ CD38+ CD8+ CD16+ CD56dim NK cell
CD57+ CD31+ CD38+ CXCR3+ Naive CD8+ NKTcell
CD57+ CD31+ CD38+ TEMRA DP NKTcell
CD57+ CD38+ CD8+ CD16+ CD56dim NK cell
CD57+ CXCR3+ EM CD8+ NKTcell
CD57+ EM CD8+ Tcell
CD57+ Naive DP Tcell
CD57+ PD−1+ Tc1 EM CD8+ Tcell
CD57+ Tc1 EM CD8+ Tcell
CD57+ Tc1 EM DP Tcell
CD57+ Tc1 TEMRA CD8+ Tcell
CD57+ TEMRA CD8+ Tcell
CD57+ TEMRA TCRgd Tcell
EM CD8+ Tcell
HLADR+ CD38+ PD−1+ Tc1 EM CD8+ Tcell
HLADR+ CD38+ PD1+ CD57+ Tc1−Tc2 EM CD8+ Tcell
HLADR+ CD38+ Tc1 EM CD8+ Tcell
Naive CD8+ Tcell
PD−1+ CD57+ Tc1 EM CD8+ Tcell
PD−1+ CD57+ TEMRA CD8+ Tcell
PD−1+ Tc17+1 EM CD8+ Tcell
PD1+ CD57+ EM CD8+ Tcell
PD1+ CD57+ Tc1−Tc2 EM CD8+ Tcell
PD1+ CD57+ TEMRA CD8+ Tcell
PD1+ CD69+ Tc17+1 EM CD8+ Tcell
PD1+ Tc1 CM CD8+ Tcell
PD1+ Tc1 EM CD8+ Tcell
PD1+ Tc1 TEMRA CD8+ Tcell
PD1+ Tc1−Tc2 CM CD8+ Tcell
PD1+ Tc1−Tc2 EM CD8+ Tcell
PD1+ Tc17+1 CM CD8+ Tcell
PD1+ Tc17+1 EM
Tc1 CM CD8+ Tc
Tc1 EM CD4+ CD
Tc1 EM CD8+ Tc
Tc1 SLAMF7− C
Tc1 TEMRA CD8
Tc17+1 CM CD8
Tc17+1 EM CD8
Tc2 CM CD8+ Tc
TEMRA CD8+ Tc
m170Er_CD3 / m89Y_CD45
m161Dy_CD8 / m168Er_CD4
m169Tm_CD25 / m149Sm_CD127
m167Er_CCR7 / m152Sm_CD45RA
m152Sm_CD45RA / m174Yb_CD31
m150Nd_CD27 / m160Gd_CD28
m144Nd_CD15 / m143Nd_TCRgd
m148Nd_CD69 / m145Nd_CD146
m172Yb_CD38 / m176Yb_HLA.DR
m158Gd_CCR10 / m165Ho_CCR6
m156Gd_CXCR3 / m146Nd_CCR4
m175Lu_IL6R / m171Yb_SLAMF7
m154Sm_TIM.3 / m142Nd_CD57
m164Dy_CD95 / m159Tb_PD.1
m163Dy_CD56 / m209Bi_CD16
m151Eu_CD123 / m174Yb_CD31
m162Dy_IgD / m166Er_CD19
m151Eu_CD123 / m176Yb_HLA.DR
m141Pr_CD1c / m173Yb_CD141
m155Gd_CD14 / m209Bi_CD16
m153Eu_CX3CR1 / m147Sm_Slan

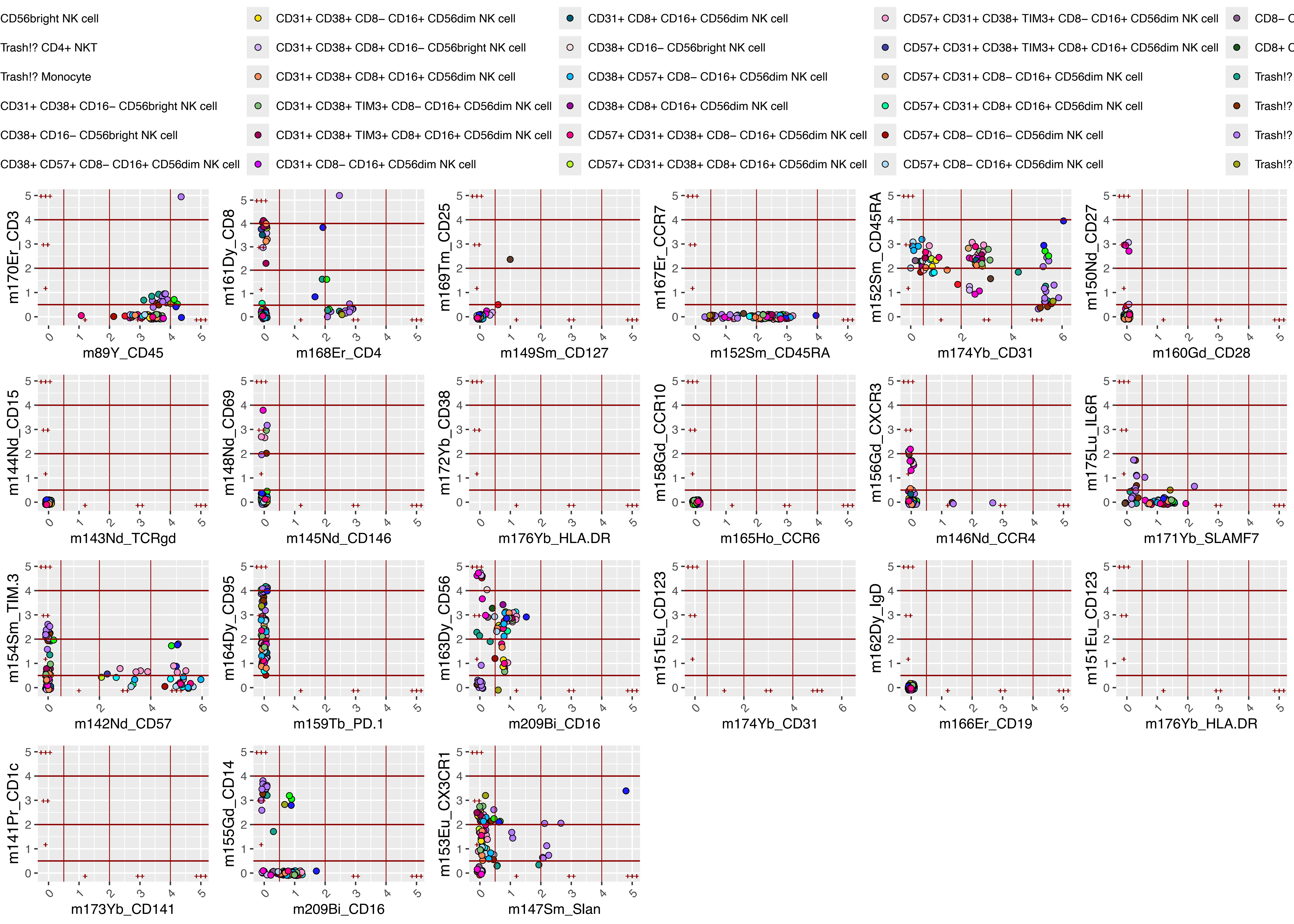
CD56bright NK cell
Trash!? CD4+ NKT
Trash!? Monocyte
CD31+ CD38+ CD16− CD56bright NK cell
CD38+ CD16− CD56bright NK cell
CD38+ CD57+ CD8− CD16+ CD56dim NK cell
CD31+ CD38+ CD8− CD16+ CD56dim NK cell
CD31+ CD38+ CD8+ CD16− CD56bright NK cell
CD31+ CD38+ CD8+ CD16+ CD56dim NK cell
CD31+ CD38+ TIM3+ CD8− CD16+ CD56dim NK cell
CD31+ CD38+ TIM3+ CD8+ CD16+ CD56dim NK cell
CD31+ CD8− CD16+ CD56dim NK cell
CD31+ CD8+ CD16+ CD56dim NK cell
CD38+ CD16− CD56bright NK cell
CD38+ CD57+ CD8− CD16+ CD56dim NK cell
CD38+ CD8+ CD16+ CD56dim NK cell
CD57+ CD31+ CD38+ CD8− CD16+ CD56dim NK cell
CD57+ CD31+ CD38+ CD8+ CD16+ CD56dim NK cell
CD57+ CD31+ CD38+ TIM3+ CD8− CD16+ CD56dim NK cell
CD57+ CD31+ CD38+ TIM3+ CD8+ CD16+ CD56dim NK cell
CD57+ CD31+ CD8− CD16+ CD56dim NK cell
CD57+ CD31+ CD8+ CD16+ CD56dim NK cell
CD57+ CD8− CD16− CD56dim NK cell
CD57+ CD8− CD16+ CD56dim NK cell
CD8−
CD8+
Trash!?
Trash!?
Trash!?
Trash!?
m170Er_CD3
m89Y_CD45
m161Dy_CD8
m168Er_CD4
m169Tm_CD25
m149Sm_CD127
m167Er_CCR7
m152Sm_CD45RA
m152Sm_CD45RA
m174Yb_CD31
m150Nd_CD27
m160Gd_CD28
m144Nd_CD15
m143Nd_TCRgd
m148Nd_CD69
m145Nd_CD146
m172Yb_CD38
m176Yb_HLA.DR
m158Gd_CCR10
m165Ho_CCR6
m156Gd_CXCR3
m146Nd_CCR4
m175Lu_IL6R
m171Yb_SLAMF7
m154Sm_TIM.3
m142Nd_CD57
m164Dy_CD95
m159Tb_PD.1
m163Dy_CD56
m209Bi_CD16
m151Eu_CD123
m174Yb_CD31
m162Dy_IgD
m166Er_CD19
m151Eu_CD123
m176Yb_HLA.DR
m141Pr_CD1c
m173Yb_CD141
m155Gd_CD14
m209Bi_CD16
m153Eu_CX3CR1
m147Sm_Slan
